# Variation in *Botryosphaeriaceae* from *Eucalyptus* plantations in YunNan Province in southwestern China across a climatic gradient

**DOI:** 10.1186/s43008-020-00043-x

**Published:** 2020-10-15

**Authors:** Guoqing Li, Bernard Slippers, Michael J. Wingfield, Shuaifei Chen

**Affiliations:** 1 State Key Laboratory of Tree Genetics and Breeding (SKLTGB), Chinese Academy of Forestry (CAF), Haidian District, Beijing, 100091 China; 2 Department of Biochemistry, Genetics and Microbiology, Forestry and Agricultural Biotechnology Institute (FABI), University of Pretoria, Pretoria, 0028 South Africa; 3 China Eucalypt Research Centre (CERC), Chinese Academy of Forestry (CAF), ZhanJiang, 524022 GuangDong Province China

**Keywords:** *Botryosphaeria*, *Lasiodiplodia*, *Neofusicoccum*, Pathogenicity, Phylogeny, Taxonomy

## Abstract

The *Botryosphaeriaceae* accommodates many important pathogens of woody plants, including *Eucalyptus*. Recently, *Botryosphaeriaceae* were isolated from diseased plant parts from surveys of *Eucalyptus* plantations in the YunNan Province, China. The aims of this study were to identify these *Botryosphaeriaceae* isolates and to evaluate their pathogenicity to *Eucalyptus*. A total of 166 isolates of *Botryosphaeriaceae* were obtained from six regions in the YunNan Province, of which 76 were from *Eucalyptus urophylla* × *E. grandis* hybrids, 49 from *E. globulus* trees, and 41 isolates were from other unknown *Eucalyptus* species or hybrids. Isolates were identified by comparing DNA sequences of the internal transcribed spacer ribosomal RNA locus (ITS), partial translation elongation factor 1-alpha (*tef1*), β-tubulin 2 (*tub2*) and DNA-directed RNA polymerase II subunit (*rpb2*) genes, and combined with their morphological characteristics. Eleven species were identified, including *Botryosphaeria fusispora*, *B. wangensis*, *Lasiodiplodia pseudotheobromae*, *Neofusicoccum kwambonambiense*, *N. parvum*, and six novel species described as *B. puerensis*, *N. dianense*, *N. magniconidium*, *N. ningerense*, *N. parviconidium* and *N. yunnanense*. The dominant species across the regions were *N. yunnanense*, *N. parvum* and *B. wangensis*, representing 31.3, 25.3 and 19.9% of the total isolates, respectively. Species diversity and composition changed across the different climatic zones, despite their relatively close geographic proximity and the fact that some of the species have a global distribution. All the *Botryosphaeriaceae* species were pathogenic to one-year-old plants of an *E. urophylla* × *E. grandis* clone and *E. globulus* seed-derived plants, but showed significant inter- and intra-species variation in aggressiveness amongst isolates. The study provides a foundation for monitoring and management of *Botryosphaeriaceae* through selection and breeding of *Eucalyptus* in the YunNan Province of southwestern China.

## INTRODUCTION

*Eucalyptus* species have been widely planted in many countries of the world for wood and fibre needs, mostly due to their rapid growth and adaptability to a variety of ecological conditions (Coppen 2002). In China, with more than 4.5 million hectares of *Eucalyptus* planted, an important area for *Eucalyptus* plantation establishment is the YunNan Province (Xie et al. 2017). This province includes seven climatic zones due to variation in altitude. These include a cold highland zone (T1), central temperate zone (T2), southern temperate zone (T3), northern sub-tropical zone (T4), central sub-tropical zone (T5), southern sub-tropical zone (T6) and tropical zone (T7) (Ye 2017). Most *Eucalyptus* have been planted in the sub-tropical and tropical (T4–T7), central and southern parts of the YunNan Province. The *Eucalyptus* species planted include large areas of *E. urophylla* × *E. grandis* hybrids and *E. globulus*, and smaller areas of *E. nitens* and *E. smithii* (Qi 2002).

In recent years, *Eucalyptus* plantations in China have faced significant health threats from different pathogens, including species in the *Botryosphaeriaceae* (Chen et al. 2011), *Cryphonectriaceae* (Chen et al. 2010; Wang et al. 2018) and *Teratosphaeriaceae* (Burgess et al. 2006a), as well as *Botrytis* (Liu et al. 2016), *Calonectria* (Lombard et al. 2010; Li et al. 2017), *Ceratocystis* (Chen et al. 2013), *Quambalaria* (Zhou et al. 2007; Chen et al. 2017) and *Ralstonia* (Carstensen et al. 2017). Of these, *Botryosphaeriaceae* are amongst the most widespread and common associated with *Eucalyptus* plantations in southern China (Chen et al. 2011; Li et al. 2018).

Diseases associated with *Botryosphaeriaceae* have been reported on a variety of woody plants globally (Slippers and Wingfield 2007; Dissanayake et al. 2016; Mehl et al. 2017; Slippers et al. 2017). They usually occur when plants are subjected to environmental stresses, including drought, frost, physical damage and biological stress (Old et al. 2003; Slippers and Wingfield 2007; Manawasinghe et al. 2016). Typical symptoms associated with *Botryosphaeriaceae* infections include die-back, canker, shoot blight, and fruit rot (Slippers and Wingfield 2007; Slippers et al. 2017; Billones-Baaijens and Savocchia 2019). On *Eucalyptus* in China, the *Botryosphaeriaceae* has been associated with stem cankers as well as shoot and twig blights.

The taxonomic status of *Botryosphaeriaceae* has been substantially revised in recent years and now includes 23 genera and at least 200 species known from culture (Liu et al. 2012; Phillips et al. 2013; Dissanayake et al. 2016; Slippers et al. 2017; Yang et al. 2017; Jayawardena et al. 2019a, 2019b). These species include many cryptic taxa and require DNA sequence-based identification, often considering sequence data from multiple loci. Recent studies on the *Botryosphaeriaceae* from *Eucalyptus* in China that have been based on DNA sequence data have identified twelve species. These include *Botryosphaeria dothidea*, *B. fabicerciana*, *B. fusispora*, *B. pseudoramosa*, *B. qingyuanensis*, *Lasiodiplodia brasiliense*, *L. pseudotheobromae*, *L. theobromae*, *Neofusicoccum microconidium*, *N. parvum*, *N. ribis* sensu lato and *N. sinoeucalypti* (Yu et al. 2009; Chen et al. 2011; Li et al. 2015, 2018). These studies have, however, not included thorough sampling from *Eucalyptus* in the YunNan Province.

During disease surveys in *Eucalyptus* plantations in the YunNan Province in 2014, typical disease symptoms linked to the *Botryosphaeriaceae* were observed. The aims of this study were to (1) identify the species of *Botryosphaeriaceae* isolated from diseased *Eucalyptus* trees in YunNan Province based on phylogenetic inference combined with morphological characteristics, (2) determine their geographic distribution in different regions of this province, and (3) evaluate their pathogenicity on one-year-old plants of an *E. urophylla* × *E. grandis* hybrid clone and *E. globulus* seed-derived plants.

## MATERIALS AND METHODS

### Sample collection and fungal isolation

**Fig. 1:**
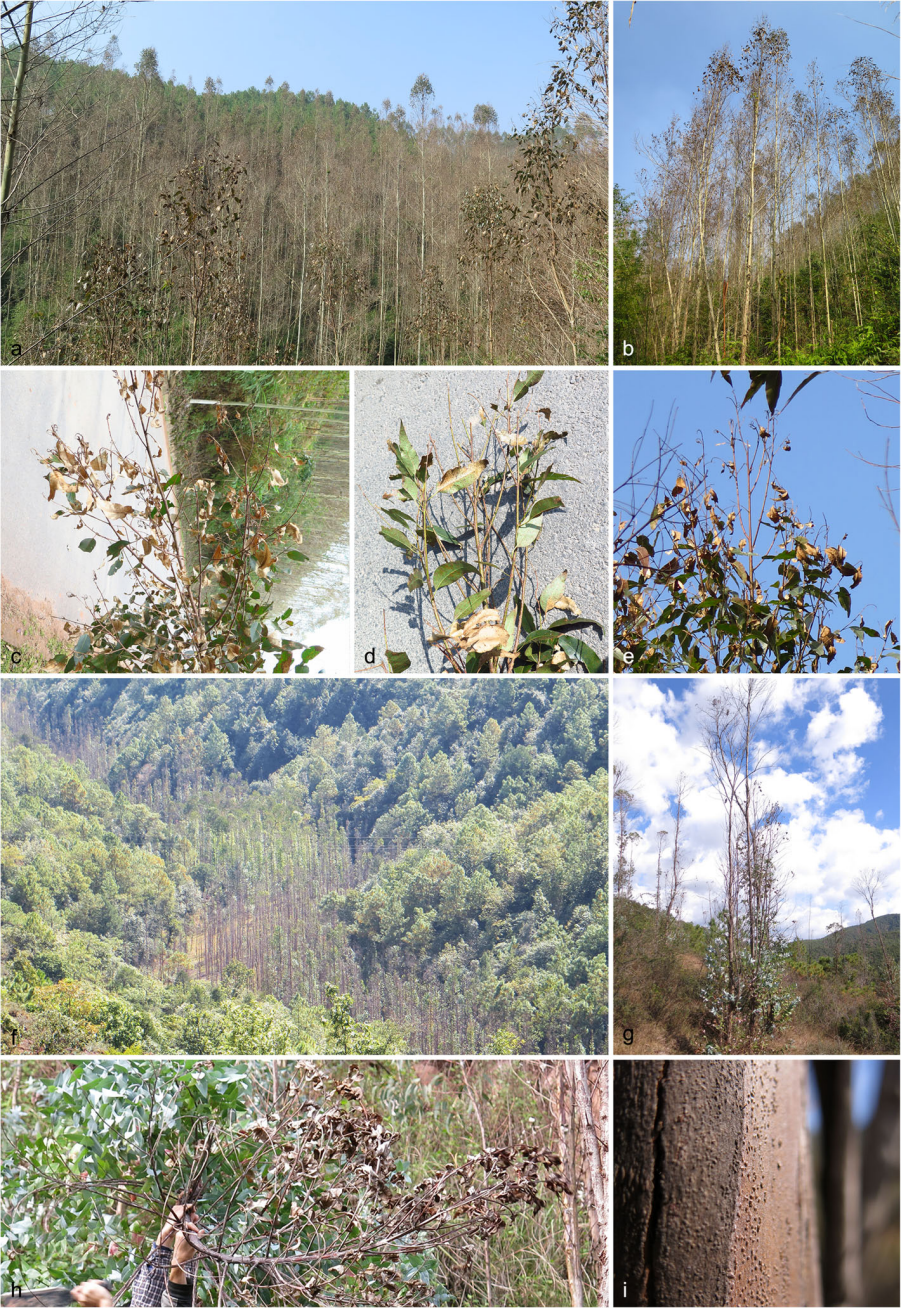
Disease symptoms on *Eucalyptus* trees associate with *Botryosphaeriaceae* in YunNan Province. **a, b**. die-back of *E. urophylla × E. grandis* hybrids; **c–e**. branch and twig blight of *E. urophylla × E. grandis* hybrids. **f–h**. die-back of *E. globulus*; **i**. fruiting structures on an *E. globulus* stem

Field surveys of *Eucalyptus* plantations were conducted in YunNan Province of southwestern China during 2014. A large area of these *Eucalyptus* plantations was severely damaged by disease with symptoms typical of the *Botryosphaeriaceae*. These symptoms included die-back, leaf and shoot blight, stem and branch canker, and they resulted in tree death in some plantations (Fig. 1).

Stems, branches and twigs from *Eucalyptus* trees showing typical symptoms of *Botryosphaeriaceae* infection were collected. *Botryosphaeriaceae* isolates were obtained as described in Li et al. (2018). All cultures were deposited in the Culture Collection (CSF) of the China Eucalypt Research Centre (CERC), Chinese Academy of Forestry (CAF), ZhanJiang, GuangDong Province, China. Duplicate cultures were deposited in the culture collection (CMW) of the Forestry and Agricultural Biotechnology Institute (FABI), University of Pretoria, Pretoria, South Africa, and representative cultures were deposited in the China General Microbiological Culture Collection Center (CGMCC), Beijing, China. The dried specimens were deposited in the mycological fungarium of the Institute of Microbiology, Chinese Academy of Sciences (HMAS), Beijing, China.

### DNA extraction, PCR amplification and sequencing

Total DNA of each isolate was extracted from the mycelium of 7-day-old cultures using the CTAB method as described in van Burik et al. (1998). RNA from each DNA sample was removed by adding 2 mL RNase A (10 mg/mL) and incubating at 37 °C for 1 h. Quality and quantity of the DNA samples were determined using a NanoDrop 2000 Spectrometer (Thermo Fisher Scientific Inc. Waltham, MA, USA), and each DNA sample was diluted to approximately 100 ng/uL with DNase/RNase-free ddH_2_O (Sangon Biotech Co., Ltd., Shanghai, China) for PCR amplification. Three to four loci were amplified, including the internal transcribed spacer (ITS), a part of the translation elongation factor 1-alpha (*tef1*), a part of the β-tubulin 2 (*tub2*) and a part of DNA directed RNA polymerase II subunit (*rpb2*). Details regarding primers, PCR reactions and cycling conditions were as described by Li et al. (2018). Primers were synthesised and PCR products were sequenced by the Beijing Genomics Institute (BGI), GuangZhou, GuangDong Province, China. Sequences obtained in this study were all deposited in GenBank (http://www.ncbi.nlm.nih.gov) (Table 1).

**Table 1:** Isolates sequenced and used for phylogenetic analyses, morphological studies and pathogenicity tests in this study

Identity^a^	Genotype^b^	Isolate No.^c^	Host	Location	GPS information	Collector	GenBank accession No.^d^			
							ITS	*tef1*	*tub2*	*rpb2*
*Botryosphaeria fusispora*	AAAAAA	CSF6021^f^	*E. urophylla* × *E. grandis*	NingEr County, PuEr Region, YunNan Province, China	23°05′26″N, 102°02′40″E	S.F. Chen & G.Q. Li	MT028551	MT028717	MT028883	MT029049
	AAAAAA	CSF6056^f^	*E. urophylla* × *E. grandis*	JingGu County, PuEr Region, YunNan Province, China	23°23′58″N, 100°50′37″E	S.F. Chen & G.Q. Li	MT028552	MT028718	MT028884	MT029050
	AAAAAA	CSF6160^h^	*E. globulus*	LuFeng County, ChuXiong Region, YunNan Province, China	25°03′12″N, 101°46′29″E	S.F. Chen & G.Q. Li	MT028553	MT028719	MT028885	MT029051
	AAAABA	CSF5683^f^	*Eucalyptus* sp.	PingBian County, HongHe Region, YunNan Province, China	23°00′52″N, 103°38′09″E	S.F. Chen & G.Q. Li	MT028554	MT028720	MT028886	MT029052
	AAA-AA	CSF5852	*Eucalyptus* sp.	MengZi County, HongHe Region, YunNan Province, China	23°12′24″N, 103°30′58″E	S.F. Chen & G.Q. Li	MT028555	MT028721	MT028887	N/A
	AAA-AA	CSF5950^h^	*E. urophylla* × *E. grandis*	YuanJiang County, YuXi Region, YunNan Province, China	23°29′04″N, 102°07′34″E	S.F. Chen & G.Q. Li	MT028556	MT028722	MT028888	N/A
	AAA---	CSF6162	*E. globulus*	LuFeng County, ChuXiong Region, YunNan Province, China	25°03′12″N, 101°46′29″E	S.F. Chen & G.Q. Li	MT028557	MT028723	MT028889	N/A
	AAA---	CSF6066	*E. urophylla* × *E. grandis*	JingGu County, PuEr Region, YunNan Province, China	23°23′58″N, 100°50′37″E	S.F. Chen & G.Q. Li	MT028558	MT028724	MT028890	N/A
	AAA---	CSF5957	*E. urophylla* × *E. grandis*	YuanJiang County, YuXi Region, YunNan Province, China	23°29′04″N, 102°07′34″E	S.F. Chen & G.Q. Li	MT028559	MT028725	MT028891	N/A
	AAA---	CSF5964	*E. urophylla* × *E. grandis*	YuanJiang County, YuXi Region, YunNan Province, China	23°29′04″N, 102°07′34″E	S.F. Chen & G.Q. Li	MT028560	MT028726	MT028892	N/A
	AAA---	CSF5976	*E. urophylla* × *E. grandis*	YuanJiang County, YuXi Region, YunNan Province, China	23°29′04″N, 102°07′34″E	S.F. Chen & G.Q. Li	MT028561	MT028727	MT028893	N/A
	ABABAA	CSF5871^f,h^	*E. urophylla* × *E. grandis*	PingBian County, HongHe Region, YunNan Province, China	23°12′24″N, 103°30′58″E	S.F. Chen & G.Q. Li	MT028562	MT028728	MT028894	MT029053
	ABABAA	CSF5872^f^	*E. urophylla* × *E. grandis*	PingBian County, HongHe Region, YunNan Province, China	23°12′24″N, 103°30′58″E	S.F. Chen & G.Q. Li	MT028563	MT028729	MT028895	MT029054
	ACAAAA	CSF6178^f,h^	*E. globulus*	AnNing County, KunMing Region, YunNan Province, China	24°55′02″N, 102°23′41″E	S.F. Chen & G.Q. Li	MT028564	MT028730	MT028896	MT029055
	ACAAAA	CSF6063^f,h^	*E. urophylla* × *E. grandis*	JingGu County, PuEr Region, YunNan Province, China	23°23′58″N, 100°50′37″E	S.F. Chen & G.Q. Li	MT028565	MT028731	MT028897	MT029056
	ACA---	CSF6179	*E. globulus*	AnNing County, KunMing Region, YunNan Province, China	24°55′02″N, 102°23′41″E	S.F. Chen & G.Q. Li	MT028566	MT028732	MT028898	N/A
	ACA---	CSF6180	*E. globulus*	AnNing County, KunMing Region, YunNan Province, China	24°55′02″N, 102°23′41″E	S.F. Chen & G.Q. Li	MT028567	MT028733	MT028899	N/A
	ACA---	CSF6181	*E. globulus*	AnNing County, KunMing Region, YunNan Province, China	24°55′02″N, 102°23′41″E	S.F. Chen & G.Q. Li	MT028568	MT028734	MT028900	N/A
***B. puerensis***	AAAAAA	CSF6052 = CGMCC3.20081^e,f,g,h^	*E. urophylla* × *E. grandis*	JingGu County, PuEr Region, YunNan Province, China	23°20′21″N, 100°54′38″E	S.F. Chen & G.Q. Li	MT028569	MT028735	MT028901	MT029057
*B. wangensis*	AAAAAA	CSF5737	*Eucalyptus* sp.	PingBian County, HongHe Region, YunNan Province, China	23°08′00″N, 103°32′39″E	S.F. Chen & G.Q. Li	MT028570	MT028736	MT028902	MT029058
	AAAAAA	CSF5770^f,h^	*E. globulus*	MengZi County, HongHe Region, YunNan Province, China	23°14′27″N, 103°28′59″E	S.F. Chen & G.Q. Li	MT028571	MT028737	MT028903	MT029059
	AAAAAA	CSF5980^f,h^	*E. urophylla* × *E. grandis*	YuanJiang County, YuXi Region, YunNan Province, China	23°29′04″N, 102°07′34″E	S.F. Chen & G.Q. Li	MT028572	MT028738	MT028904	MT029060
	AAAAAA	CSF6158	*E. globulus*	LuFeng County, ChuXiong Region, YunNan Province, China	25°03′12″N, 101°46′29″E	S.F. Chen & G.Q. Li	MT028574	MT028740	MT028906	MT029062
	AAABBA	CSF6113^f^	*E. globulus*	ChuXiong County, ChuXiong Region, YunNan Province, China	25°02′48″N, 101°41′46″E	S.F. Chen & G.Q. Li	MT028573	MT028739	MT028905	MT029061
	AAA---	CSF6133	*E. globulus*	ChuXiong County, ChuXiong Region, YunNan Province, China	25°02′48″N, 101°41′46″E	S.F. Chen & G.Q. Li	MT028575	MT028741	MT028907	N/A
	AAA---	CSF6159	*E. globulus*	LuFeng County, ChuXiong Region, YunNan Province, China	25°03′12″N, 101°46′29″E	S.F. Chen & G.Q. Li	MT028576	MT028742	MT028908	N/A
	AAA---	CSF5776	*E. globulus*	MengZi County, HongHe Region, YunNan Province, China	23°14′27″N, 103°28′59″E	S.F. Chen & G.Q. Li	MT028577	MT028743	MT028909	N/A
	AAA---	CSF5812	*Eucalyptus* sp.	MengZi County, HongHe Region, YunNan Province, China	23°12′24″N, 103°30′58″E	S.F. Chen & G.Q. Li	MT028578	MT028744	MT028910	N/A
	AAA---	CSF5830	*Eucalyptus* sp.	MengZi County, HongHe Region, YunNan Province, China	23°12′24″N, 103°30′58″E	S.F. Chen & G.Q. Li	MT028579	MT028745	MT028911	N/A
	AAA---	CSF5850	*Eucalyptus* sp.	MengZi County, HongHe Region, YunNan Province, China	23°12′24″N, 103°30′58″E	S.F. Chen & G.Q. Li	MT028580	MT028746	MT028912	N/A
	AAA---	CSF5741	*Eucalyptus* sp.	MengZi County, HongHe Region, YunNan Province, China	23°08′00″N, 103°32′39″E	S.F. Chen & G.Q. Li	MT028581	MT028747	MT028913	N/A
	AAA---	CSF5923	*E. urophylla* × *E. grandis*	PingBian County, HongHe Region, YunNan Province, China	23°09′26″N, 103°32′14″E	S.F. Chen & G.Q. Li	MT028582	MT028748	MT028914	N/A
	ABAAAA	CSF6173^f,h^	*E. globulus*	AnNing County, KunMing Region, YunNan Province, China	24°55′02″N, 102°23′41″E	S.F. Chen & G.Q. Li	MT028583	MT028749	MT028915	MT029063
	ABAAAA	CSF6174^f^	*E. globulus*	AnNing County, KunMing Region, YunNan Province, China	24°55′02″N, 102°23′41″E	S.F. Chen & G.Q. Li	MT028584	MT028750	MT028916	MT029064
	ACAAAA	CSF6237^f,h^	*E. urophylla* × *E. grandis*	FuNing County, WenShan Region, YunNan Province, China	23°36′26″N, 105°40′43″E	S.F. Chen	MT028585	MT028751	MT028917	MT029065
	ADACAA	CSF6242	*E. urophylla* × *E. grandis*	FuNing County, WenShan Region, YunNan Province, China	23°36′26″N, 105°40′43″E	S.F. Chen	MT028586	MT028752	MT028918	MT029066
	ADACAA	CSF5781^f,h^	*E. globulus*	MengZi County, HongHe Region, YunNan Province, China	23°14′27″N, 103°28′59″E	S.F. Chen & G.Q. Li	MT028587	MT028753	MT028919	MT029067
	ADACAA	CSF5878^f,h^	*E. urophylla* × *E. grandis*	PingBian County, HongHe Region, YunNan Province, China	23°08′02″N, 103°32′29″E	S.F. Chen & G.Q. Li	MT028588	MT028754	MT028920	MT029068
	ADACAA	CSF5971	*E. urophylla* × *E. grandis*	YuanJiang County, YuXi Region, YunNan Province, China	23°29′04″N, 102°07′34″E	S.F. Chen & G.Q. Li	MT028589	MT028755	MT028921	MT029069
	ADA---	CSF6243	*E. urophylla* × *E. grandis*	FuNing County, WenShan Region, YunNan Province, China	23°36′26″N, 105°40′43″E	S.F. Chen	MT028590	MT028756	MT028922	N/A
	ADA---	CSF5847	*Eucalyptus* sp.	MengZi County, HongHe Region, YunNan Province, China	23°12′24″N, 103°30′58″E	S.F. Chen & G.Q. Li	MT028591	MT028757	MT028923	N/A
	ADA---	CSF5890	*E. urophylla* × *E. grandis*	PingBian County, HongHe Region, YunNan Province, China	23°09′26″N, 103°32′14″E	S.F. Chen & G.Q. Li	MT028592	MT028758	MT028924	N/A
	ADA---	CSF5895	*E. urophylla* × *E. grandis*	PingBian County, HongHe Region, YunNan Province, China	23°09′26″N, 103°32′14″E	S.F. Chen & G.Q. Li	MT028593	MT028759	MT028925	N/A
	ADA---	CSF5972	*E. urophylla* × *E. grandis*	YuanJiang County, YuXi Region, YunNan Province, China	23°29′04″N, 102°07′34″E	S.F. Chen & G.Q. Li	MT028594	MT028760	MT028926	N/A
	BAAAAA	CSF6235	*E. urophylla* × *E. grandis*	FuNing County, WenShan Region, YunNan Province, China	23°36′26″N, 105°40′43″E	S.F. Chen	MT028595	MT028761	MT028927	MT029070
	BAAAAA	CSF5868^f,h^	*E. urophylla* × *E. grandis*	MengZi County, HongHe Region, YunNan Province, China	23°12′24″N, 103°30′58″E	S.F. Chen & G.Q. Li	MT028596	MT028762	MT028928	MT029071
	BAAAAA	CSF5944	*E. urophylla* × *E. grandis*	YuanJiang County, YuXi Region, YunNan Province, China	23°29′04″N, 102°07′34″E	S.F. Chen & G.Q. Li	MT028597	MT028763	MT028929	MT029072
	BAAAAA	CSF5733^f^	*Eucalyptus* sp.	PingBian County, HongHe Region, YunNan Province, China	23°08′00″N, 103°32′39″E	S.F. Chen & G.Q. Li	MT028598	MT028764	MT028930	MT029073
	BAA---	CSF5948	*E. urophylla* × *E. grandis*	YuanJiang County, YuXi Region, YunNan Province, China	23°29′04″N, 102°07′34″E	S.F. Chen & G.Q. Li	MT028599	MT028765	MT028931	N/A
	BAA---	CSF5969	*E. urophylla* × *E. grandis*	YuanJiang County, YuXi Region, YunNan Province, China	23°29′04″N, 102°07′34″E	S.F. Chen & G.Q. Li	MT028600	MT028766	MT028932	N/A
	CAAAAA	CSF5820^f,h^	*Eucalyptus* sp.	MengZi County, HongHe Region, YunNan Province, China	23°12′24″N, 103°30′58″E	S.F. Chen & G.Q. Li	MT028601	MT028767	MT028933	MT029074
	CAAAAA	CSF5838^f^	*Eucalyptus* sp.	MengZi County, HongHe Region, YunNan Province, China	23°12′24″N, 103°30′58″E	S.F. Chen & G.Q. Li	MT028602	MT028768	MT028934	MT029075
*Lasiodiplodia pseudotheobromae*	AAAAAA	CSF6050^f,h^	*E. urophylla* × *E. grandis*	JingGu County, PuEr Region, YunNan Province, China	23°20′21″N, 100°54′38″E	S.F. Chen & G.Q. Li	MT028603	MT028769	MT028935	MT029076
	AAAAAA	CSF5802^f,h^	*Eucalyptus* sp.	PingBian County, HongHe Region, YunNan Province, China	23°10′07″N, 103°32′27″E	S.F. Chen & G.Q. Li	MT028604	MT028770	MT028936	MT029077
***Neofusicoccum dianense***	AAAAAA	CSF6075 = CGMCC3.20082^e,f,g,h^	*E. urophylla* × *E. grandis*	JingGu County, PuEr Region, YunNan Province, China	23°23′58″N, 100°50′37″E	S.F. Chen & G.Q. Li	MT028605	MT028771	MT028937	MT029078
	AAAAAA	CSF5840	*Eucalyptus* sp.	MengZi County, HongHe Region, YunNan Province, China	23°12′24″N, 103°30′58″E	S.F. Chen & G.Q. Li	MT028606	MT028772	MT028938	MT029079
	AAAAAA	CSF5841	*Eucalyptus* sp.	MengZi County, HongHe Region, YunNan Province, China	23°12′24″N, 103°30′58″E	S.F. Chen & G.Q. Li	MT028607	MT028773	MT028939	MT029080
	AAAAAA	CSF5721 = CGMCC3.20075^f,g,h^	*E. globulus*	PingBian County, HongHe Region, YunNan Province, China	23°05′36″N, 103°31′52″E	S.F. Chen & G.Q. Li	MT028608	MT028774	MT028940	MT029081
	BAAABA	CSF5722^f,h^	*E. globulus*	PingBian County, HongHe Region, YunNan Province, China	23°05′36″N, 103°31′52″E	S.F. Chen & G.Q. Li	MT028609	MT028775	MT028941	MT029082
*N. kwambonambiense*	AAAAAA	CSF6037^f,h^	*E. urophylla* × *E. grandis*	NingEr County, PuEr Region, YunNan Province, China	23°05′26″N, 102°02′40″E	S.F. Chen & G.Q. Li	MT028610	MT028776	MT028942	MT029083
***N. magniconidium***	AAAAAA	CSF5875 = CGMCC3.20076^f,g,h^	*E. urophylla* × *E. grandis*	PingBian County, HongHe Region, YunNan Province, China	23°08′02″N, 103°32′29″E	S.F. Chen & G.Q. Li	MT028611	MT028777	MT028943	MT029084
	AAAAAA	CSF5876 = CGMCC3.20077^e,f,g,h^	*E. urophylla* × *E. grandis*	PingBian County, HongHe Region, YunNan Province, China	23°08′02″N, 103°32′29″E	S.F. Chen & G.Q. Li	MT028612	MT028778	MT028944	MT029085
***N. ningerense***	AAAAAA	CSF6028^f,g,h^	*E. urophylla* × *E. grandis*	NingEr County, PuEr Region, YunNan Province, China	23°05′26″N, 102°02′40″E	S.F. Chen & G.Q. Li	MT028613	MT028779	MT028945	MT029086
	AAAAAA	CSF6030^f,g,h^	*E. urophylla* × *E. grandis*	NingEr County, PuEr Region, YunNan Province, China	23°05′26″N, 102°02′40″E	S.F. Chen & G.Q. Li	MT028614	MT028780	MT028946	MT029087
***N. parviconidium***	AAAAAA	CSF5667 = CGMCC3.20074^e,f,g,h^	*Eucalyptus* sp.	PingBian County, HongHe Region, YunNan Province, China	23°00′52″N, 103°38′09″E	S.F. Chen & G.Q. Li	MT028615	MT028781	MT028947	MT029088
	AAAAAA	CSF5670	*Eucalyptus* sp.	PingBian County, HongHe Region, YunNan Province, China	23°00′52″N, 103°38′09″E	S.F. Chen & G.Q. Li	MT028616	MT028782	MT028948	MT029089
	AAAAAA	CSF5671	*Eucalyptus* sp.	PingBian County, HongHe Region, YunNan Province, China	23°00′52″N, 103°38′09″E	S.F. Chen & G.Q. Li	MT028617	MT028783	MT028949	MT029090
	AAAAAA	CSF5672	*Eucalyptus* sp.	PingBian County, HongHe Region, YunNan Province, China	23°00′52″N, 103°38′09″E	S.F. Chen & G.Q. Li	MT028618	MT028784	MT028950	MT029091
	AAAAAA	CSF5677 = CGMCC3.20085^f,g,h^	*Eucalyptus* sp.	PingBian County, HongHe Region, YunNan Province, China	23°00′52″N, 103°38′09″E	S.F. Chen & G.Q. Li	MT028619	MT028785	MT028951	MT029092
	AAAAAA	CSF5678	*Eucalyptus* sp.	PingBian County, HongHe Region, YunNan Province, China	23°00′52″N, 103°38′09″E	S.F. Chen & G.Q. Li	MT028620	MT028786	MT028952	MT029093
	AAAAAA	CSF5681^g,h^	*Eucalyptus* sp.	PingBian County, HongHe Region, YunNan Province, China	23°00′52″N, 103°38′09″E	S.F. Chen & G.Q. Li	MT028621	MT028787	MT028953	MT029094
	AAAAAA	CSF5682	*Eucalyptus* sp.	PingBian County, HongHe Region, YunNan Province, China	23°00′52″N, 103°38′09″E	S.F. Chen & G.Q. Li	MT028622	MT028788	MT028954	MT029095
*N. parvum*	AAAAAA	CSF6220	*E. urophylla* × *E. grandis*	FuNing County, WenShan Region, YunNan Province, China	23°36′26″N, 105°40′43″E	S.F. Chen	MT028623	MT028789	MT028955	MT029096
	AAAAAA	CSF6060^f^	*E. urophylla* × *E. grandis*	JingGu County, PuEr Region, YunNan Province, China	23°23′58″N, 100°50′37″E	S.F. Chen & G.Q. Li	MT028624	MT028790	MT028956	MT029097
	AAAAAA	CSF5818^f^	*Eucalyptus* sp.	MengZi County, HongHe Region, YunNan Province, China	23°12′24″N, 103°30′58″E	S.F. Chen & G.Q. Li	MT028625	MT028791	MT028957	MT029098
	AAABAA	CSF6032^f^	*E. urophylla* × *E. grandis*	NingEr County, PuEr Region, YunNan Province, China	23°05′26″N, 102°02′40″E	S.F. Chen & G.Q. Li	MT028626	MT028792	MT028958	MT029099
	AAABAA	CSF5961^f,h^	*E. urophylla* × *E. grandis*	YuanJiang County, YuXi Region, YunNan Province, China	23°29′04″N, 102°07′34″E	S.F. Chen & G.Q. Li	MT028627	MT028793	MT028959	MT029100
	AAACAA	CSF5664^f^	*Eucalyptus* sp.	PingBian County, HongHe Region, YunNan Province, China	23°00′52″N, 103°38′09″E	S.F. Chen & G.Q. Li	MT028628	MT028794	MT028960	MT029101
	AAA---	CSF6244	*E. urophylla* × *E. grandis*	FuNing County, WenShan Region, YunNan Province, China	23°36′26″N, 105°40′43″E	S.F. Chen	MT028629	MT028795	MT028961	N/A
	AAA---	CSF6067	*E. urophylla* × *E. grandis*	JingGu County, PuEr Region, YunNan Province, China	23°23′58″N, 100°50′37″E	S.F. Chen & G.Q. Li	MT028630	MT028796	MT028962	N/A
	AAA---	CSF6068	*E. urophylla* × *E. grandis*	JingGu County, PuEr Region, YunNan Province, China	23°23′58″N, 100°50′37″E	S.F. Chen & G.Q. Li	MT028631	MT028797	MT028963	N/A
	AAA---	CSF5827	*Eucalyptus* sp.	MengZi County, HongHe Region, YunNan Province, China	23°12′24″N, 103°30′58″E	S.F. Chen & G.Q. Li	MT028632	MT028798	MT028964	N/A
	AAA---	CSF5835	*Eucalyptus* sp.	MengZi County, HongHe Region, YunNan Province, China	23°12′24″N, 103°30′58″E	S.F. Chen & G.Q. Li	MT028633	MT028799	MT028965	N/A
	AAA---	CSF5837	*Eucalyptus* sp.	MengZi County, HongHe Region, YunNan Province, China	23°12′24″N, 103°30′58″E	S.F. Chen & G.Q. Li	MT028634	MT028800	MT028966	N/A
	AAA---	CSF5891	*E. urophylla* × *E. grandis*	PingBian County, HongHe Region, YunNan Province, China	23°09′26″N, 103°32′14″E	S.F. Chen & G.Q. Li	MT028635	MT028801	MT028967	N/A
	AAA---	CSF5897	*E. urophylla* × *E. grandis*	PingBian County, HongHe Region, YunNan Province, China	23°09′26″N, 103°32′14″E	S.F. Chen & G.Q. Li	MT028636	MT028802	MT028968	N/A
	AAA---	CSF5920	*E. urophylla* × *E. grandis*	PingBian County, HongHe Region, YunNan Province, China	23°09′26″N, 103°32′14″E	S.F. Chen & G.Q. Li	MT028637	MT028803	MT028969	N/A
	AAA---	CSF7345	*E. urophylla* × *E. grandis*	PingBian County, HongHe Region, YunNan Province, China	23°08′02″N, 103°32′29″E	S.F. Chen & G.Q. Li	MT028638	MT028804	MT028970	N/A
	AAA---	CSF7348	*E. urophylla* × *E. grandis*	PingBian County, HongHe Region, YunNan Province, China	23°08′02″N, 103°32′29″E	S.F. Chen & G.Q. Li	MT028639	MT028805	MT028971	N/A
	AAA---	CSF5666	*Eucalyptus* sp.	PingBian County, HongHe Region, YunNan Province, China	23°00′52″N, 103°38′09″E	S.F. Chen & G.Q. Li	MT028640	MT028806	MT028972	N/A
	AAA---	CSF5685	*Eucalyptus* sp.	PingBian County, HongHe Region, YunNan Province, China	23°00′52″N, 103°38′09″E	S.F. Chen & G.Q. Li	MT028641	MT028807	MT028973	N/A
	AAA---	CSF5967	*E. urophylla* × *E. grandis*	YuanJiang County, YuXi Region, YunNan Province, China	23°29′04″N, 102°07′34″E	S.F. Chen & G.Q. Li	MT028642	MT028808	MT028974	N/A
	AAA---	CSF5979	*E. urophylla* × *E. grandis*	YuanJiang County, YuXi Region, YunNan Province, China	23°29′04″N, 102°07′34″E	S.F. Chen & G.Q. Li	MT028643	MT028809	MT028975	N/A
	ABAAAA	CSF6219	*E. urophylla* × *E. grandis*	FuNing County, WenShan Region, YunNan Province, China	23°36′26″N, 105°40′43″E	S.F. Chen	MT028644	MT028810	MT028976	MT029102
	ABAAAA	CSF5782^f,h^	*E. globulus*	MengZi County, HongHe Region, YunNan Province, China	23°14′27″N, 103°28′59″E	S.F. Chen & G.Q. Li	MT028645	MT028811	MT028977	MT029103
	ABAAAA	CSF6019^f,h^	*E. urophylla* × *E. grandis*	NingEr County, PuEr Region, YunNan Province, China	23°05′26″N, 102°02′40″E	S.F. Chen & G.Q. Li	MT028646	MT028812	MT028978	MT029104
	ABAAAA	CSF5810	*Eucalyptus* sp.	PingBian County, HongHe Region, YunNan Province, China	23°10′07″N, 103°32′27″E	S.F. Chen & G.Q. Li	MT028647	MT028813	MT028979	MT029105
	ABA---	CSF6252	*E. urophylla* × *E. grandis*	FuNing County, WenShan Region, YunNan Province, China	23°36′26″N, 105°40′43″E	S.F. Chen	MT028648	MT028814	MT028980	N/A
	ABA---	CSF5783	*E. globulus*	MengZi County, HongHe Region, YunNan Province, China	23°14′27″N, 103°28′59″E	S.F. Chen & G.Q. Li	MT028649	MT028815	MT028981	N/A
	ABA---	CSF5784	*E. globulus*	MengZi County, HongHe Region, YunNan Province, China	23°14′27″N, 103°28′59″E	S.F. Chen & G.Q. Li	MT028650	MT028816	MT028982	N/A
	ABA---	CSF5785	*E. globulus*	MengZi County, HongHe Region, YunNan Province, China	23°14′27″N, 103°28′59″E	S.F. Chen & G.Q. Li	MT028651	MT028817	MT028983	N/A
	ABA---	CSF6020	*E. urophylla* × *E. grandis*	NingEr County, PuEr Region, YunNan Province, China	23°05′26″N, 102°02′40″E	S.F. Chen & G.Q. Li	MT028652	MT028818	MT028984	N/A
	ABA---	CSF6031	*E. urophylla* × *E. grandis*	NingEr County, PuEr Region, YunNan Province, China	23°05′26″N, 102°02′40″E	S.F. Chen & G.Q. Li	MT028653	MT028819	MT028985	N/A
	BAAAAA	CSF6224^f^	*E. urophylla* × *E. grandis*	FuNing County, WenShan Region, YunNan Province, China	23°36′26″N, 105°40′43″E	S.F. Chen	MT028654	MT028820	MT028986	MT029106
	BAAAAA	CSF6053	*E. urophylla* × *E. grandis*	JingGu County, PuEr Region, YunNan Province, China	23°20′21″N, 100°54′38″E	S.F. Chen & G.Q. Li	MT028655	MT028821	MT028987	MT029107
	BAAAAA	CSF6038^f,h^	*E. urophylla* × *E. grandis*	NingEr County, PuEr Region, YunNan Province, China	23°05′26″N, 102°02′40″E	S.F. Chen & G.Q. Li	MT028656	MT028822	MT028988	MT029108
	BAADAA	CSF5687^f^	*Eucalyptus* sp.	PingBian County, HongHe Region, YunNan Province, China	23°04′02″N, 103°36′33″E	S.F. Chen & G.Q. Li	MT028657	MT028823	MT028989	MT029109
	BAA---	CSF6230	*E. urophylla* × *E. grandis*	FuNing County, WenShan Region, YunNan Province, China	23°36′26″N, 105°40′43″E	S.F. Chen	MT028658	MT028824	MT028990	N/A
	BAA---	CSF6250	*E. urophylla* × *E. grandis*	FuNing County, WenShan Region, YunNan Province, China	23°36′26″N, 105°40′43″E	S.F. Chen	MT028659	MT028825	MT028991	N/A
	BAA---	CSF6054	*E. urophylla* × *E. grandis*	JingGu County, PuEr Region, YunNan Province, China	23°20′21″N, 100°54′38″E	S.F. Chen & G.Q. Li	MT028660	MT028826	MT028992	N/A
	BAA---	CSF5765^h^	*E. globulus*	MengZi County, HongHe Region, YunNan Province, China	23°14′27″N, 103°28′59″E	S.F. Chen & G.Q. Li	MT028661	MT028827	MT028993	N/A
	BAA---	CSF5824	*Eucalyptus* sp.	MengZi County, HongHe Region, YunNan Province, China	23°12′24″N, 103°30′58″E	S.F. Chen & G.Q. Li	MT028662	MT028828	MT028994	N/A
	BAA---	CSF5753	*Eucalyptus* sp.	PingBian County, HongHe Region, YunNan Province, China	23°08′00″N, 103°32′39″E	S.F. Chen & G.Q. Li	MT028663	MT028829	MT028995	N/A
	BAA---	CSF5798	*Eucalyptus* sp.	PingBian County, HongHe Region, YunNan Province, China	23°10′07″N, 103°32′27″E	S.F. Chen & G.Q. Li	MT028664	MT028830	MT028996	N/A
***N. yunnanense***	AAAAAA	CSF6169	*E. globulus*	AnNing County, KunMing Region, YunNan Province, China	24°55′02″N, 102°23′41″E	S.F. Chen & G.Q. Li	MT028665	MT028831	MT028997	MT029110
	AAAAAA	CSF6171	*E. globulus*	AnNing County, KunMing Region, YunNan Province, China	24°55′02″N, 102°23′41″E	S.F. Chen & G.Q. Li	MT028666	MT028832	MT028998	MT029111
	AAAAAA	CSF6142 = CGMCC3.20083^e,f,g,h^	*E. globulus*	LuFeng County, ChuXiong Region, YunNan Province, China	25°03′12″N, 101°46′29″E	S.F. Chen & G.Q. Li	MT028667	MT028833	MT028999	MT029112
	AAAAAA	CSF6146	*E. globulus*	LuFeng County, ChuXiong Region, YunNan Province, China	25°03′12″N, 101°46′29″E	S.F. Chen & G.Q. Li	MT028668	MT028834	MT029000	MT029113
	AAAAAA	CSF6161	*E. globulus*	LuFeng County, ChuXiong Region, YunNan Province, China	25°03′12″N, 101°46′29″E	S.F. Chen & G.Q. Li	MT028669	MT028835	MT029001	MT029114
	AAAAAA	CSF6166^g^	*E. globulus*	LuFeng County, ChuXiong Region, YunNan Province, China	25°03′12″N, 101°46′29″E	S.F. Chen & G.Q. Li	MT028670	MT028836	MT029002	MT029115
	AAAAAA	CSF7384	*E. globulus*	LuFeng County, ChuXiong Region, YunNan Province, China	25°03′12″N, 101°46′29″E	S.F. Chen & G.Q. Li	MT028671	MT028837	MT029003	MT029116
	AAAAAA	CSF6034 = CGMCC3.20080^f,g,h^	*E. urophylla* × *E. grandis*	NingEr County, PuEr Region, YunNan Province, China	23°05′26″N, 102°02′40″E	S.F. Chen & G.Q. Li	MT028672	MT028838	MT029004	MT029117
	AAAAAA	CSF5686	*Eucalyptus* sp.	PingBian County, HongHe Region, YunNan Province, China	23°04′02″N, 103°36′33″E	S.F. Chen & G.Q. Li	MT028673	MT028839	MT029005	MT029118
	AAA-AA	CSF6036	*E. urophylla* × *E. grandis*	NingEr County, PuEr Region, YunNan Province, China	23°05′26″N, 102°02′40″E	S.F. Chen & G.Q. Li	MT028674	MT028840	MT029006	N/A
	ABAAAA	CSF6175	*E. globulus*	AnNing County, KunMing Region, YunNan Province, China	24°55′02″N, 102°23′41″E	S.F. Chen & G.Q. Li	MT028675	MT028841	MT029007	MT029119
	ABAAAA	CSF6111	*E. globulus*	ChuXiong County, ChuXiong Region, YunNan Province, China	25°02′48″N, 101°41′46″E	S.F. Chen & G.Q. Li	MT028676	MT028842	MT029008	MT029120
	ABAAAA	CSF6225	*E. urophylla* × *E. grandis*	FuNing County, WenShan Region, YunNan Province, China	23°36′26″N, 105°40′43″E	S.F. Chen	MT028677	MT028843	MT029009	MT029121
	ABAAAA	CSF6051	*E. urophylla* × *E. grandis*	JingGu County, PuEr Region, YunNan Province, China	23°20′21″N, 100°54′38″E	S.F. Chen & G.Q. Li	MT028678	MT028844	MT029010	MT029122
	ABAAAA	CSF6137	*E. globulus*	LuFeng County, ChuXiong Region, YunNan Province, China	25°03′12″N, 101°46′29″E	S.F. Chen & G.Q. Li	MT028679	MT028845	MT029011	MT029123
	ABAAAA	CSF5761	*E. globulus*	MengZi County, HongHe Region, YunNan Province, China	23°14′27″N, 103°28′59″E	S.F. Chen & G.Q. Li	MT028680	MT028846	MT029012	MT029124
	ABAAAA	CSF6033	*E. urophylla* × *E. grandis*	NingEr County, PuEr Region, YunNan Province, China	23°05′26″N, 102°02′40″E	S.F. Chen & G.Q. Li	MT028681	MT028847	MT029013	MT029125
	ABAAAA	CSF5706^f,h^	*E. globulus*	PingBian County, HongHe Region, YunNan Province, China	23°05′36″N, 103°31′52″E	S.F. Chen & G.Q. Li	MT028682	MT028848	MT029014	MT029126
	ABAAAA	CSF5974^f,h^	*E. urophylla* × *E. grandis*	YuanJiang County, YuXi Region, YunNan Province, China	23°29′04″N, 102°07′34″E	S.F. Chen & G.Q. Li	MT028683	MT028849	MT029015	MT029127
	ABA---	CSF6184	*E. globulus*	AnNing County, KunMing Region, YunNan Province, China	24°55′02″N, 102°23′41″E	S.F. Chen & G.Q. Li	MT028684	MT028850	MT029016	N/A
	ABA---	CSF6118	*E. globulus*	ChuXiong County, ChuXiong Region, YunNan Province, China	25°02′48″N, 101°41′46″E	S.F. Chen & G.Q. Li	MT028685	MT028851	MT029017	N/A
	ABA---	CSF6122	*E. globulus*	ChuXiong County, ChuXiong Region, YunNan Province, China	25°02′48″N, 101°41′46″E	S.F. Chen & G.Q. Li	MT028686	MT028852	MT029018	N/A
	ABA---	CSF6126	*E. globulus*	ChuXiong County, ChuXiong Region, YunNan Province, China	25°02′48″N, 101°41′46″E	S.F. Chen & G.Q. Li	MT028687	MT028853	MT029019	N/A
	ABA---	CSF6127	*E. globulus*	ChuXiong County, ChuXiong Region, YunNan Province, China	25°02′48″N, 101°41′46″E	S.F. Chen & G.Q. Li	MT028688	MT028854	MT029020	N/A
	ABA---	CSF6247	*E. urophylla* × *E. grandis*	FuNing County, WenShan Region, YunNan Province, China	23°36′26″N, 105°40′43″E	S.F. Chen	MT028689	MT028855	MT029021	N/A
	ABA---	CSF6251	*E. urophylla* × *E. grandis*	FuNing County, WenShan Region, YunNan Province, China	23°36′26″N, 105°40′43″E	S.F. Chen	MT028690	MT028856	MT029022	N/A
	ABA---	CSF6078	*E. urophylla* × *E. grandis*	JingGu County, PuEr Region, YunNan Province, China	23°23′58″N, 100°50′37″E	S.F. Chen & G.Q. Li	MT028691	MT028857	MT029023	N/A
	ABA---	CSF6150	*E. globulus*	LuFeng County, ChuXiong Region, YunNan Province, China	25°03′12″N, 101°46′29″E	S.F. Chen & G.Q. Li	MT028692	MT028858	MT029024	N/A
	ABA---	CSF6152	*E. globulus*	LuFeng County, ChuXiong Region, YunNan Province, China	25°03′12″N, 101°46′29″E	S.F. Chen & G.Q. Li	MT028693	MT028859	MT029025	N/A
	ABA---	CSF6154	*E. globulus*	LuFeng County, ChuXiong Region, YunNan Province, China	25°03′12″N, 101°46′29″E	S.F. Chen & G.Q. Li	MT028694	MT028860	MT029026	N/A
	ABA---	CSF6163	*E. globulus*	LuFeng County, ChuXiong Region, YunNan Province, China	25°03′12″N, 101°46′29″E	S.F. Chen & G.Q. Li	MT028695	MT028861	MT029027	N/A
	ABA---	CSF6165	*E. globulus*	LuFeng County, ChuXiong Region, YunNan Province, China	25°03′12″N, 101°46′29″E	S.F. Chen & G.Q. Li	MT028696	MT028862	MT029028	N/A
	ABA---	CSF7400	*E. globulus*	LuFeng County, ChuXiong Region, YunNan Province, China	25°03′12″N, 101°46′29″E	S.F. Chen & G.Q. Li	MT028697	MT028863	MT029029	N/A
	ABA---	CSF5768	*E. globulus*	MengZi County, HongHe Region, YunNan Province, China	23°14′27″N, 103°28′59″E	S.F. Chen & G.Q. Li	MT028698	MT028864	MT029030	N/A
	ABA---	CSF5778	*E. globulus*	MengZi County, HongHe Region, YunNan Province, China	23°14′27″N, 103°28′59″E	S.F. Chen & G.Q. Li	MT028699	MT028865	MT029031	N/A
	ABA---	CSF5833	*Eucalyptus* sp.	MengZi County, HongHe Region, YunNan Province, China	23°12′24″N, 103°30′58″E	S.F. Chen & G.Q. Li	MT028700	MT028866	MT029032	N/A
	ABA---	CSF5848	*Eucalyptus* sp.	MengZi County, HongHe Region, YunNan Province, China	23°12′24″N, 103°30′58″E	S.F. Chen & G.Q. Li	MT028701	MT028867	MT029033	N/A
	ABA---	CSF5712	*E. globulus*	PingBian County, HongHe Region, YunNan Province, China	23°05′36″N, 103°31′52″E	S.F. Chen & G.Q. Li	MT028702	MT028868	MT029034	N/A
	ABA---	CSF5719	*E. globulus*	PingBian County, HongHe Region, YunNan Province, China	23°05′36″N, 103°31′52″E	S.F. Chen & G.Q. Li	MT028703	MT028869	MT029035	N/A
	ABA---	CSF5739	*Eucalyptus* sp.	PingBian County, HongHe Region, YunNan Province, China	23°08′00″N, 103°32′39″E	S.F. Chen & G.Q. Li	MT028704	MT028870	MT029036	N/A
	ABA---	CSF5751	*Eucalyptus* sp.	PingBian County, HongHe Region, YunNan Province, China	23°08′00″N, 103°32′39″E	S.F. Chen & G.Q. Li	MT028705	MT028871	MT029037	N/A
	ABA---	CSF5873	*E. urophylla* × *E. grandis*	PingBian County, HongHe Region, YunNan Province, China	23°08′02″N, 103°32′29″E	S.F. Chen & G.Q. Li	MT028706	MT028872	MT029038	N/A
	ABA---	CSF5886	*E. urophylla* × *E. grandis*	PingBian County, HongHe Region, YunNan Province, China	23°08′02″N, 103°32′29″E	S.F. Chen & G.Q. Li	MT028707	MT028873	MT029039	N/A
	ABA---	CSF5894	*E. urophylla* × *E. grandis*	PingBian County, HongHe Region, YunNan Province, China	23°09′26″N, 103°32′14″E	S.F. Chen & G.Q. Li	MT028708	MT028874	MT029040	N/A
	ABA---	CSF5900	*E. urophylla* × *E. grandis*	PingBian County, HongHe Region, YunNan Province, China	23°09′26″N, 103°32′14″E	S.F. Chen & G.Q. Li	MT028709	MT028875	MT029041	N/A
	ABA---	CSF5906	*E. urophylla* × *E. grandis*	PingBian County, HongHe Region, YunNan Province, China	23°09′26″N, 103°32′14″E	S.F. Chen & G.Q. Li	MT028710	MT028876	MT029042	N/A
	ABA---	CSF5911	*E. urophylla* × *E. grandis*	PingBian County, HongHe Region, YunNan Province, China	23°09′26″N, 103°32′14″E	S.F. Chen & G.Q. Li	MT028711	MT028877	MT029043	N/A
	ABA---	CSF5918	*E. urophylla* × *E. grandis*	PingBian County, HongHe Region, YunNan Province, China	23°09′26″N, 103°32′14″E	S.F. Chen & G.Q. Li	MT028712	MT028878	MT029044	N/A
	ABA---	CSF7344	*E. urophylla* × *E. grandis*	PingBian County, HongHe Region, YunNan Province, China	23°08′02″N, 103°32′29″E	S.F. Chen & G.Q. Li	MT028713	MT028879	MT029045	N/A
	ABA---	CSF7360	*E. urophylla* × *E. grandis*	PingBian County, HongHe Region, YunNan Province, China	23°08′02″N, 103°32′29″E	S.F. Chen & G.Q. Li	MT028714	MT028880	MT029046	N/A
	ABA---	CSF5788	*Eucalyptus* sp.	PingBian County, HongHe Region, YunNan Province, China	23°10′07″N, 103°32′27″E	S.F. Chen & G.Q. Li	MT028715	MT028881	MT029047	N/A
	ABA---	CSF5793	*Eucalyptus* sp.	PingBian County, HongHe Region, YunNan Province, China	23°10′07″N, 103°32′27″E	S.F. Chen & G.Q. Li	MT028716	MT028882	MT029048	N/A

^a^ Species names in bold are novel species described in this study

^b^ Genotype within each identified species, determined by ITS, *tef1*, *tub2* and *rpb2* loci; ‘-’ means not available

^c^ *CSF* Culture Collection from Southern Forests (CSF), ZhanJiang, GuangDong Province, China, *CGMCC* China General Microbiological Culture Collection Center, Beijing, China

^d^ *ITS* Internal transcribed spacer, *tef1* Translation elongation factor 1-alpha, *tub2* β-tubulin 2, *rpb2* DNA-directed RNA polymerase II subunit, *N/A* Not avaliable

^e^ Isolates represent ex-type

^f^ Isolates used for phylogenetic analyses

^g^ Isolates used for morphological and culture growth studies

^h^ Isolates used for pathogenicity tests

### Phylogenetic analyses

Sequences of the ITS, *tef1* and *tub2* regions for all isolates obtained in this study were generated for species identification. Based on these sequences, the initial genotype of each isolate was determined. Representative isolates based on initial genotype characterisation, host and location for each species were selected for sequencing of the *rpb2* locus. The final genotypes of the selected isolates were thus determined based on sequence data from four loci. Preliminary identification in this study was performed using Standard Nucleotide BLAST (https://blast.ncbi.nlm.nih.gov/Blast.cgi), and available sequences of all species in related genera containing ex-type isolates were downloaded from the NCBI for phylogenetic analyses. The sequences were aligned using the online version of MAFFT v.7 (http://mafft.cbrc.jp/alignment/server/) (Katoh and Standley 2013), with the iterative refinement method (FFT-NS-i setting). The alignments were checked manually and edited in MEGA v.6.0.5 (Tamura et al. 2013). Sequence alignments were deposited in TreeBASE.

Maximum likelihood (ML) analyses with 1000 bootstrap replicates were conducted using PhyML v.3.0 (Guindon et al. 2010). The best-fit model of nucleotide substitution for each dataset was determined using jModelTest v.2.1.5 (Darriba et al. 2012). Maximum parsimony (MP) trees were generated in PAUP v.1.0b10 (Swofford 2002), using the heuristic search function with tree bisection and reconstruction (TBR) as branch swapping algorithms and 1000 random addition replicates. Gaps were treated as a fifth character and the characters were unordered and given equal weight. MAXTREES were set to 5000, branches of zero length were collapsed and all multiple, equally parsimonious trees were saved. Tree length (TL), consistency index (CI), retention index (RI), rescaled consistency index (RC) and homoplasy index (HI) were calculated. Bootstrap support values were evaluated using 1000 bootstrap replicates (Hillis and Bull 1993). The phylogenetic analyses for *Botryosphaeria* were rooted using *N. parvum* (ATCC 58191), and phylogenetic analyses for *Lasiodiplodia* and *Neofusicoccum* were rooted using *Botryosphaeria dothidea* (CBS 115476) (Table 2).

**Table 2:** Isolates from other studies and used in the phylogenetic analyses for this study

Species	Isolate No.^a^	Host	Location	Collector	GenBank accession No.^b^				Reference
					ITS	*tef1*	*tub2*	*rpb2*	
*Botryosphaeria agaves*	MFLUCC 11–0125	*Agave* sp.	Thailand	R. Phookamsak	JX646791	JX646856	JX646841	N/A	Liu et al. 2012
	= CBS 133992^c^								
	MFLUCC 10–0051	*Agave* sp.	Thailand	P. Chomnunti	JX646790	JX646855	JX646840	N/A	Liu et al. 2012
*B. auasmontanum*	CMW 25413	*Acacia mellifera*	Namibia	F.J.J. van der Walt & J. Roux	EU101303	EU101348	N/A	N/A	Slippers et al. 2014
	= CBS 121769^c^								
*B. corticis*	CBS 119047^c^	*Vaccinium corymbosum*	USA	P.V. Oudemans	DQ299245	EU017539	EU673107	N/A	Phillips et al. 2006, 2008
	ATCC 22927	*Vaccinium* sp.	USA	R.D. Millholland	DQ299247	EU673291	EU673108	N/A	Phillips et al. 2006, 2008
*B. dothidea*	CBS 115476	*Prunus* sp.	Switzerland	B. Slippers	AY236949	AY236898	AY236927	EU339577	Slippers et al. 2004a, Phillips et al. 2008
	= CMW 8000^c^								
	CBS 110302	*Vitis vinifera*	Portugal	A.J.L. Phillips	AY259092	AY573218	EU673106	N/A	Alves et al. 2004, Phillips et al. 2008
*B. fabicerciana*	CMW 27094	*Eucalyptus* sp.	China	M.J. Wingfield	HQ332197	HQ332213	KF779068	MF410137	Chen et al. 2011; Li et al. 2018
	= CBS 127193^c^								
	CMW 27121	*Eucalyptus* sp.	China	M.J. Wingfield	HQ332198	HQ332214	KF779069	MF410138	Chen et al. 2011, Li et al. 2018
	= CBS 127194								
*B. fusispora*	MFLUCC 10–0098^c^	*Entada* sp.	Thailand	S. Boonmee	JX646789	JX646854	JX646839	N/A	Liu et al. 2012
	MFLUCC 11–0507	*Entada* sp.	Thailand	R. Cheewangkoon	JX646788	JX646853	JX646838	N/A	Liu et al. 2012
*B. kuwatsukai*	CBS 135219	*Malus domestica*	China	C.S. Wang	KJ433388	KJ433410	N/A	N/A	Xu et al. 2015
	= PG 2^c^								
	LSP 5	*Pyrus* sp.	China	C.S. Wang	KJ433395	KJ433417	N/A	N/A	Xu et al. 2015
*B. minutispermatia*	GZCC 16–0013^c^	dead wood	Guizhou, China	H.A. Ariyawansa	KX447675	KX447678	N/A	N/A	Ariyawansa et al. 2016
	GZCC 16–0014	dead wood	Guizhou, China	H.A. Ariyawansa	KX447676	KX447679	N/A	N/A	Ariyawansa et al. 2016
*B. pseudoramosa*	CERC2001 = CGMCC3.18739^c^	*Eucalyptus* hybrid	GuangXi, China	S.F. Chen & G.Q. Li	KX277989	KX278094	KX278198	MF410140	Li et al. 2018
	CERC2983	*Melastoma sanguineum*	ZhanJiang Region, GuangDong Province, China	S.F. Chen	KX277992	KX278097	KX278201	MF410143	Li et al. 2018
	=CGMCC3.18740								
*B. qingyuanensis*	CERC2946	*Eucalyptus* hybrid	QingYuan Region, GuangDong Province, China	S.F. Chen & G.Q. Li	KX278000	KX278105	KX278209	MF410151	Li et al. 2018
	= CGMCC3.18742^c^								
	CERC2947	*Eucalyptus* hybrid	QingYuan Region, GuangDong Province, China	S.F. Chen & G.Q. Li	KX278001	KX278106	KX278210	MF410152	Li et al. 2018
	= CGMCC3.18743								
*B. ramosa*	CBS 122069	*Eucalyptus camaldulensis*	Australia	T.I. Burgess	EU144055	EU144070	KF766132	N/A	Pavlic et al. 2008, Slippers et al. 2013
	= CMW 26167^c^								
*B. rosaceae*	CGMCC3.18007^c^	*Malus* sp.	Shandong, China	Y. Zhang & J.Q. Zhang	KX197074	KX197094	KX197101	N/A	Zhou et al. 2017
	CGMCC3.18008	*Amygdalus* sp.	Shandong, China	Y. Zhang, J.Q. Zhang & Z.P. Dou	KX197075	KX197095	KX197102	N/A	Zhou et al. 2017
*B. scharifii*	IRAN 1529C	*Mangifera indica*	Iran	J. Abdollahzadeh	JQ772020	JQ772057	N/A	N/A	Abdollahzadeh et al. 2013
	= CBS 124703^c^								
	IRAN 1543C	*Mangifera indica*	Iran	J. Abdollahzadeh & A. Javadi	JQ772019	JQ772056	N/A	N/A	Abdollahzadeh et al. 2013
	= CBS 124702								
*B. sinensia*	CGMCC3.17723	*Morus* sp.	Henan, China	Z.P. Dou	KT343254	KU221233	KX197107	N/A	Zhou et al. 2016, 2017
	CGMCC3.17724	*Juglans regia*	Henan, China	Z.P. Dou	KT343256	KU221234	KX197108	N/A	Zhou et al. 2016, 2017
*B. wangensis*	CERC2298	*Cedrus deodara*	RuZhou Region, HeNan Province, China	S.F. Chen	KX278002	KX278107	KX278211	MF410153	Li et al. 2018
	= CGMCC3.18744^c^								
	CERC2299	*Cedrus deodara*	RuZhou Region, HeNan Province, China	S.F. Chen	KX278003	KX278108	KX278212	MF410154	Li et al. 2018
	= CGMCC3.18745								
*Lasiodiplodia avicenniae*	CMW 41467^c^	*Avicennia marina*	South Africa	J.A. Osorio & J. Roux	KP860835	KP860680	KP860758	KU587878	Osorio et al. 2017
	LAS 199 DNA	*Avicennia marina*	South Africa	J.A. Osorio & J. Roux	KU587957	KU587947	KU587868	KU587880	Osorio et al. 2017
*L. americana*	CERC1961	*Pistachia vera*	Arizona, USA	T.J. Michailides	KP217059	KP217067	KP217075	MF410161	Chen et al. 2015, Li et al. 2018
	= CFCC50065^c^								
	CERC1960	*Pistachia vera*	Arizona, USA	T.J. Michailides	KP217058	KP217066	KP217074	MF410162	Chen et al. 2015, Li et al. 2018
	= CFCC50064								
*L. brasiliense*	CMM 4015^c^	*Mangifera indica*	Brazil	M.W. Marques	JX464063	JX464049	N/A	N/A	Netto et al. 2014
	CMW 35884	*Adansonia madagascariensis*	Madagascar		KU887094	KU886972	KU887466	KU696345	Cruywagen et al. 2017
*L. bruguierae*	CMW 41470^c^	*Bruguiera gymnorrhiza*	South Africa	J.A. Osorio & J. Roux	KP860833	KP860678	KP860756	KU587875	Osorio et al. 2017
	CMW 41614	*Bruguiera gymnorrhiza*	South Africa	J.A. Osorio & J. Roux	KP860834	KP860679	KP860757	KU587877	Osorio et al. 2017
*L. caatinguensis*	CMM 1325^c^	*Citrus sinensis*	Itarema, Ceará, Brazil	I.B.L. Coutinho & J.S. Lima	KT154760	KT008006	KT154767	N/A	Coutinho et al. 2017
	IBL 40	*Spondias mombin*	Itarema, Ceará, Brazil	J.S. Lima & J.E. Cardoso	KT154762	KT154755	KT154769	N/A	Coutinho et al. 2017
*L. chinensis*	CGMCC3.18061^c^	Unknown	China	W. He & Z.P. Dou	KX499889	KX499927	KX500002	KX499965	Dou et al. 2017a
	CGMCC3.18066	*Hevea brasiliensis*	China	Y. Zhang & Y.P. Zhou	KX499899	KX499937	KX500012	KX499974	Dou et al. 2017a
*L. chonburiensis*	MFLUCC 16–0376 = KUMCC 17–0299 ^c^	*Pandanus* sp.	Thailand	W. Jaidee	MH275066	MH412773	MH412742	N/A	Tibpromma et al. 2018
*L. cinnamomi*	CFCC 51997 ^c^	*Cinnamomum camphora*	China	N. Jiang	MG866028	MH236799	MH236797	MH236801	Jiang et al. 2018
	CFCC 51998	*Cinnamomum camphora*	China	N. Jiang	MG866029	MH236800	MH236798	MH236802	Jiang et al. 2018
*L. citricola*	CBS 124707	*Citrus* sp.	Iran	J. Abdollahzadeh & A. Javadi	GU945354	GU945340	KU887505	KU696351	Abdollahzadeh et al. 2010, Cruywagen et al. 2017
	= IRAN 1522C^c^								
	CBS 124706	*Citrus* sp.	Iran	A. Shekari	GU945353	GU945339	KU887504	KU696350	Abdollahzadeh et al. 2010, Cruywagen et al. 2017
	= IRAN 1521C								
*L. crassispora*	CBS 118741	*Santalum album*	Kununurra, Australia	T.I. Burgess & B. Dell	DQ103550	EU673303	KU887506	KU696353	Burgess et al. 2006b, Phillips et al. 2008, Cruywagen et al. 2017
	= WAC12533^c^								
	CBS 110492	Unknown	Unknown	Unknown	EF622086	EF622066	EU673134	N/A	Alves et al. 2008, Phillips et al. 2008
*L. euphorbicola*	CMM 3609^c^	*Jatropha curcas*	Brazil	A. R. Machado & O. L. Pereira	KF234543	KF226689	KF254926	N/A	Machado et al. 2014
	CMW 33350	*Adansonia digitata*	Botswana		KU887149	KU887026	KU887455	KU696346	Cruywagen et al. 2017
*L. exigua*	CBS 137785^c^	*Retama raetam*	Tunisia	B.T. Linaldeddu	KJ638317	KJ638336	KU887509	KU696355	Linaldeddu et al. 2015, Cruywagen et al. 2017
	BL 184	*Retama raetam*	Tunisia	B.T. Linaldeddu	KJ638318	KJ638337	N/A	N/A	Linaldeddu et al. 2015
*L. gilanensis*	CBS 124704	Unknown	Iran	J. Abdollahzadeh & A. Javadi	GU945351	GU945342	KU887511	KU696357	Abdollahzadeh et al. 2010, Cruywagen et al. 2017
	= IRAN 1523C^c^								
	CBS 124705	Unknown	Iran	J. Abdollahzadeh & A. Javadi	GU945352	GU945341	KU887510	KU696356	Abdollahzadeh et al. 2010, Cruywagen et al. 2017
	= IRAN 1501C								
*L. gonubiensis*	CBS 115812	*Syzygium cordatum*	South Africa	D. Pavlic	AY639595	DQ103566	DQ458860	KU696359	Pavlic et al. 2004, Burgess et al. 2006b, Phillips et al. 2008, Cruywagen et al. 2017
	= CMW 14077^c^								
	CBS 116355	*Syzygium cordatum*	South Africa	D. Pavlic	AY639594	DQ103567	EU673126	KU696358	Pavlic et al. 2004, Burgess et al. 2006b, Phillips et al. 2008, Cruywagen et al. 2017
	= CMW 14078								
*L. gravistriata*	CMM 4564^c^	*Anacardium humile*	Brazil	M.S.B. Netto	KT250949	KT250950	N/A	N/A	Netto et al. 2017
	CMM 4565	*Anacardium humile*	Brazil	M.S.B. Netto	KT250947	KT266812	N/A	N/A	Netto et al. 2017
*L. hormozganensis*	CBS 124709	*Olea* sp.	Iran	J. Abdollahzadeh & A. Javadi	GU945355	GU945343	KU887515	KU696361	Abdollahzadeh et al. 2010, Cruywagen et al. 2017
	= IRAN 1500C^c^								
	CBS 124708	*Mangifera indica*	Iran	J. Abdollahzadeh & A. Javadi	GU945356	GU945344	KU887514	KU696360	Abdollahzadeh et al. 2010, Cruywagen et al. 2017
	= IRAN 1498C								
*L. hyalina*	CGMCC3.17975^c^	*Acacia confusa*	China	Y. Zhang & Y. P. Zhou	KX499879	KX499917	KX499992	KX499955	Dou et al. 2017b
	CGMCC3.18383	unknown tree	China	Z. P. Dou & Z. C. Liu	KY767661	KY751302	KY751299	KY751296	Dou et al. 2017b
	= B 6180								
*L. indica*	IBP 01^c^	*Angiospermous* tree	India	I.B. Prasher & G. Singh	KM376151	N/A	N/A	N/A	Prasher and Singh 2014
*L. iraniensis*	IRAN 1520C^c^	*Salvadora persica*	Iran	J. Abdollahzadeh & A. Javadi	GU945348	GU945336	KU887516	KU696363	Abdollahzadeh et al. 2010, Cruywagen et al. 2017
	IRAN 1502C	*Juglans* sp.	Iran	A. Javadi	GU945347	GU945335	KU887517	KU696362	Abdollahzadeh et al. 2010, Cruywagen et al. 2017
*L. laeliocattleyae*	CBS 167.28^c^	*Laeliocattleya*	Italy	C. Sibilia	KU507487	KU507454	N/A	N/A	Rodríguez-Gálvez et al. 2017
	LAREP 1	*Mangifera indica*	Repartidor, Peru	P. Guerrero	KU507484	KU507451	N/A	N/A	Rodríguez-Gálvez et al. 2017
*L. lignicola*	MFLUCC 11–0435	Unknown	Thailand	A.D. Ariyawansa	JX646797	KU887003	JX646845	KU696364	Liu et al. 2012, Cruywagen et al. 2017
	= CBS 134112^c^								
*L. macrospora*	CMM 3833^c^	*Jatropha curcas*	Brazil	A.R. Machado & O.L. Pereira	KF234557	KF226718	KF254941	N/A	Machado et al. 2014
*L. mahajangana*	CBS 124925	*Terminalia catappa*	Madagascar	J. Roux	FJ900595	FJ900641	FJ900630	KU696365	Begoude et al. 2010, Cruywagen et al. 2017
	= CMW 27801^c^								
	CBS 124926	*Terminalia catappa*	Madagascar	J. Roux	FJ900596	FJ900642	KU887519	KU696366	Begoude et al. 2010, Cruywagen et al. 2017
	= CMW 27820								
*L. margaritacea*	CBS 122519	*Adansonia gibbosa*	WA, Tunnel Creek Gorge	T.I. Burgess	EU144050	EU144065	KU887520	KU696367	Pavlic et al. 2008, Cruywagen et al. 2017
	= CMW 26162^c^								
*L. mediterranea*	CBS 137783^c^	*Quercus ilex*	Italy	B.T. Linaldeddu	KJ638312	KJ638331	KU887521	KU696368	Linaldeddu et al. 2015
	CBS 137784	*Vitis vinifera*	Italy	*S. Serra*	KJ638311	KJ638330	KU887522	KU696369	Linaldeddu et al. 2015
*L. missouriana*	CBS 128311	*Vitis* sp. × *Vitis labruscana*	Missouri, USA	K. Striegler & G.M. Leavitt	HQ288225	HQ288267	HQ288304	KU696370	Urbez-Torres et al. 2012, Cruywagen et al. 2017
	= UCD2193MO^c^								
	CBS 128312	*Vitis* sp. × *Vitis labruscana*	Missouri, USA	K. Striegler & G.M. Leavitt	HQ288226	HQ288268	HQ288305	KU696371	Urbez-Torres et al. 2012, Cruywagen et al. 2017
	= UCD2199MO								
*L. pandanicola*	MFLUCC 16–0265 = KUMCC 16–0158^c^	*Pandanus* sp.	Thailand	B. Thongbai	MH275068	MH412774	N/A	N/A	Tibpromma et al. 2018
*L. parva*	CBS 456.78^c^	Cassava-field soil	Colombia	O. Rangel	EF622083	EF622063	KU887523	KU696372	Alves et al. 2008, Cruywagen et al. 2017
	CBS 494.78	Cassava-field soil	Colombia	O. Rangel	EF622084	EF622064	EU673114	KU696373	Alves et al. 2008, Phillips et al. 2008, Cruywagen et al. 2017
*L. plurivora*	CBS 120832^c^	*Prunus salicina*	Stellenbosch, Western Cape, South Africa	U. Damm	EF445362	EF445395	KU887524	KU696374	Damm et al. 2007, Cruywagen et al. 2017
	CBS 121103	*Vitis vinifera*	South Africa	F. Halleen	AY343482	EF445396	KU887525	KU696375	Damm et al. 2007, Cruywagen et al. 2017
*L. pontae*	CMM 1277^c^	*Spondias purpurea*	Pio-IX/Piauí/Brazil	J.S. Lima & F.C.O. Freire	KT151794	KT151791	KT151797	N/A	Coutinho et al. 2017
*L. pseudotheobromae*	CBS 116459^c^	*Gmelina arborea*	Costa Rica	J. Carranza & Velásquez	EF622077	EF622057	EU673111	KU696376	Alves et al. 2008, Phillips et al. 2008, Cruywagen et al. 2017
	CMM 3887	*Jatropha curcas*	Brazil	A. R. Machado	KF234559	KF226722	KF254943	N/A	Machado et al. 2014
*L. pyriformis*	CBS 121770	*Acacia mellifera*	Dordabis, Namibia	F.J.J. van der Walt & J. Roux	EU101307	EU101352	KU887527	KU696378	Slippers et al. 2014, Cruywagen et al. 2017
	= CMW 25414^c^								
	CBS 121771	*Acacia mellifera*	Dordabis, Namibia	F.J.J. van der Walt & J. Roux	EU101308	EU101353	KU887528	KU696379	Slippers et al. 2014, Cruywagen et al. 2017
	= CMW 25415								
*L. rubropurpurea*	CBS 118740	*Eucalyptus grandis*	Tully, Queensland	T.I. Burgess & G. Pegg	DQ103553	DQ103571	EU673136	KU696380	Burgess et al. 2006b, Phillips et al. 2008, Cruywagen et al. 2017
	= CMW 14700								
	= WAC 12535^c^								
	WAC 12536	*Eucalyptus grandis*	Tully, Queensland	T.I. Burgess & G. Pegg	DQ103554	DQ103572	KU887530	KU696381	Burgess et al. 2006b, Cruywagen et al. 2017
	= CMW 15207								
*L. sterculiae*	CBS 342.78^c^	*Sterculia oblonga*	Germany	S. Bruhn	KX464140	KX464634	KX464908	KX463989	Yang et al. 2017
*L. subglobosa*	CMM 3872^c^	*Jatropha curcas*	Brazil	A.R. Machado & O.L. Pereira	KF234558	KF226721	KF254942	N/A	Machado et al. 2014
	CMM 4046	*Jatropha curcas*	Brazil	A.R. Machado & O.L. Pereira	KF234560	KF226723	KF254944	N/A	Machado et al. 2014
*L. thailandica*	CPC 22795^c^	*Mangifera indica*	Thailand	T. Trakunyingcharoen	KJ193637	KJ193681	N/A	N/A	Trakunyingcharoen et al. 2015
	CPC 22755	*Phyllanthus acidus*	Thailand	T. Trakunyingcharoen	KM006433	KM006464	N/A	N/A	Trakunyingcharoen et al. 2015
*L. theobromae*	CBS 164.96^c^	Fruit along coral reef coast	New Guinea	A. Aptroot	AY640255	AY640258	KU887532	KU696383	Phillips et al. 2005, Cruywagen et al. 2017
	CBS 111530	Unknown	Unknown	Unknown	EF622074	EF622054	KU887531	KU696382	Alves et al. 2008, Cruywagen et al. 2017
*L. venezuelensis*	CBS 118739	*Acacia mangium*	Acarigua, Venezuela	S. Mohali	DQ103547	DQ103568	KU887533	KU696384	Burgess et al. 2006b, Cruywagen et al. 2017
	= CMW 13511								
	= WAC 12539^c^								
	CMW 13512	*Acacia mangium*	Acarigua, Venezuela	S. Mohali	DQ103548	DQ103569	KU887534	N/A	Burgess et al. 2006b, Cruywagen et al. 2017
	= WAC 12540								
*L. viticola*	CBS 128313	*Vitis vinifera*	USA	K. Striegler & G.M. Leavitt	HQ288227	HQ288269	HQ288306	KU696385	Urbez-Torres et al. 2012, Cruywagen et al. 2017
	= UCD 2553AR^c^								
	CBS 128315	*Vitis vinifera*	USA	K. Striegler & G.M. Leavitt	HQ288228	HQ288270	HQ288307	KU696386	Urbez-Torres et al. 2012, Cruywagen et al. 2017
	= UCD 2604MO								
*L. vitis*	CBS 124060^c^	*Vitis vinifera*	Italy	S. Burruano	KX464148	KX464642	KX464917	KX463994	Yang et al. 2017
*Neofusicoccum algeriense*	CBS 137504	*Vitis vinifera*	Algeria	A. Berraf-Tebbal	KJ657702	KJ657715	KX505915	N/A	Berraf-Tebbal et al. 2014, Lopes et al. 2017
	= ALG 1^c^								
	CAA 322	*Malus domestica*	Portugal		KX505906	KX505894	KX505916	N/A	Lopes et al. 2017
*N. andinum*	CBS 117453	*Eucalyptus* sp.	Me´ rida state, Venezuela	S. Mohali	AY693976	AY693977	KX464923	KX464002	Mohali et al. 2006, Yang et al. 2017
	= CMW13455^c^								
	CBS 117452	*Eucalyptus* sp.	Me´ rida state, Venezuela	S. Mohali	DQ306263	DQ306264	KX464922	KX464001	Mohali et al. 2006, Yang et al. 2017
	= CMW 13446								
*N. arbuti*	CBS 116131^c^	*Arbutus menziesii*	Washington, USA	M. Elliott	AY819720	KF531792	KF531793	KX464003	Farr et al. 2005, Phillips et al. 2013, Yang et al. 2017
	CBS 117090	*Arbutus menziesii*	California, USA	M. Elliott	AY819724	KF531791	KF531794	N/A	Farr et al. 2005, Phillips et al. 2013
*N. australe*	CMW 6837^c^	*Acacia sp.*	Batemans Bay, Australia	M.J. Wingfield	AY339262	AY339270	AY339254	EU339573	Slippers et al. 2004c, Yang et al. 2017
	CBS 110865	*Vitis vinifera*	South Africa	F. Halleen	AY343408	KX464661	KX464937	KX464005	van Niekerk et al. 2004, Yang et al. 2017
	= CPC 4599								
*N. batangarum*	CBS 124924	*Terminalia catappa*	Cameroon	D. Begoude & J. Roux	FJ900607	FJ900653	FJ900634	FJ900615	Begoude 2010
	= CMW 28363^c^								
	CBS 124923	*Terminalia catappa*	Cameroon	D. Begoude & J. Roux	FJ900608	FJ900654	FJ900635	FJ900616	Begoude 2010
	= CMW 28320								
*N. brasiliense*	CMM 1338^c^	*Mangifera indica*	Brazil	M.W. Marques	JX513630	JX513610	KC794031	N/A	Marques et al. 2013
	CMM 1285	*Mangifera indica*	Brazil	M.W. Marques	JX513628	JX513608	KC794030	N/A	Marques et al. 2013
*N. buxi*	CBS 116.75^c^	*Buxus sempervirens*	France	H.A. van der Aa	KX464165	KX464678	N/A	KX464010	Yang et al. 2017
	CBS 113714	*Buxus sempervirens*	Sweden	O. Constantinescu	KX464164	KX464677	KX464954	KX464009	Yang et al. 2017
*N. cordaticola*	CBS 123634	*Syzigium cordatum*	South Africa	D. Pavlic	EU821898	EU821868	EU821838	EU821928	Pavlic et al. 2009a, 2009b, Yang et al. 2017
	= CMW 13992^c^								
	CBS 123635	*Syzigium cordatum*	South Africa	D. Pavlic	EU821903	EU821873	EU821843	EU821933	Pavlic et al. 2009a, 2009b
	= CMW 14056								
*N. corticosae*	CBS 120081^c^	*Eucalyptus corticosa*	Australia	B.A. Summerell	DQ923533	KX464682	KX464958	KX464013	Summerell et al. 2006, Yang et al. 2017
	CBS 118099	*Eucalyptus camaldulensis*	Australia	P. Barber	KX464168	KX464681	KX464957	KX464012	Yang et al. 2017
*N. cryptoaustrale*	CMW 23785	*Eucalyptus* trees	South Africa	H.M. Maleme	FJ752742	FJ752713	FJ752756	KX464014	Crous et al. 2013, Yang et al. 2017
	= CBS 122813^c^								
*N. eucalypticola*	CBS 115679	*Eucalyptus grandis*	Orbost, Victoria, Australia	M.J. Wingfield	AY615141	AY615133	AY615125	N/A	Slippers et al. 2004b
	= CMW 6539^c^								
	CBS 115766	*Eucalyptus rossii*	Tidbinbilla, NSW, Australia	M.J. Wingfield	AY615143	AY615135	AY615127	N/A	Slippers et al. 2004b, 2013
	= CMW 6217								
*N. eucalyptorum*	CBS 115791	*Eucaluptus grandis*	South Africa	H. Smith	AF283686	AY236891	AY236920	N/A	Smith et al. 2001, Slippers et al. 2004c
	= CMW10125^c^								
	CMW 10126	*Eucaluptus grandis*	South Africa	H. Smith	AF283687	AY236892	AY236921	N/A	Smith et al. 2001, Slippers et al. 2004c
*N. grevilleae*	CBS 129518	*Grevillea aurea*	Australia	P.W. Crous & R.G. Shivas	JF951137	N/A	N/A	N/A	Crous et al. 2011
	= CPC 16999^c^								
*N. hellenicum*	CERC1947	*Pistachia vera*	Thessaloniki, Greece	T.J. Michailides	KP217053	KP217061	KP217069	N/A	Chen et al. 2015
	= CFCC50067^c^								
	CERC1948	*Pistachia vera*	Aghios Mamas, Chalkidiki, Greece	T.J. Michailides	KP217054	KP217062	KP217070	N/A	Chen et al. 2015
	= CFCC50068								
*N. hongkongense*	CERC2968	*A. cunninghamii*	HongKong, China	S.F. Chen	KX278051	KX278156	KX278260	KX278282	Li et al. 2018
	= CGMCC3.18748								
	CERC2973	*A. cunninghamii*	HongKong, China	S.F. Chen	KX278052	KX278157	KX278261	KX278283	Li et al. 2018
	= CGMCC3.18749^c^								
*N. illicii*	CGMCC3.18310^c^	*Illicium verum*	Guangxi, China	L. Wang	KY350149	N/A	KY350155	N/A	Zhang et al. 2017
	CGMCC3.18311	*Illicium verum*	Guangxi, China	L. Wang	KY350150	KY817756	KY350156	N/A	Zhang et al. 2017
*N. italicum*	MFLUCC 15–0900^c^	*Vitis vinifera*	Italy	E. Camporesi	KY856755	KY856754	N/A	N/A	Marin-Felix et al. 2017
*N. kwambonambiense*	CBS 123639	*Syzigium cordatum*	South Africa	D. Pavlic	EU821900	EU821870	EU821840	EU821930	Pavlic et al. 2009a, 2009b
	= CMW 14023^c^								
	CBS 123641	*Syzigium cordatum*	South Africa	D. Pavlic	EU821919	EU821889	EU821859	EU821949	Pavlic et al. 2009a, 2009b
	= CMW 14140								
*N. lumnitzerae*	CMW 41469^c^	*Lumnitzera racemosa*	South Africa	J.A. Osorio & J. Roux	KP860881	KP860724	KP860801	KU587925	Osorio et al. 2017
	CMW 41228	*Lumnitzera racemosa*	South Africa	J.A. Osorio & J. Roux	KP860882	KP860725	KP860803	KU587926	Osorio et al. 2017
*N. luteum*	CBS 562.92	*Actinidia deliciosa, lesion on ripe fruit*	New Zealand	S.R. Pennycook	KX464170	KX464690	KX464968	KX464020	Yang et al. 2017
	= ATCC 58193^c^								
*N. macroclavatum*	CBS 118223	*Eucalyptus globulus*	Western Australia	T. Burgess	DQ093196	DQ093217	DQ093206	KX464022	Burgess et al. 2005, Yang et al. 2017
	= WAC 12444^c^								
*N. mangiferae*	CBS 118531	*Mangifera indica*	Australia	G.I. Johnson	AY615185	DQ093221	AY615172	N/A	Burgess et al. 2005, Slippers et al. 2005
	= CMW 7024								
	CBS 118532	*Mangifera indica*	Australia	G.I. Johnson	AY615186	DQ093220	AY615173	KX464023	Burgess et al. 2005, Slippers et al. 2005, Yang et al. 2017
	= CMW 7797								
*N. mangroviorum*	CMW 41365^c^	*Avicennia marina*	South Africa	J.A. Osorio & J. Roux	KP860859	KP860702	KP860779	KU587905	Osorio et al. 2017
	CMW 42481	*Bruguiera gymnorrhiza*	South Africa	J.A. Osorio & J. Roux	KP860848	KP860692	KP860770	KU587895	Osorio et al. 2017
*N. mediterraneum*	CBS 121718	*Eucalyptus* sp.	Greece	P.W. Crous, M.J. Wingfield & A.J.L. Phillips	GU251176	GU251308	GU251836	KX464024	Crous et al. 2007, Yang et al. 2017
	= CPC 13137^c^								
*N. microconidium*	CERC3497	*E. urophylla* × *E. grandis*	ZhanJiang Region, GuangDong Province, China	S.F. Chen & G.Q. Li	KX278053	KX278158	KX278262	MF410203	Li et al. 2018
	= CGMCC3.18750^c^								
	CERC3498	*E. urophylla* × *E. grandis*	ZhanJiang Region, GuangDong Province, China	S.F. Chen & G.Q. Li	KX278054	KX278159	KX278263	MF410204	Li et al. 2018
	= CGMCC3.18751								
*N. nonquaesitum*	CBS 126655	*Umbellularia californica*	USA	F.P. Trouillas	GU251163	GU251295	GU251823	KX464025	Inderbitzin et al. 2010, Yang et al. 2017
	= PD 484^c^								
	PD 301	*Vaccinum corymbosum* cv. Elliot	Chile	E.X. Bricen˜o, J.G. Espinoza, B.A. Latorre & J.G. Espinoza	GU251164	GU251296	GU251824	N/A	Inderbitzin et al. 2010
*N. occulatum*	CBS 128008	*Eucalyptus grandis* hybrid	Australia	T.I. Burgess	EU301030	EU339509	EU339472	EU339558	Sakalidis et al. 2011
	= MUCC 227^c^								
	MUCC 286	*Eucalyptus pellita*	Australia	T.I. Burgess	EU736947	EU339511	EU339474	EU339560	Sakalidis et al. 2011
	= WAC 12395								
*N. pandanicola*	MFLUCC 17–2270 = KUMCC 17–0184 ^c^	*Pandanus* sp.	China	T. Aluthwaththa	MH275072	N/A	N/A	N/A	Tibpromma et al. 2018
*N. parvum*	ATCC 58191	*Populus nigra*	New Zealand	G.J. Samuels	AY236943	AY236888	AY236917	EU821963	Slippers et al. 2004a, Pavlic et al. 2009a
	= CMW 9081^c^								
	CMW 9080	*Populus nigra*	New Zealand	G.J. Samuels	AY236942	AY236887	AY236916	EU821962	Slippers et al. 2004a, Pavlic et al. 2009a
	= ICMP 8002								
*N. pennatisporum*	WAC 13153	*Allocasuarina fraseriana*	Western Australia	K.M. Taylor	EF591925	EF591976	EF591959	N/A	Taylor et al. 2009
	= MUCC 510^c^								
*N. pistaciae*	CBS 595.76^c^	*Pistacia vera*	Greece	D.G. Zachos	KX464163	KX464676	KX464953	KX464008	Yang et al. 2017
*N. pistaciarum*	CBS 113083	*Pistacia vera*	USA	T.J. Michailides	KX464186	KX464712	KX464998	KX464027	Yang et al. 2017
	= CPC 5263^c^								
	CBS 113084	Redwood	USA	T.J. Michailides	KX464187	KX464713	KX464999	KX464028	Yang et al. 2017
	= CPC 5284								
*N. pistaciicola*	CBS 113089^c^	*Pistacia vera*	USA	T.J. Michailides	KX464199	KX464727	KX465014	KX464033	Marin-Felix et al. 2017, Yang et al. 2017
*N. protearum*	CBS 114176	*Leucadendron salignum*	South Africa	S. Denman	AF452539	KX464720	KX465006	KX464029	Denman et al. 2003, Yang et al. 2017
	= STE-U 1775^c^								
	CBS 111200	*Leucadendron* sp.	South Africa	P.W. Crous	KX464193	KX464719	KX465005	N/A	Yang et al. 2017
	= CPC 1357								
*N. pruni*	CBS 121112^c^	*Prunus salicina*	South Africa	U. Damm	EF445349	EF445391	KX465016	KX464034	Damm et al. 2007, Marin-Felix et al. 2017, Yang et al. 2017
*N. ribis*	CBS 115475	*Ribes* sp.	USA	B. Slippers & G. Hudler	AY236935	AY236877	AY236906	EU821958	Slippers et al. 2004a, Pavlic et al. 2009a
	= CMW 7772^c^								
	CBS 121.26	*Ribes rubrum*	USA	N.E. Stevens	AF241177	AY236879	AY236908	EU821960	Slippers et al. 2004a, Pavlic et al. 2009a
	= CMW 7054								
	= CPC4598^c^								
*N. sinense*	CGMCC3.18315^c^	*unknown woody plant*	Guizhou, China	J.J. Gan	KY350148	KY817755	KY350154	N/A	Zhang et al. 2017
*N. sinoeucalypti*	CERC2005	*E. urophylla* × *E. grandis*	ZhanJiang Region, GuangDong Province, China	S.F. Chen & G.Q. Li	KX278061	KX278166	KX278270	KX278290	Li et al. 2018
	= CGMCC3.18752^c^								
	CERC3416	*Eucalyptus* hybrid	BeiHai Region, GuangXi Province, China	S.F. Chen & G.Q. Li	KX278064	KX278169	KX278273	KX278293	Li et al. 2018
	#NAME?								
*N. stellenboschiana*	CBS 110864^c^	*Vitis vinifera*	South Africa	F. Halleen	AY343407	AY343348	KX465047	KX464042	van Niekerk et al. 2004, Yang et al. 2017
*N. terminaliae*	CBS 125263	*Terminalia sericea*	South Africa	D. Begoude & J. Roux	GQ471802	GQ471780	KX465052	KX464045	Begoude 2010, Yang et al. 2017
	= CMW 26679^c^								
	CBS 125264	*Terminalia sericea*	South Africa	D. Begoude & J. Roux	GQ471804	GQ471782	KX465053	KX464046	Begoude 2010, Yang et al. 2017
	= CMW 26683								
*N. umdonicola*	CBS 123645	*Syzigium cordatum*	South Africa	D. Pavlic	EU821904	EU821874	EU821844	EU821934	Pavlic et al. 2009a, 2009b
	= CMW 14058^c^								
	CBS 123646	*Syzigium cordatum*	South Africa	D. Pavlic	EU821905	EU821875	EU821845	EU821935	Pavlic et al. 2009a, 2009b
	= CMW 14060								
*N. ursorum*	CMW 24480	*Eucalyptus* trees	South Africa	H.M. Maleme	FJ752746	FJ752709	KX465056	KX464047	Crous et al. 2013, Yang et al. 2017
	= CBS 122811^c^								
	CMW 23790	*Eucalyptus* trees	South Africa	H.M. Maleme	FJ752745	FJ752708	KX465057	N/A	Crous et al. 2013, Yang et al. 2017
*N. variabile*	CMW 37739^c^	*Mimusops caffra*	South Africa	M.J. Wingfield	MH558608	N/A	MH569153	N/A	Jami et al. 2018
	CMW 37742	*Mimusops caffra*	South Africa	M.J. Wingfield	MH558609	MH576585	MH569154	N/A	Jami et al. 2018
*N. viticlavatum*	CBS 112878	*Vitis vinifera*	South Africa	F. Halleen	AY343381	AY343342	KX465058	KX464048	Phillips et al. 2013, Yang et al. 2017
	= STE-U 5044^c^								
	CBS 112977	*Vitis vinifera*	South Africa	F. Halleen	AY343380	AY343341	KX465059	N/A	Phillips et al. 2013, Yang et al. 2017
	= STE-U 5041								
*N. vitifusiforme*	CBS 110887	*Vitis vinifera*	South Africa	J.M.van Niekerk	AY343383	AY343343	KX465061	KX464049	van Niekerk et al. 2004, Yang et al. 2017
	= STE-U 5252^c^								
	CBS 110880	*Vitis vinifera*	South Africa	J.M.van Niekerk	AY343382	AY343344	KX465008	N/A	van Niekerk et al. 2004, Yang et al. 2017
	= STE-U 5050								

^a^ *ALG* Personal culture collection A. Berraf-Tebbal, *ATCC* American Type Culture Collection, Virginia, USA, *BL* Personal number of B.T. Linaldeddu, *CAA* Personal culture collection Artur Alves, Universidade de Aveiro, Portugal, *CBS* CBS-KNAW Fungal Biodiversity Centre, Utrecht, The Netherlands, *CERC* Culture collection of China Eucalypt Research Centre, Chinese Academy of Forestry, ZhanJiang, GuangDong, China, *CFCC* China Forestry Culture Collection Center, Beijing, China, *CGMCC* China General Microbiological Culture Collection Center, Beijing, China, *CMM* Culture Collection of Phytopathogenic Fungi ‘Prof. Maria Menezes’, Universidade Federal Rural de Pernambuco, Recife, Brazil, *CMW* Tree Pathology Co-operative Program, Forestry and Agricultural Biotechnology Institute, University of Pretoria, South Africa, *CPC* Working collection of P.W. Crous, housed at CBS, *GZCC* Guizhou Academy of Agricultural Sciences Culture Collection, GuiZhou, China, *IBL* Personal culture collection, I.B.L. Coutinho, *IBP* Personal culture collection, I.B. Prasher, *ICMP* International Collection of Microorganisms from Plants, Auckland, New Zealand, *IRAN* Iranian Fungal Culture Collection, Iranian Research Institute of Plant Protection, Iran, *KUMCC* Kunming Institute of Botany Culture Collection, *MFLUCC* Mae Fah Luang University Culture Collection, Chiang Rai, Thailand, *MUCC* Culture collection of Murdoch University, Perth, Australia, *STE-U* Culture collection of the Department of Plant Pathology, University of Stellenbosch, South Africa, *UCD* University of California, Davis, Plant Pathology Department Culture Collection, *WAC* Department of Agriculture, Western Australia Plant Pathogen Collection, South Perth, Western Australia

^b^ *ITS* internal transcribed spacer; *tef1* translation elongation factor 1-alpha; *tub2* β-tubulin 2; *rpb2* DNA-directed RNA polymerase II subunit; *N/A* not available

^c^ Isolates represent ex-type are from samples that have been linked morphologically to type materials of the species

The criterion applied to determine species boundaries was based on phylogenetic analyses and sequences comparisons. Thus, species were considered unique when isolate(s) formed a distinct lineage that differentiated them from other isolates in at least two of the three or four individual loci (ITS, *tef1* and *tub2* for *Botryosphaeria*; or ITS, *tef1*, *tub2* and *rpb2* for *Lasiodiplodia* and *Neofusicoccum*). Furthermore, where these groupings were not contradicted at the other loci, and where they had fixed Single Nucleotide Polymorphisms (SNPs) that differentiated them from their phylogenetically closest species.

### Morphology

For the description of putatively novel species, microscopic features and colony characteristics were examined. More than one *Botryosphaeriaceae* species was frequently isolated from the pycnidia on the same *Eucalyptus* branch, and most of the isolates were obtained from diseased tissues, which were free of fruiting structures. Consequently, isolates were grown on Petri dishes containing 2% water agar (WA) with several double-autoclaved pine needles on their surface (Smith et al. 1996). These plates were incubated at room temperature under near-ultraviolet light for 4–6 wk. to induce sporulation. Relevant morphological characteristics were examined and recorded using a Zeiss Axio Imager A1 microscope and a Zeiss AxioCam MRc digital camera with Zeiss Axio Vision v.4.8 software (Carl Zeiss Ltd., Oberkochen, Germany). The lengths and widths of 50 conidia per isolate were measured. These are presented as average (mean), standard deviation (SD), minimum (min) and maximum (max) of the conidial measurements are presented as (min–) (mean–SD)–(mean + SD)(−max). The ratio of average length to average width (L/W) for each species was calculated. Morphological descriptions were deposited in MycoBank (www.mycobank.org).

To determine the optimum temperatures for growth of the novel species, a 5-mm-diam plug of agar was cut from the actively growing margin of a 7-day-old colony and placed at the centre of a 90-mm-diam Petri dish containing 2% MEA. Five replicate plates were used for each isolate at each temperature and these were incubated in the dark at temperatures ranging from 5 to 40 °C at 5 °C intervals. Two diameter measurements, perpendicular to each other, were recorded daily until the fastest growing culture reached the edge of the Petri dish. The average colony diameter for each of the eight temperatures was calculated. Colony colour was determined from 7-day-old cultures grown on 2% MEA at 25 °C using the colour charts of Rayner (1970).

### Pathogenicity tests

To determine the relative pathogenicity of the species identified in this study, inoculation trials were conducted under natural conditions using potted-trees of an *E. urophylla* × *E. grandis* hybrid clone and *E. globulus* seed-derived plants at the South China Experiment Nursery (SCEN), located in ZhanJiang, GuangDong, China. One-year-old healthy plants of the *E. urophylla* × *E. grandis* clone and *E. globulus* seed-derived plants, approximately 170 cm high and 2 cm diameter at the root collar, were utilised. For each plant, a 5-mm-diam wound was made on the stem (approximately 30 cm above the root collar) using a cork borer to remove the bark and expose the cambium. Seven-day-old cultures of representative isolates, representing different species of *Botryosphaeriaceae* incubated at 25 °C in the dark, were prepared and mycelial plugs were cut with a 5-mm-diam cork borer from the actively growing margins of these cultures. Mycelial plugs were placed into wounds with the mycelium facing the xylem. The wounds were sealed with masking tape immediately after inoculation to protect them from contamination and desiccation.

Ten trees of each *Eucalyptus* species were inoculated for each isolate. Negative controls were conducted on ten trees of the *E. urophylla* × *E. grandis* hybrid clone or *E. globulus* seed-derived plants with clean 2% MEA plugs. After one month, lesion lengths were measured and the average lesion length for the control treatments was subtracted from the average length for the fungus-treated plants. This measurement reflected the result of the fungal inoculation without including the wound response due to physical damage in the controls. Re-isolations were made from the inoculated plants to fulfil Koch’s postulates. General Linear Model (GLM) Univariate Analysis (two-way ANOVA) and one-way ANOVA were used to determine the differences in aggressiveness among isolates utilising the programmes SPSS v.20 (IBM Corp 2011) and SAS v.9.3 (SAS Institute Inc 2011), respectively for the two analyses.

## RESULTS

### Sample collection and fungal isolation

For each sampled tree, between one and five isolates of *Botryosphaeriaceae* were obtained. A total of 166 *Botryosphaeriaceae* isolates from 89 *Eucalyptus* trees were collected from the six regions (ChuXiong, HongHe, KunMing, PuEr, WenShan and YuXi) sampled (Table 1, Fig. 11). Of these, 76 isolates (45.8%) were from *E. urophylla* × *E. grandis*, including 23 isolates from 11 trees in the HongHe Region, 25 isolates were from 12 trees in the PuEr Region, 14 isolates from six trees in the WenShan Region and 14 isolates were from nine trees in the YuXi Region. Forty-nine isolates (29.5%) were from *E. globulus*, including 23 isolates from 18 trees in the ChuXiong Region, 16 isolates from eight trees in the HongHe Region and 10 isolates from four trees in the KunMing Region. Forty-one isolates (24.7%) were from 21 other unknown *Eucalyptus* species or hybrids in the HongHe Region.

### Phylogenetic analyses

The ITS, *tef1* and *tub2* loci were amplified for all the 166 isolates (Table 1). Subsequently, 82 representative isolates were selected based on these sequences so as to include all the genotypes revealed by these three loci, as well as all the sampling regions and *Eucalyptus* genotypes. The *rpb2* locus was then also sequenced for these 82 isolates (Table 1). The sequence fragments were approximately 520 bp for the ITS, 280 bp for the *tef1*, 430 bp for the *tub2* and 610 bp for the *rpb2*. The genotype of each isolate was determined based on the four loci, and one or two isolates were then selected for phylogenetic analyses, depending on the number of isolates available for each genotype (Table 1).

Based on the BLAST search against the nucleotide database on the NCBI website, three genera (*Botryosphaeria*, *Lasiodiplodia* and *Neofusicoccum*) in the *Botryosphaeriaceae* were identified. Sequences of ex-type isolates for all species in these genera were downloaded and used in the phylogenetic analyses. The aligned sequences for each locus (ITS, *tef1*, *tub2* and *rpb2*), as well as the combined sequences of three or four loci (*Botryosphaeria*: ITS, *tef1*, *tub2*; *Lasiodiplodia* and *Neofusicoccum*: ITS, *tef1*, *tub2*, *rpb2*) were deposited in TreeBASE (No. S25832). Statistical values for all datasets for ML and MP analyses are presented in Table 3. Isolates obtained in this study were divided into 11 groups (A to K) based on phylogenetic analyses. Single nucleotide polymorphism (SNP) analyses for the novel taxa emerging from this study and their closest sister taxa are presented in Table 4.

**Table 3:** Statistical values of datasets for maximum parsimony and maximum likelihood analyses

**Genus**	**Dataset**	**Maximum likelihood**									
		**Subst. model**^a^	**NST**^b^	**Rate matrix**					**p-inv**	**Gamma**	**Rates**
*Botryosphaeria*	ITS	TrN + I	6	1.0000	1.5461	1.0000	1.0000	5.4052	0.7570	–	Equal
	*tef1*	TVM + I	6	1.2703	4.1281	1.8345	0.0377	4.1281	0.5680	–	Equal
	*tub2*	TIM2 + G	6	0.2584	4.1669	0.2584	1.0000	8.5072	–	0.0280	Gamma
	*rpb2*	TPM3uf + I	6	2872.6267	37,884.9415	1.0000	2872.6267	37,884.9415	0.7290	–	Equal
	ITS/*tef1*/*tub2*	TrN + I	6	1.0000	3.6483	1.0000	1.0000	6.4337	0.7430	–	Equal
*Lasiodiplodia*	ITS	TPM1uf + I + G	6	1.0000	8.3069	3.1151	3.1151	8.3069	0.6640	0.7300	Gamma
	*tef1*	TrN + G	6	1.0000	3.1913	1.0000	1.0000	5.0207	–	0.4440	Gamma
	*tub2*	TIM3 + G	6	2.6726	3.8861	1.0000	2.6726	10.7258	–	0.4200	Gamma
	*rpb2*	TrN + I + G	6	1.0000	4.7971	1.0000	1.0000	13.7321	0.4690	1.8510	Gamma
	ITS/*tef1*/*tub2*/*rpb2*	TIM2 + I + G	6	1.2861	4.0643	1.2861	1.0000	8.3643	0.5010	0.6480	Gamma
*Neofusicoccum*	ITS	TIM1 + I + G	6	1.0000	10.7228	2.7330	2.7330	23.3748	0.5420	0.5670	Gamma
	*tef1*	TPM2uf + G	6	1.6352	7.1729	1.6352	1.0000	7.1729	–	0.6840	Gamma
	*tub2*	TIM3 + G	6	1.9226	7.3114	1.0000	1.9226	12.7028	–	0.2070	Gamma
	*rpb2*	TIM3 + G	6	2.4608	9.3031	1.0000	2.4608	24.9646	–	0.2660	Gamma
	ITS/*tef1*/*tub2*/*rpb2*	TrN + I + G	6	1.0000	5.0967	1.0000	1.0000	9.7420	0.4430	0.7340	Gamma
**Genus**	**Dataset**	**No. of taxa**	**No. of bp**^c^	**Maximum parsimony**							
				**PIC**^d^	**No. of trees**	**Tree length**	**CI**^e^	**RI**^f^	**RC**^g^	**HI**^h^	
*Botryosphaeria*	ITS	49	530	30	86	51	0.8039	0.8913	0.7165	0.1961	
	*tef1*	49	353	115	120	152	0.8684	0.9385	0.8150	0.1316	
	*tub2*	42	414	22	35	30	0.8000	0.9063	0.7250	0.2000	
	*rpb2*	30	718	23	4	37	0.8378	0.9483	0.7945	0.1622	
	ITS/*tef1*/*tub2*	49	1297	167	234	241	0.8174	0.9085	0.7427	0.1826	
*Lasiodiplodia*	ITS	74	511	50	5000	91	0.6813	0.8858	0.6035	0.3187	
	*tef1*	73	323	135	1233	415	0.6024	0.8922	0.5375	0.3976	
	*tub2*	64	409	41	5000	60	0.7667	0.9310	0.7138	0.2333	
	*rpb2*	53	532	104	3297	192	0.6354	0.8649	0.5496	0.3646	
	ITS/*tef1*/*tub2*/*rpb2*	74	1775	330	3989	854	0.5621	0.8508	0.4782	0.4379	
*Neofusicoccum*	ITS	99	535	86	1790	205	0.5512	0.8844	0.4875	0.4488	
	*tef1*	98	307	150	5000	312	0.7308	0.9413	0.6879	0.2692	
	*tub2*	98	424	72	1380	149	0.6040	0.8952	0.5407	0.3960	
	*rpb2*	76	605	116	2619	201	0.6915	0.9180	0.6348	0.3085	
	ITS/*tef1*/*tub2*/*rpb2*	101	1871	424	3584	936	0.6090	0.8968	0.5461	0.3910	

^a^ *Subst. model* = best fit substitution model

^b^ *NST* Number of substitution rate categories

^c^ *bp* Base pairs

^d^ *PIC* Number of parsimony informative characters

^e^ *CI* Consistency index

^f^ *RI* Retention index

^g^ *RC* Rescaled consistency index

^h^ *HI* Homoplasy index

**Table 4:** Number of fixed SNPs between newly described species and their phylogenetically close taxa

Species	Single nucleotide polymorphism comparisons of four loci					
	*B. puerensis*	*N. dianense*	*N. magniconidium*	*N. ningerense*	*N. parviconidium*	*N. yunnanense*
*Botryosphaeria corticis*	13/16/14/*^a^	—	—	—	—	—
*B. fabicerciana*	1/14/9/12	—	—	—	—	—
*B. fusispora*	1/16/11/*	—	—	—	—	—
*B. kuwatsukai*	1/10/*/*	—	—	—	—	—
*B. qingyuanensis*	2/9/9/3	—	—	—	—	—
*B. rosaceae*	1/13/9/*	—	—	—	—	—
*Neofusicoccum algeriense*	—^b^	3/1/7/*	—	—	—	3/1/4/*
*N. dianense*	—	—	—	—	—	2/2/3/6
*N. hongkongense*	—	4/0/2/4	—	—	—	4/2/1/2
*N. italicum*	—	4/0/5/5	—	—	—	4/1/*/*
*N. macroclavatum*	—	—	9/1/6/2	7/1/6/2	—	—
*N. mangiferae*	—	—	—	—	2/5/2/27	—
*N. microconidium*	—	—	—	—	1/3/1/1	—
*N. ningerense*	—	—	2/0/2/2	—	—	—
*N. parvum*	—	3/2/5/5	—	—	—	1/4/2/0

^a^ The number means the difference of two species in four loci, ITS/*tef1*/*tub2*/*rpb2*; “*” represents the sequence is unavailable

^b^ “—” represent the sequences between two species were not compared

### *Species in* Botryosphaeria

**Fig. 2:**
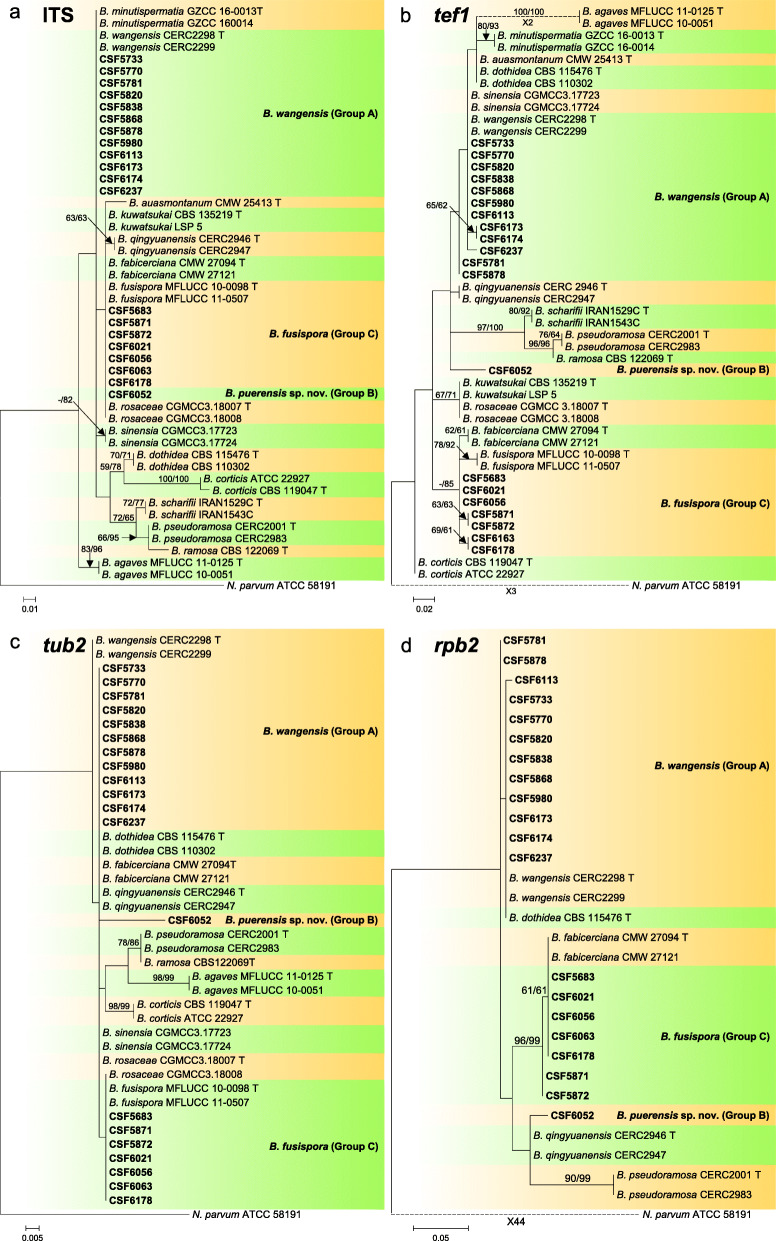

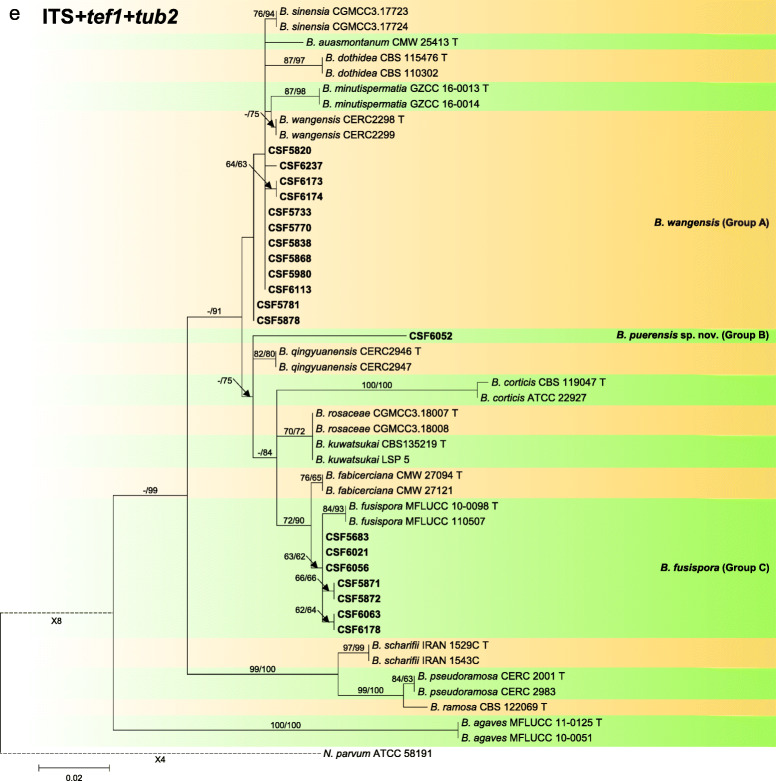
Phylogenetic trees based on maximum likelihood (ML) analyses for species in *Botryosphaeria*. **a**. ITS; **b**. *tef1*; **c**. *tub2*; **d**. *rpb2*; **e**. combination of ITS, *tef1* and *tub2*. Isolates sequenced in this study are in bold. Bootstrap support values ≥60% for ML and MP are presented above branches as follows: ML/MP, bootstrap support values < 60% are marked with ‘-’, and absent are marked with ‘*’. Ex-type isolates are marked with ‘T’. The trees were rooted to *N. parvum* (ATCC 58191)

Sequence data were not available for *rpb2* for ex-type isolates of various *Botryosphaeria* species (Table 2). The *Botryosphaeria* isolates clustered in three groups (Group A, Group B and Group C) based on *tef1*, *tub2*, *rpb2* and combined ITS/*tef1*/*tub2* analyses, and two groups based on ITS analyses, including Group A and where Group B clustered with Group C (Fig. 2).

Isolates in Group A clustered with *B. wangensis* and *B. minutispermatia* based on phylogenetic analyses of ITS dataset (Fig. 2a). In the *tef1* tree, they clustered with or were closely related to *B. wangensis*, *B. auasmontanum*, *B. dothidea*, *B. minutispermatia* and *B. sinensia* (Fig. 2b). In the *tub2* tree, they clustered with *B. dothidea*, *B. fabicerciana*, *B. qingyuanensis*, *B. rosaceae* and *B. sinensia*, and were closely related to *B. wangensis* (Fig. 2c). In the *rpb2* tree, they clustered with or were closely related to *B. wangensis* and *B. dothidea* (Fig. 2d). In the combined ITS/*tef1*/*tub2* tree, these isolates were closely related to *B. wangensis* (Fig. 2e). Some isolates formed an independent clade based on one of the four individual loci (isolates CSF6173 and CSF6174 in the *tef1* tree, isolate CSF6237 in the *tef1* tree, and isolate CSF6113 in the *rpb2* tree) (Fig. 2b–d); isolates CSF5781 and CSF5878 formed an independent clade based on two loci (*tef1* and *rpb2* trees) (Fig. 2b, d), while they only had three fixed SNPs (one in each of ITS, *tef1* and *tub2* loci, respectively) different to the phylogenetically closest species, *B. wangensis*. Based on the phylogenetic analyses for the different datasets and fixed SNPs difference, isolates in Group A were identified as *B. wangensis*.

Isolate CSF6052 in Group B clustered with *B. fabicerciana*, *B. fusispora*, *B. kuwatsukai* and *B. rosaceae* based on the ITS tree (Fig. 2a). This isolate formed an independent clade that was distinct from all known species based on the *tef1*, *tub2*, *rpb2* and the combined ITS/*tef1*/*tub2* trees (Fig. 2b–e). There were also 23 fixed SNPs different to its phylogenetically closest species, *B. qingyuanensis*. Consequently, isolate CSF6052 was recognised as an undescribed species.

Isolates in Group C clustered with *B. fusispora*, *B. fabicerciana*, *B. kuwatsukai*, *B. puerensis* and *B. rosaceae* in the ITS tree (Fig. 2a). They were closely related to *B. fusispora* and *B. fabicerciana* in the *tef1* tree (Fig. 2b) and clustered with *B. fusispora* in the *tub2* tree (Fig. 2c). They clustered with or were close to *B. fabicerciana* in the *rpb2* tree, but could not be compared with *B. fusispora* because sequence data for this region are not available for that species (Fig. 2d). Based on *tef1* data (Fig. 2b), three independent clades emerged accommodating isolates CSF5683, CSF6021 and CSF6056; CSF5871 and CSF5872; and CSF6063 and CSF6178, but they had only three or four fixed SNPs different to their phylogenetically closest species *B. fusispora*. These isolates in Group C were phylogenetically close to *B. fusispora* based on ITS, *tef1*, *tub2* and the combined ITS/*tef1*/*tub2* trees (Fig. 2) and they were identified as that species.

### *Species in* Lasiodiplodia

**Fig. 3:**
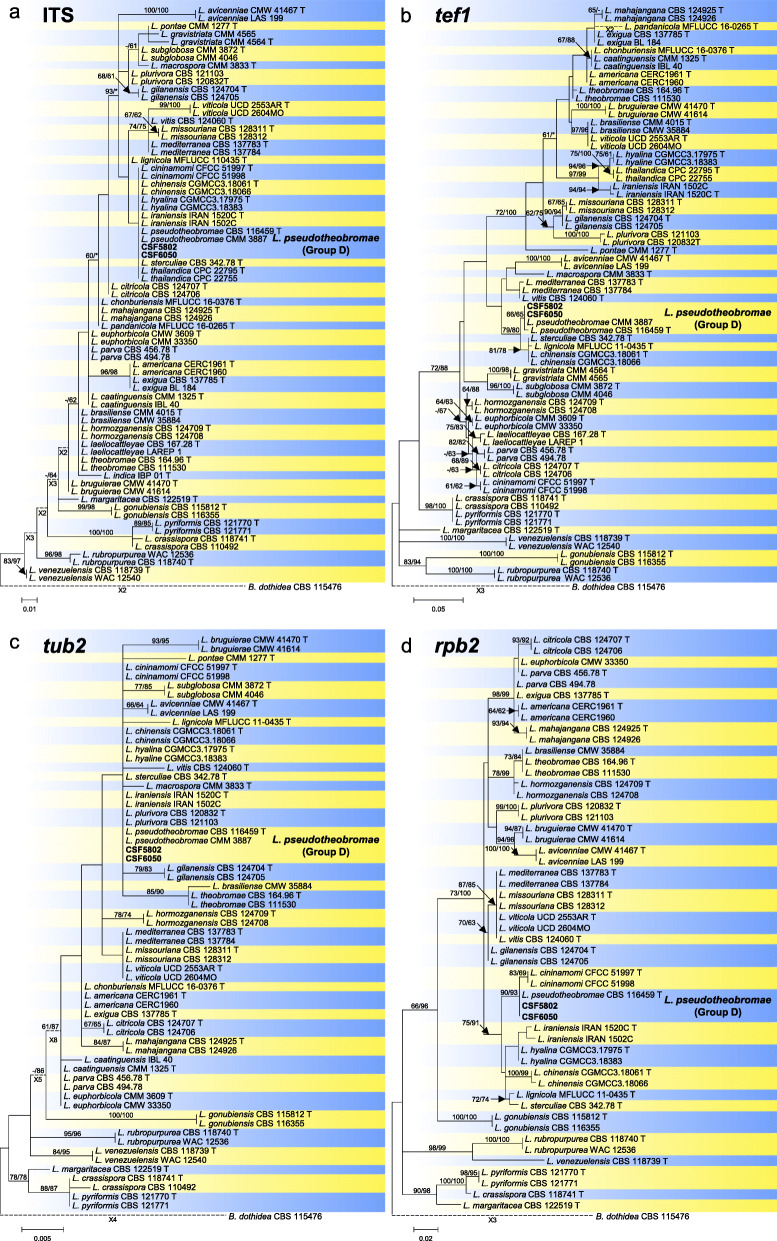

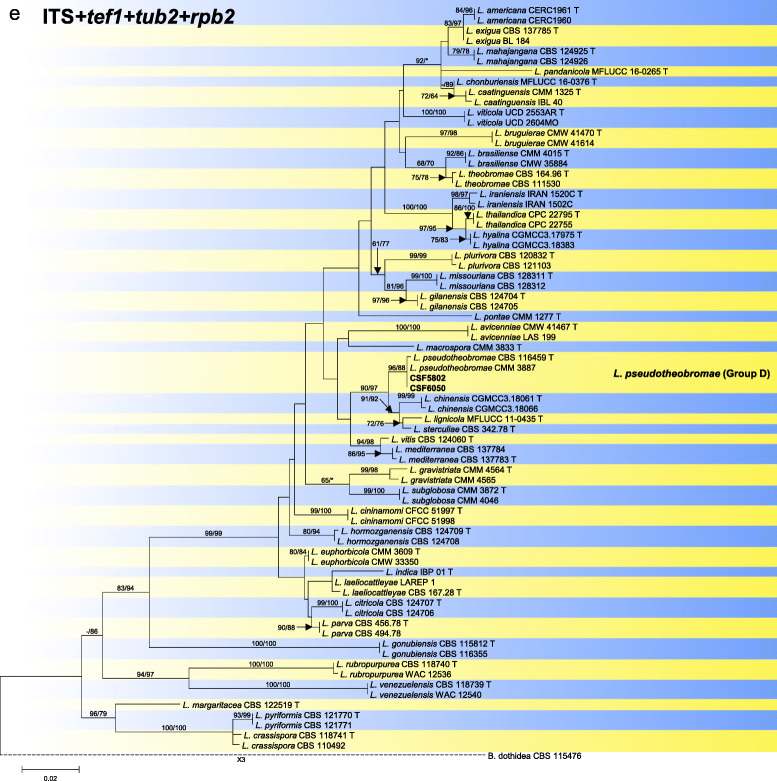
Phylogenetic trees based on maximum likelihood (ML) analyses for species in *Lasiodiplodia*. **a**. ITS; **b**. *tef1*; **c**. *tub2*; **d**. *rpb2*; **e**. combination of ITS, *tef1*, *tub2* and *rpb2*. Isolates sequenced in this study are in bold. Bootstrap support values ≥60% for ML and MP are presented above branches as follows: ML/MP, bootstrap values < 60% are marked with ‘-’, and absent are marked with ‘*’ Ex-type isolates are marked with ‘T’. The trees were rooted to *B. dothidea* (CBS 115476)

Analyses were conducted for *Lasiodiplodia* based on sequences for the ITS, *tef1*, *tub2* and *rpb2* loci. Based on phylogenetic analyses for these loci and the combined ITS/*tef1*/*tub2*/*rpb2* datasets, two *Lasiodiplodia* isolates clustered in one group (Group D) (Fig. 3). These isolates were phylogenetically related to *L. pseudotheobromae* and various other species based on ITS and *tub2* trees (Fig. 3a, c). They were closest *L. pseudotheobromae* based on *tef1* tree (Fig. 3b), and clustered with *L. pseudotheobromae* based on *rpb2* tree (Fig. 3d). The tree based on the combined ITS/*tef1*/*tub2*/*rpb2* dataset also showed that the two isolates making up Group D were phylogenetically closely related to *L. pseudotheobromae* and they were treated as that species (Fig. 3e).

### *Species in* Neofusicoccum

**Fig. 4:**
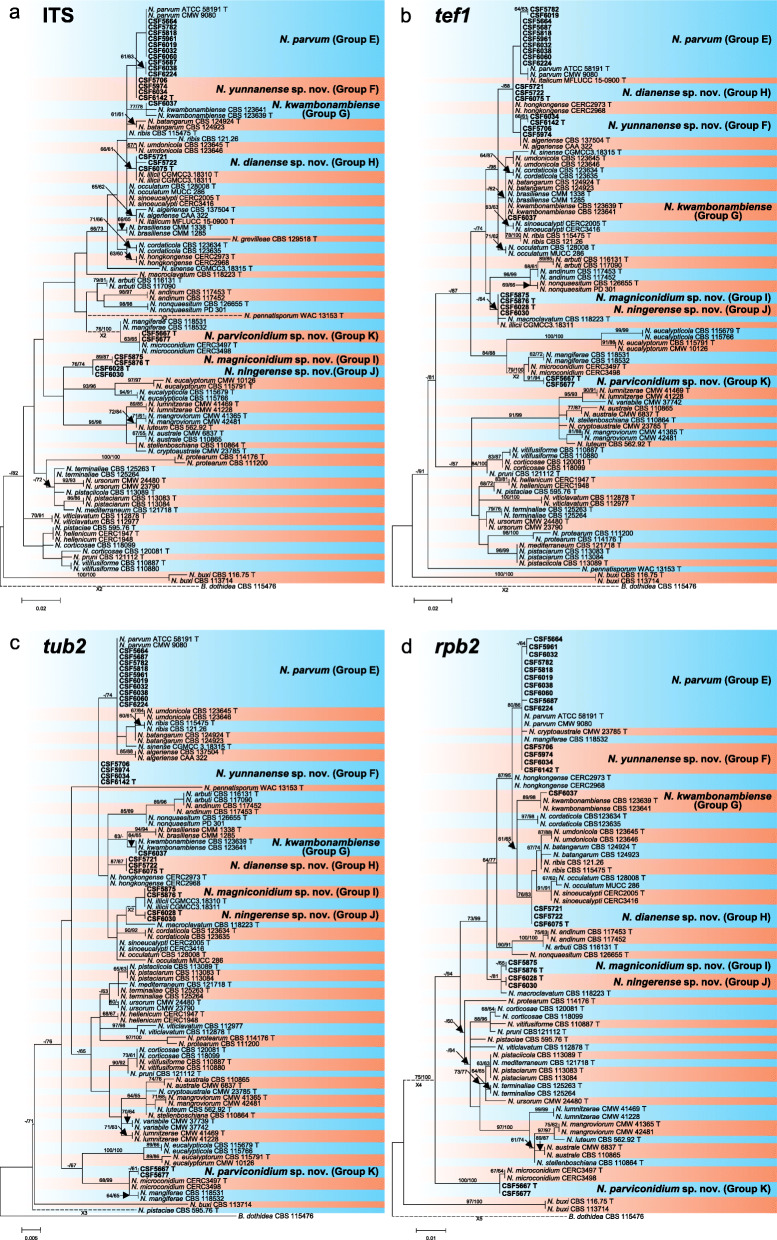

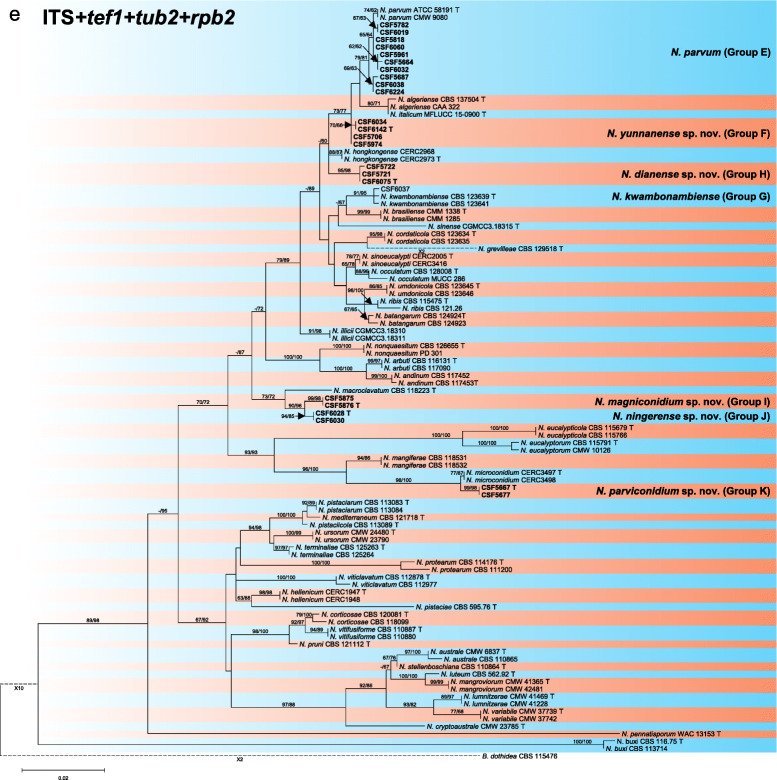
Phylogenetic trees based on maximum likelihood (ML) analyses for species in *Neofusicoccum*. **a**. ITS; **b**. *tef1*; **c**. *tub2*; **d**. *rpb2*; **e**. combination of ITS, *tef1*, *tub2* and *rpb2*. Isolates sequenced in this study are in bold. Bootstrap support values ≥60% for ML and MP are presented above branches as follows: ML/MP, bootstrap support values < 60% are marked with ‘-’, and absent are marked with ‘*’. Ex-type isolates are marked with ‘T’. The trees were rooted to *B. dothidea* (CBS 115476)

The *Neofusicoccum* isolates resided in seven groups based on ITS, *tub2* and the combined ITS/*tef1*/*tub2*/*rpb2* datasets (Groups E–K). For the *tef1* dataset, there were six groups including Groups E–H, Group I that clustered with Group J and Group K. For the *rpb2* dataset, there were six groups including Group E that clustered with Group F and Groups G–K (Fig. 4).

Isolates in Group E were closely related to *N. parvum* and various other species based on the ITS, *tef1* and *rpb2* trees (Fig. 4a, b, d) and they also clustered with *N. parvum* based on the *tub2* tree (Fig. 4c). They formed multiple independent clades based on the ITS, *tef1*, *rpb2* and the combined ITS/*tef1*/*tub2*/*rpb2* trees (Fig. 4a, b, d, e). Based on these analyses of five datasets, isolates in Group E were treated as *N. parvum* (Fig. 4).

Isolates in Group F were closely related to *N. algeriense* based on phylogenetic analyses of *tef1* dataset (Fig. 4b). They clustered with *N. mangiferae* and *N. parvum* in the *rpb2* tree (Fig. 4d). Isolates in Group F formed one independent clade that was distinct from all known species based on ITS and *tub2* trees, and isolates CSF6034 and CSF6142 (ex-type) formed a distinct lineage in *tef1* tree (Fig. 4a–c). In the combined tree, isolates CSF6034 and CSF6142 (ex-type), and other isolates in Group F formed an independent sub-clade (Fig. 4e). Seven fixed SNPs also differentiated isolates in Group F from their phylogenetically closest relatives *N. algeriense* and *N. parvum* in the ITS, *tef1* and *tub2* regions, and five fixed SNPs differentiated them from *N. italicum* in the ITS and *tef1* regions (*tub2* not available for *N. italicum*) (Table 4). These isolates were consequently treated as representing a novel species.

Isolate CSF6037 in Group G clustered with *N. kwambonambiense* in the *tub2* tree (Fig. 4c). It also clustered with *N. kwambonambiense* and various other species in the *tef1* tree (Fig. 4b), and was most closely related to that species in the ITS, *rpb2* and the combined ITS/*tef1*/*tub2*/*rpb2* trees (Fig. 4a, d, e). Isolate CSF6037 was consequently identified as *N. kwambonambiense.*

Isolates in Group H clustered with *N. illicii* in the ITS tree (Fig. 4a) and with *N. hongkongense* in the *tef1* tree (Fig. 4b). Based on the *tub2* and *rpb2* trees, these isolates formed an independent clade that was distinct from all known species of *Neofusicoccum* (Fig. 4c, d). This clade was well supported by high bootstrap values in the *tub2* and combined ITS/*tef1*/*tub2*/*rpb2* trees (*tub2*, ML/MP = 87%/87%; ITS/*tef1*/*tub2*/*rpb2*, ML/MP = 95%/98%) (Fig. 4c, e). There were also ten fixed SNPs differentiating isolates in Group H from their phylogenetically closest species, *N. hongkongense* (Table 4). Consequently, isolates in Group H were considered to represent a novel species of *Neofusicoccum*.

Isolates in both Group I and Group J formed a single clade that clustered with *N. illicii* in the *tef1* tree, and isolates in Group I clustered with *N. illicii* in the *tub2* tree (Fig. 4c). But isolates in these two groups formed two independent clades in the ITS and *rpb2* trees (Fig. 4a, d), and those in Group J also formed an independent clade in the *tub2* tree (Fig. 4c). The two independent clades were supported by high bootstrap values in the combined ITS/*tef1*/*tub2*/*rpb2* tree (Group I, ML/MP = 99%/98%; Group J, ML/MP = 94%/85%) (Fig. 4e). In addition, there were six fixed SNPs observed between isolates in Group I and Group J (Table 4). Thus, isolates in Group I and Group J were considered to represent two undescribed species of *Neofusicoccum*.

Isolates in Group K clustered with *N. microconidium* in the ITS tree (Fig. 4a). However, they formed a distinct clade that was separated from all known species in the *tef1*, *tub2*, and *rpb2* trees (Fig. 4b–d). These isolates resided in a single clade, which was supported by high bootstrap values in the combined ITS/*tef1*/*tub2*/*rpb2* tree (ML/MP = 99%/98%) (Fig. 4e). There were also six fixed SNPs observed between isolates in Group K and their phylogenetically closest relative, *N. microconidium* (Table 4). Consequently, isolates in Group K were considered to represent a novel species.

### Morphology and taxonomy

Based on analyses of DNA sequence data, the isolates obtained in the present study clustered in 11 phylogenetic groups of the *Botryosphaeriaceae*. The culture morphology of all isolates in these groups was morphologically similar to other species of *Botryosphaeriaceae*, consistent with the fact that this characteristic has little taxonomic significance.

Isolates representing Groups B, F and H–K were identified as novel species based on the phylogenetic analyses. Representative isolates for these groups were selected to induce fruiting structures (Table 1). With the exception of those in Group J (isolates CSF6028 and CSF6030), that did not sporulate, these putatively novel taxa produced only asexual structures. Morphological differences were observed for the phylogenetically distinct species (Table 5) and these have been included in their descriptions. Based primarily on phylogenetic inference but including available morphological characteristics, isolates in Groups B, F, H–K were recognised as representing six previously undescribed species for which names are proposed as follows:

**Table 5:** Conidial measurements of *Botryosphaeriaceae* species described in this study and comparison with phylogenetically close species in previous studies

Species^a^	Conidial size (μm) (L × W)^b^	Mean (μm) (L × W)^c^	L*/*W^d^	Reference
*Botryosphaeria corticis*	(20.5–)23.5–32.5(−34.5) × (5.0–)5.5–7(−7.5)	28.9 × 6.4	4.5	Phillips et al. 2006
*B. fabicerciana*	(16.5–)19.5–24.5(−26) × (4.5–)5–6.5(−7.5)	22.0 × 5.8	3.8	Chen et al. 2011
*B. fusispora*	16–22 × 4–5.5	20.0 × 5.0	4.0	Liu et al. 2012
*B. kuwatsukai*	(18.5**–**)20**–**24.5(**−**26) × 5**–**7(**−**8)	22.3 × 6.2	3.6	Xu et al. 2015
***B. puerensis***^a^	**(22.5–)24–29.5(−32) × (4.5–)5.5–7.5(− 8)**	**26.8 × 6.4**	**4.2**	**This study**
*B. qingyuanensis*	(15–)19.5–24.5(−28.5) × (5–)6–6.5(− 7.5)	22.0 × 6.2	3.5	Li et al. 2018
*B. rosaceae*	20–31 × 6–8	26.2 × 6.7	3.9	Zhou et al. 2017
*Neofusicoccum algeriense*	(14.5**–**)17**–**18(**−**21) × (4.5**–**)5.5**–**5.7(**−**6.5)	17.6 × 5.6	3.1	Berraf-Tebbal et al. 2014
***N. dianense***^a^	**(16–)16.5–21(−24) × (4.5–)5–5.5(− 6)**	**18.9 × 5.2**	**3.6**	**This study**
*N. hongkongense*	(11.5–)13–15.5(−17.5) × (4–)4.5–5(−5.5)	14.1 × 4.7	3.0	Li et al. 2018
*N. italicum*	13–18.5 × 3.5–6	15.8 × 5.2	—^e^	Marin-Felix et al. 2017
*N. macroclavatum*	(19–)25–35(−41) × (5–)6–8(−10)	30.3 × 7.1	4.2	Burgess et al. 2005
***N. magniconidium***^a^	**(27–)27.5–30(−34) × (5.5–)6–7.5(−8)**	**29.1 × 6.7**	**4.3**	**This study**
*N. mangiferae*	(11–)12–15(−17.5) × 5–6.6	13.6 × 5.4	2.0**–**2.5	Slippers et al. 2005
*N. microconidium*	(10–)11.5–13(−14.5) × (4–)4.5–5.5(−6)	12.3 × 5.0	2.5	Li et al. 2018
***N. parviconidium***^a^	**(9.5–)10.5–11.5(−12.5) × (4.4–)5–5.5(− 6)**	**10.9 × 5.2**	**2.1**	**This study**
*N. parvum*	(12–)13.5–21(−24) × 4–6(− 10)	17.1 × 5.5	3.2	Phillips et al. 2013
***N. yunnanense***^a^	**(13–)13.5–17.5(−20) × (3.5–)4–4.5(− 5)**	**15.6 × 4.4**	**3.5**	**This study**

^a^ Species in bold are novel species described in this study

^b^ Minimum–(average – standard deviation)–(average + standard deviation)–maximum or minimum–maximum, L × W = length × width

^c^ L × W = average length × average width

^d^ L / W = average length/average width

^e^ “—” indicates no data was available

***Botryosphaeria puerensis*** G.Q. Li & S.F. Chen, **sp. nov.**

**Fig. 5:**
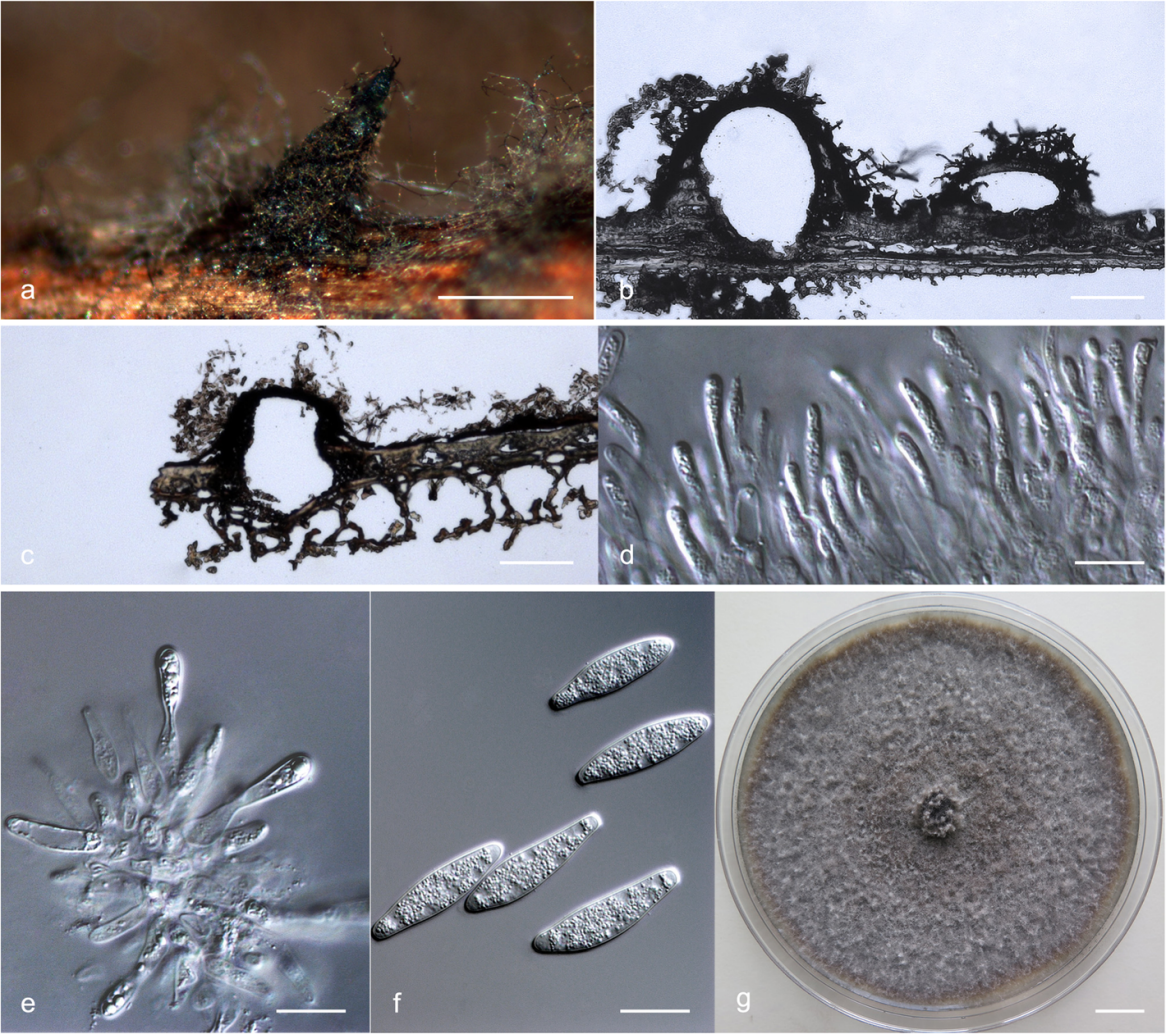
*Botryosphaeria puerensis.* **a**. Conidiomata formed on pine needle culture; **b**, **c**. Longitudinal section through conidiomata; **d**, **e**. Conidiogenous cells and developing conidia; **f**. Conidia; **g**. Living culture after 10 d on 2% MEA (front). Scale bars: a = 500 μm; b, c = 100 μm; d–f = 10 μm; g = 1 cm

MycoBank MB834102. (Fig. 5).

*Etymology*: Name reflects the PuEr Region where the fungus was isolated for the first time.

*Diagnosis*: *Botryosphaeria puerensis* produces shorter conidia than *B. corticis*, but longer conidia than other species of *Botryosphaeria*.

*Type*: **China**: YunNan Province, PuEr Region, JingGu County (GPS 23°20′21″N, 100°54′38″E), from twigs of one *E. urophylla × E. grandis* tree, 16 November 2014, *S.F. Chen & G.Q. Li*, fruiting structures induced on needles of *Pinus* sp. on water agar (HMAS255719 – holotype, CSF6052 = CGMCC3.20081 – ex-type culture).

*Description*: *Sexual state* unknown*. Conidiomata* pycnidial, produced on pine needles on WA medium within 4–6 wk., globose to ovoid, dark brown to black, up to 662 μm wide, 1041 μm high, embedded in needle tissue, semi-immersed to superficial, unilocular, with a central ostiole. *Conidiophores* reduced to conidiogenous cells. *Conidiogenous cells* holoblastic, discrete, hyaline, cylindrical to lageniform, phialidic with periclinal thickening, (6–)7–14(− 20) × (1.5–)2–3.5(− 4) μm. *Paraphyses* not seen. *Conidia* hyaline, thin-walled, smooth with granular contents, unicellular, aseptate narrowly fusiform, base subtruncate to bluntly rounded, (22.5–)24–29.5(− 32) × (4.5–)5.5–7.5(− 8) μm (av. of 100 conidia 26.8 × 6.4 μm; L/W = 4.2) (Table 5).

*Culture characteristics*: Colonies on MEA medium having fluffy mycelia with uneven margins and a few cottony aerial mycelia reaching to the lids of Petri plates, mycelial mat appressed, sparse to moderately dense. Colony mycelia initially white, becoming smoke gray (19”“f) to olivaceous (21”k) at the surface and olivaceous gray (23”“‘b) to iron gray (23”“‘k) at the reverse after 10 d. Optimal growth temperature 25 °C. No growth at 5 °C and 40 °C. After 4 d, colonies at 10 °C, 15 °C, 20 °C, 25 °C, 30 °C and 35 °C reaching 14 mm, 31 mm, 43 mm, 64 mm, 62 mm and 10 mm, respectively.

*Host*: *E. urophylla × E. grandis*.

*Distribution*: Currently only known from PuEr Region in YunNan Province, China.

*Notes*: *Botryosphaeria puerensis* is phylogenetically closely related to *B. corticis*, *B. fabicerciana*, *B. fusispora*, *B. kuwatsukai*, *B. rosaceae* and *B. qingyuanensis* (Fig. 2). Conidia (Table 5) of *B. puerensis* (av. 26.8 × 6.4; L/W = 4.2) are larger than in those species with the exception of *B. corticis* (av. 28.9 × 6.4; L/W = 4.5) (Phillips et al. 2006; Chen et al. 2011; Liu et al. 2012; Xu et al. 2015; Zhou et al. 2017; Li et al. 2018).

***Neofusicoccum dianense*** G.Q. Li & S.F. Chen, **sp. nov.**

**Fig. 6:**
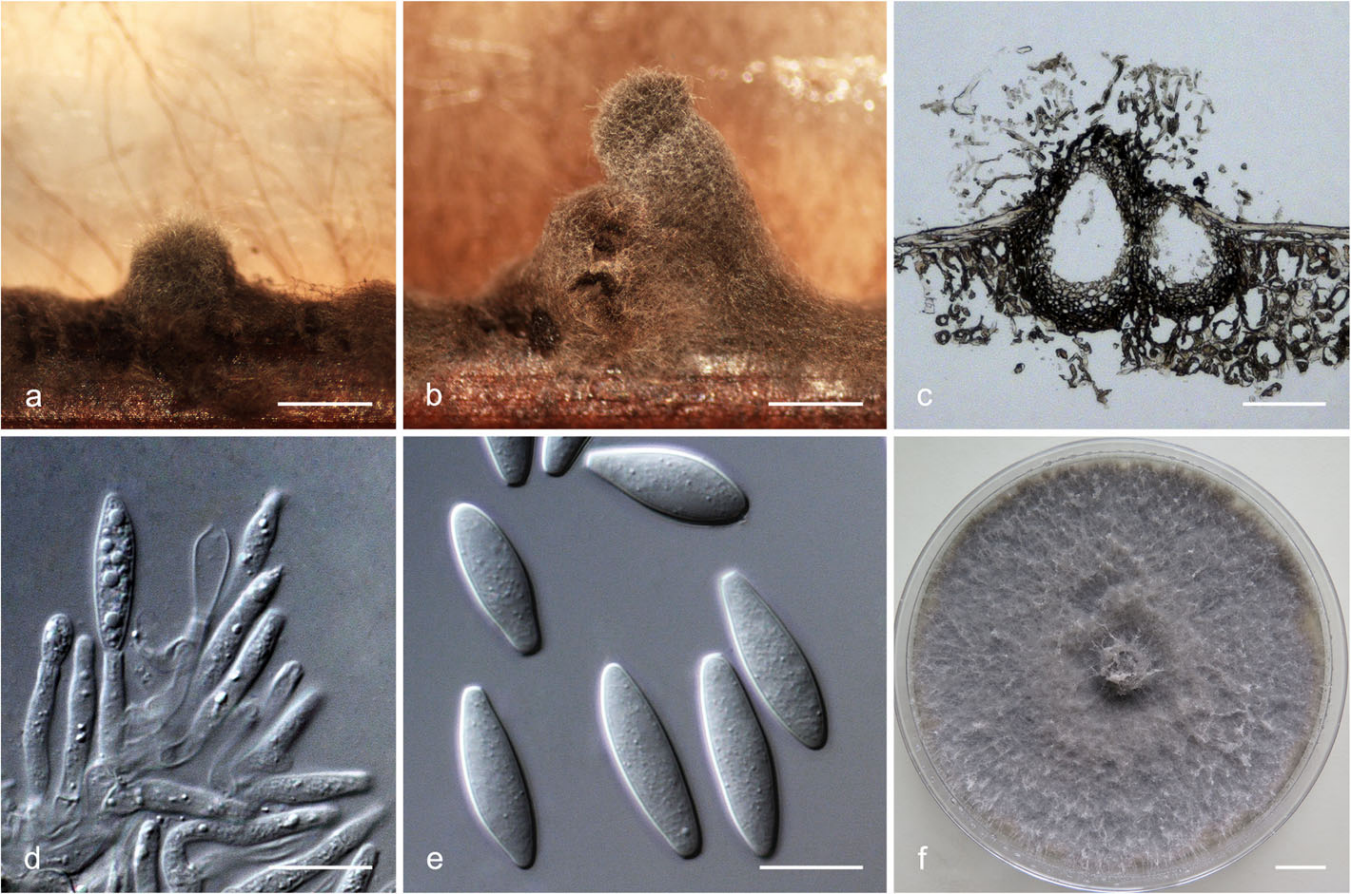
*Neofusicoccum dianense.* **a**, **b**. Conidiomata formed on pine needle culture; **c**. Longitudinal section through conidiomata; **d**. Conidiogenous cells and developing conidia; **e**. Conidia; **f**. Living culture after 10 d on 2% MEA (front). Scale bars: a, b = 500 μm; c = 100 μm; d, e = 10 μm; f = 1 cm

MycoBank MB834103. (Fig. 6).

*Etymology*: Name refers to “Dian”, an ancient kingdom of YunNan Province, where the type specimen was collected.

*Diagnosis*: Based on phylogenetic inference, *Neofusicoccum dianense* resides in ‘*N. parvum* / *N. ribis*’ complex. It produces the longer conidia than its closest phylogenetic relatives including *N. algeriense*, *N. hongkongense*, *N. italium*, *N. parvum*, *N. yunnanense*. The optimal growth temperature of *N. dianense* also differs from that of *N. yunnanense*.

*Type*: **China**: YunNan Province, PuEr Region, JingGu County (GPS 23°23′58″N, 100°50′37″E), from twigs of one *E. urophylla × E. grandis* tree, 16 November 2014, *S.F. Chen & G.Q. Li*, fruiting structures induced on needles of *Pinus* sp. on water agar (HMAS255720 – holotype, CSF6075 = CGMCC3.20082 – ex-type culture).

*Description*: *Sexual state* unknown*. Conidiomata* pycnidial, produced on pine needles on WA medium within 4–6 wk., globose to ovoid, dark brown to black, up to 1363 μm wide, 2298 μm high, embedded in needle tissue, semi-immersed to superficial, unilocular, with a central ostiole. *Conidiophores* reduced to conidiogenous cells. *Conidiogenous cells* holoblastic, discrete, hyaline, cylindrical to lageniform, phialidic with periclinal thickening, (8.5–)10.5–15(− 16.5) × (2–)2.5–3(− 3.5) μm. *Paraphyses* not seen. *Conidia* hyaline, thin-walled, smooth with granular contents, unicellular, aseptate narrowly fusiform, base subtruncate to bluntly rounded, (16–)16.5–21(− 24) × (4.5–)5–5.5(− 6) μm (av. of 100 conidia 18.9 × 5.2 μm; L/W = 3.6) (Table 5).

*Culture characteristics*: Colonies on MEA medium with fluffy mycelia with uneven margins and a few cottony aerial mycelia reaching to the lids of Petri plates, mycelial mat appressed, sparse to moderately dense. Colony mycelia initially white, becoming pale mouse grey (15”“‘d) to mouse grey (13”“‘i) at the surface and olivaceous grey (23”“‘b) to iron grey (23”“‘k) at the reverse after 10 d. Optimal growth temperature 25 °C. No growth at 5 °C and 40 °C. After 4 d, colonies at 10 °C, 15 °C, 20 °C, 25 °C, 30 °C and 35 °C reaching 16 mm, 47 mm, 71 mm, 86 mm, 73 mm and 12 mm, respectively.

*Host*: *E. globulus*, *E. urophylla × E. grandis* and *Eucalyptus* sp.

*Distribution*: Currently known from PuEr and HongHe Regions in YunNan Province, China.

*Notes*: *Neofusicoccum dianense* is phylogenetically closely related to *N. algeriense*, *N. hongkongense*, *N. italium*, *N. parvum* and *N. yunnanense* (Fig. 4). The conidia (Table 5) of *N. dianense* (av. 18.9 × 5.2; L/W = 3.6) are larger than those of *N. hongkongense* (av. 14.1 × 4.7; L/W = 3.0; Li et al. 2018) and *N. yunnanense* (av. 15.6 × 4.4; L/W = 3.5), and longer than those of *N. algeriense* (av. 17.6 × 5.6; L/W = 3.1; Berraf-Tebbal et al. 2014), *N. italium* (av. 15.8 × 5.2; L/W = 3.0; Marin-Felix et al. 2017) and *N. parvum* (av. 17.1 × 5.5; L/W = 3.2; Phillips et al. 2013).

*Additional specimens examined*: **China**: YunNan Province, HongHe Region, PingBian County (GPS 23°05′36″N, 103°31′52″E), from twigs of one *E. globulus* tree, 13 November 2014, *S.F. Chen* & *G.Q. Li*, fruiting structures induced on needles of *Pinus* sp. on water agar (HMAS255721, culture CSF5721 = CGMCC3.20075); YunNan Province, HongHe Region, PingBian County (GPS 23°05′36″N, 103°31′52″E), from twigs of one *E. globulus* tree, 13 November 2014, *S.F. Chen* & *G.Q. Li* (culture CSF5722); YunNan Province, HongHe Region, MengZi County (GPS 23°12′24″N, 103°30′58″E), from twigs of one *Eucalyptus* tree, 14 November 2014, *S.F. Chen* & *G.Q. Li* (culture CSF5840).

***Neofusicoccum magniconidium*** G.Q. Li & S.F. Chen, **sp. nov.**

**Fig. 7:**
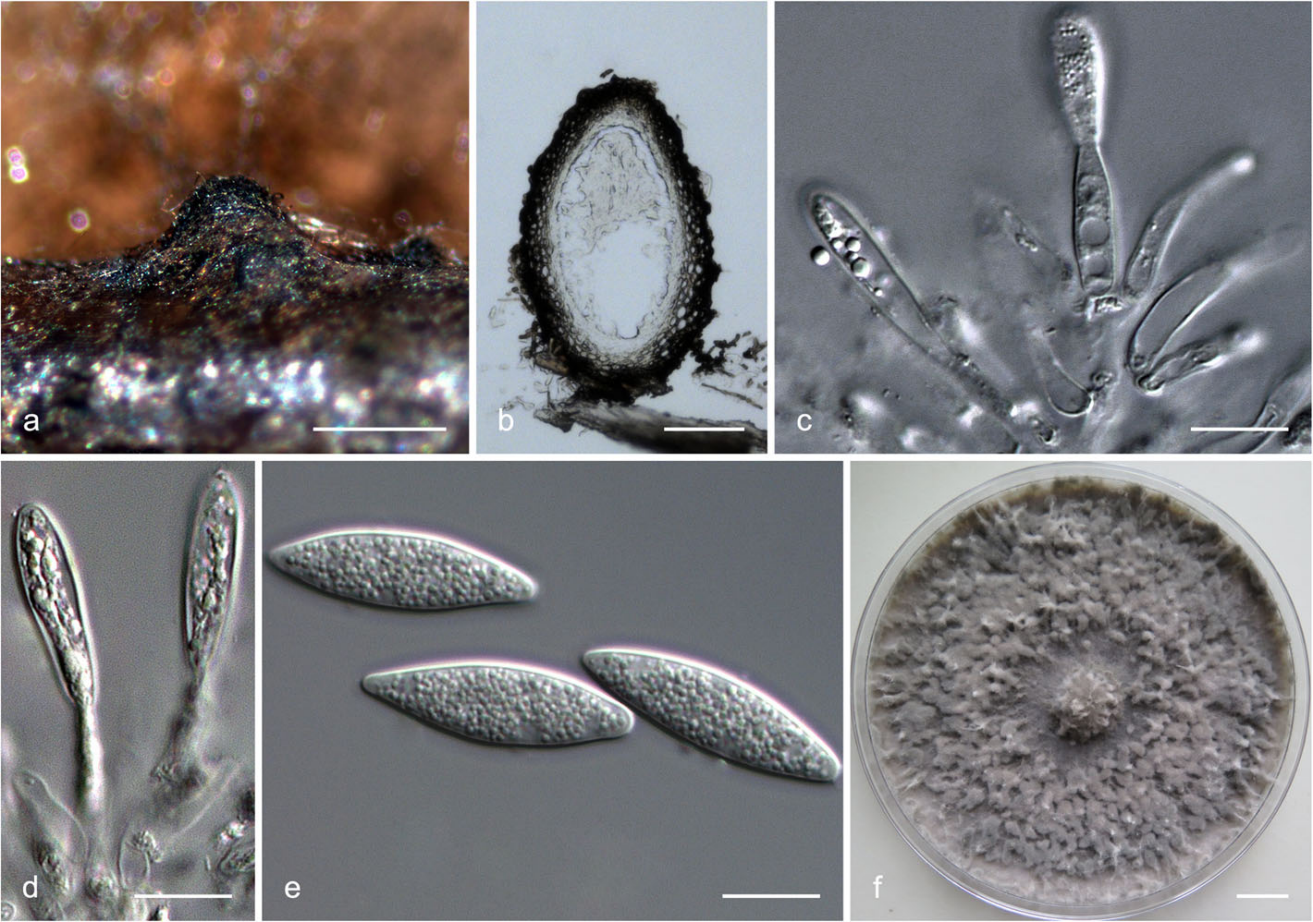
*Neofusicoccum magniconidium.* **a**. Conidiomata formed on pine needle culture; **b**. Longitudinal section through conidioma; **c**, **d**. Conidiogenous cells and developing conidia; **e**. Conidia; **f**. Living culture after 10 d on 2% MEA (front). Scale bars: a = 500 μm; b = 100 μm; c–e = 10 μm; f = 1 cm

MycoBank MB834104. (Fig. 7).

*Etymology*: Name refers to the exceptionally large conidia in this species.

*Diagnosis*: *Neofusicoccum magniconidium* is phylogenetically closely related to *N. ningerense* and *N. macroclavatum*. Its conidia are smaller than those of *N. macroclavatum* and conidia have not been observed in *N. ningerense*. *Neofusicoccum magniconidium* grows optimally at 25 °C, which is different to *N. ningerense* that grows best at 30 °C.

*Type*: **China**: YunNan Province, HongHe Region, PingBian County (GPS 23°08′02″N, 103°32′29″E), from twigs of one *E. urophylla × E. grandis* tree, 14 November 2014, *S.F. Chen & G.Q. Li*, fruiting structures induced on needles of *Pinus* sp. on water agar (HMAS255722 – holotype, CSF5876 = CGMCC3.20077 – ex-type culture).

*Description*: *Sexual state* unknown*. Conidiomata* pycnidial, produced on pine needles on WA medium within 4–6 wk., globose to ovoid, dark brown to black, up to 1224 μm wide, 774 μm high, embedded in needle tissue, semi-immersed to superficial, unilocular, with a central ostiole. *Conidiophores* reduced to conidiogenous cells. *Conidiogenous cells* holoblastic, discrete, hyaline, cylindrical to lageniform, phialidic with periclinal thickening, (8.5–)10–14.5(− 16.5) × 2.5–3.5(− 4) μm. *Paraphyses* not seen. *Conidia* hyaline, thin-walled, smooth with granular contents, unicellular, aseptate narrowly fusiform, base subtruncate to bluntly rounded, (27–)27.5–30(− 34) × (5.5–)6–7.5(− 8) μm (av. of 100 conidia 29.1 × 6.7 μm; L/W = 4.3) (Table 5).

*Culture characteristics*: Colonies on MEA medium with fluffy mycelia, uneven margins and a few cottony aerial mycelia reaching to the lids of Petri plates, mycelial mat appressed, sparse to moderately dense. Colony mycelia initially white, becoming pale mouse grey (15”“‘d) to mouse grey (13”“‘i) at the surface and olivaceous grey (23”“‘b) to iron grey (23”“‘k) at the reverse after 10 d. Optimal growth temperature 25 °C. No growth at 5 °C and 40 °C. After 4 d, colonies at 10 °C, 15 °C, 20 °C, 25 °C, 30 °C and 35 °C reaching 22 mm, 50 mm, 68 mm, 87 mm, 82 mm and 11 mm, respectively.

*Host*: *E. urophylla* × *E. grandis.*

*Distribution*: Currently known only from HongHe Region in YunNan Province, China.

Notes — *Neofusicoccum magniconidium* is phylogenetically closely related to *N. ningerense* and *N. macroclavatum*, but conidia (Table 5) of *N. magniconidium* (av. 29.1 × 6.7; L/W = 4.3) are smaller than those of *N. macroclavatum* (av. 30.3 × 7.1, L/W = 4.2; Burgess et al. 2005). *Neofusicoccum ningerense* could not be induced to sporulate in culture. Conidia of *N. macroclavatum* are occasionally 1–4-septate when mature before germination, and spermatia have been observed in this species (Burgess et al. 2005); characters not observed in *N. magniconidium*.

*Additional specimens examined*: **China**: YunNan Province, HongHe Region, PingBian County (GPS 23°08′02″N, 103°32′29″E), from twigs on one *E. urophylla × E. grandis* tree, 14 November 2014, *S.F. Chen & G.Q. Li*, fruiting structures induced on needles of *Pinus* sp. on water agar (HMAS255723, culture CSF5875 = CGMCC3.20076).

***Neofusicoccum ningerense*** G.Q. Li & S.F. Chen, **sp. nov*.***

**Fig. 8:**
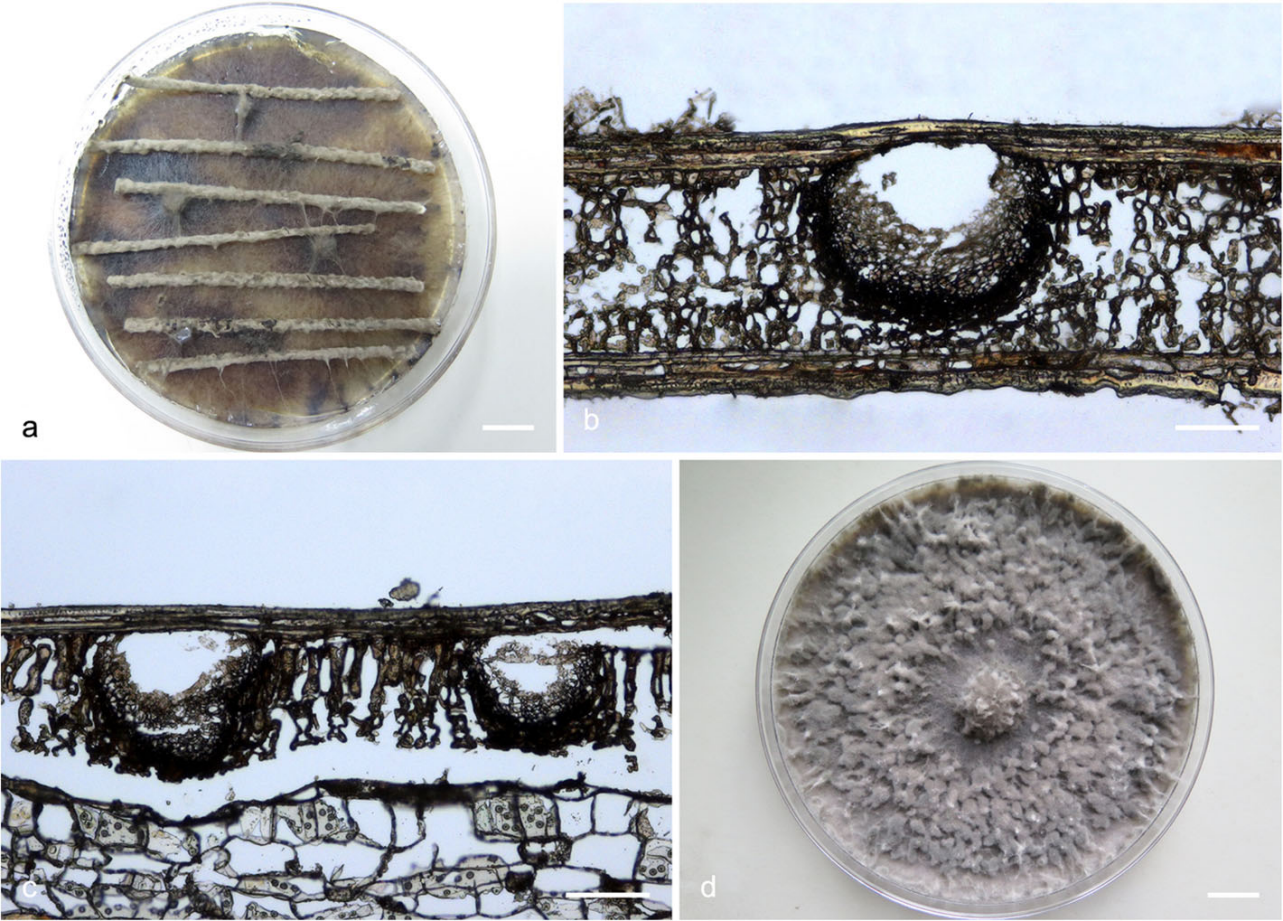
*Neofusicoccum ningerense.* **a**. WA plate with pine needle to induce sporulation; **b**, **c**. Longitudinal section through conidiomata-like structure; **d**. Living culture after 10 d on 2% MEA (front). Scale bars: a, d = 1 cm; b, c = 100 μm

MycoBank MB834105. (Fig. 8).

*Etymology*: Name refers to the NingEr County where the fungus was isolated for the first time.

*Diagnosis*: *Neofusicoccum ningerense* is closely related to *N. magniconidium,* but differs from the latter species at two bases in each of the ITS, *tub2* and *rpb2* loci. The optimal growth temperature for *N. ningerense* is also different from that of *N. magniconidium*.

*Type*: **China**: YunNan Province, PuEr Region, NingEr County (GPS 23°05′26″N, 102°02′40″E), from twigs of one *E. urophylla* × *E. grandis* tree, 16 November 2014, *S.F. Chen & G.Q. Li*, dried 30-day-old culture grown on 2% MEA at 25 °C (HMAS255724 – holotype, CSF6028 = CGMCC3.20078 – ex-type culture).

*Description*: *Sexual state* unknown*.* Conidiomata-like structures produced on pine needles on WA medium within 4–6 wk., embedded in needle tissue, unilocular (Fig. 8a–c). No conidiophores, conidiogenous cells or conidia have been observed.

*Culture characteristics*: Colonies on MEA medium with fluffy mycelia, uneven margins and a few cottony aerial mycelia reaching to the lids of Petri plates, mycelial mat appressed, sparse to moderately dense. Colony mycelia initially white, becoming pale mouse grey (15”“‘d) to mouse grey (13”“‘i) at the surface and olivaceous grey (23”“‘b) to iron grey (23”“‘k) at the reverse after 10 d. Optimal growth temperature is 30 °C, covering the 90 mm plates after 4 d. No growth at 5 °C and 40 °C. After 4 d, colonies at 10 °C, 15 °C, 20 °C, 25 °C, 30 °C and 35 °C reached 23 mm, 53 mm, 69 mm, 88 mm, 90 mm and 10 mm, respectively.

*Host*: *E. urophylla* × *E. grandis*.

*Distribution*: Currently known only from the PuEr Region in YunNan Province, China.

*Notes*: Only conidiomata were observed in this fungus, and no other asexual structures were observed. Different methods were used in an attempt to induce sporulation but all of these failed. *Neofusicoccum ningerense* is phylogenetically closely related to *N. magniconidium* (Fig. 4). The optimal growth temperature of *N. ningerense* (30 °C) differs from that of *N. magniconidium* (25 °C).

*Additional specimens examined*: **China**: YunNan Province, PuEr Region, NingEr County (GPS 23°05′26″N, 102°02′40″E), 16 November 2014, S.F. *Chen & G.Q. Li*, from twigs of one *E. urophylla* × *E. grandis* tree, dried 30-day-old culture grown on 2% MEA at 25 °C (HMAS255725, culture CSF6030 = CGMCC3.20079).

***Neofusicoccum parviconidium*** G.Q. Li & S.F. Chen, **sp. nov.**

**Fig. 9:**
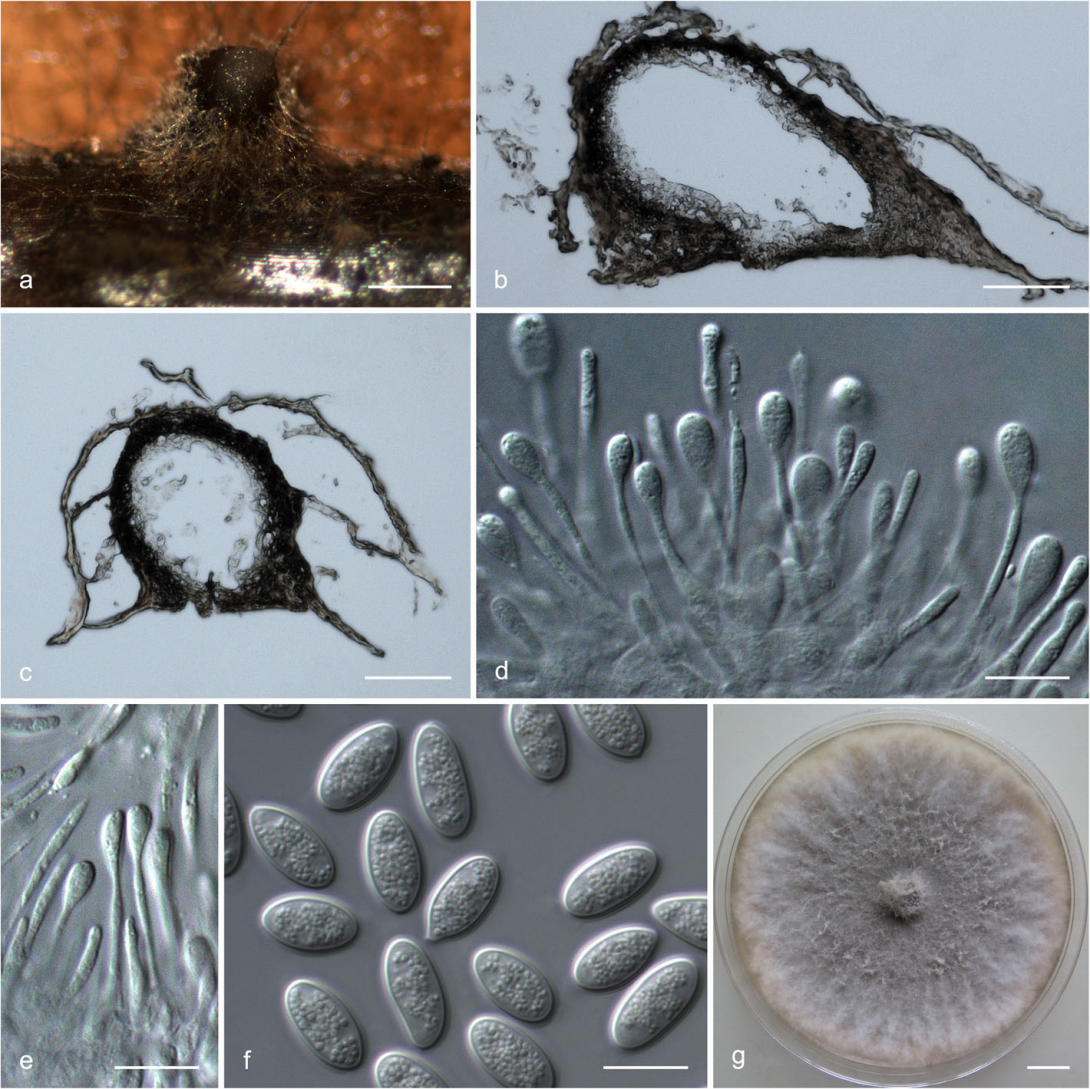
*Neofusicoccum parviconidium.* **a**. Conidioma formed on pine needle culture; **b**, **c**. Longitudinal section through conidioma; **d**, **e**. Conidiogenous cells and developing conidia; **f**. Conidia; **g**. Living culture after 10 d on 2% MEA (front). Scale bars: a = 500 μm; b, c = 100 μm; d–f = 10 μm; g = 1 cm

MycoBank MB834106. (Fig. 9).

*Etymology*: Name refers to the small conidia in this fungus*.*

*Diagnosis*: *Neofusicoccum parviconidium* can be distinguished from other *Neofusicoccum* species by its exceptionally short conidia.

*Type*: **China**: YunNan Province, HongHe Region, PingBian County (GPS 23°00′52″N, 103°38′09″E), from twigs of one *Eucalyptus* tree, 13 November 2014, *S.F. Chen & G.Q. Li*, fruiting structures induced on needles of *Pinus* sp. on water agar (HMAS255726 – holotype, CSF5667 = CGMCC3.20074 – ex-type culture).

*Description*: *Sexual state* unknown*. Conidiomata* pycnidial, produced on pine needles on WA medium within 4–6 wk., globose to ovoid, dark brown to black, up to 604 μm wide, 1205 μm high, embedded in needle tissue, semi-immersed to superficial, unilocular, with a central ostiole. *Conidiophores* reduced to conidiogenous cells. *Conidiogenous cells* holoblastic, discrete, hyaline, cylindrical to lageniform, phialidic with periclinal thickening, (5.5–)7–15(− 20) × 2–2.5(− 3) μm. *Paraphyses* not seen. *Conidia* hyaline, thin-walled, smooth with granular contents, unicellular, aseptate ellipsoid to fusoid, base subtruncate to bluntly rounded, (9.5–)10.5–11.5(− 12.5) × (4.4–)5–5.5(− 6) μm (av. of 100 conidia 10.9 × 5.2 μm; L/W = 2.1) (Table 5).

*Culture characteristics*: Colonies on MEA medium with fluffy mycelia, uneven margins and a few cottony aerial mycelia reaching to the lids of Petri plates, mycelial mat appressed, sparse to moderately dense. Colony mycelia initially white, becoming smoke grey (21”“f) to pale mouse grey (15”“‘d) at the surface and olivaceous (21”k) to iron grey (23″“‘k) at the reverse after 10 d. Optimal growth temperature 30 °C. No growth at 5 °C and 40 °C. After 4 d, colonies at 10 °C, 15 °C, 20 °C, 25 °C, 30 °C and 35 °C reaching 16 mm, 39 mm, 55 mm, 74 mm, 85 mm and 29 mm, respectively.

*Host*: *Eucalyptus* sp.

*Distribution*: Currently only known from HongHe Region in YunNan Province, China.

Notes: *Neofusicoccum parviconidium* is phylogenetically closely related to *N. mangiferae* and *N. microconidium* (Fig. 4), but conidia (Table 5) of *N. parviconidium* (av. 10.9 × 5.2; L/W = 2.1) are smaller than those of *N. mangiferae* (av. 13.6 × 5.4; L/W = 2.0–2.5; Slippers et al. 2005), shorter and wider than those of *N. microconidium* (av. 12.3 × 5.0; L/W = 2.5; Li et al. 2018).

*Additional specimens examined*: **China**: YunNan Province, HongHe Region, PingBian County (GPS 23°00′52″N, 103°38′09″E), from twigs on one *Eucalyptus* tree, 13 November 2014, *S.F. Chen & G.Q. Li*, fruiting structures induced on needles of *Pinus* sp. on water agar (HMAS255727, culture CSF5677 = CGMCC3.20085); YunNan Province, HongHe Region, PingBian County (GPS 23°00′52″N, 103°38′09″E), from twigs of one *Eucalyptus* tree, 13 November 2014, *S.F. Chen* & *G.Q. Li* (culture CSF5670); YunNan Province, HongHe Region, PingBian County (GPS 23°00′52″N, 103°38′09″E), from twigs of one *Eucalyptus* tree, 13 November 2014, *S.F. Chen* & *G.Q. Li* (culture CSF5681).

***Neofusicoccum yunnanense*** G.Q. Li & S.F. Chen, **sp. nov.**

**Fig. 10:**
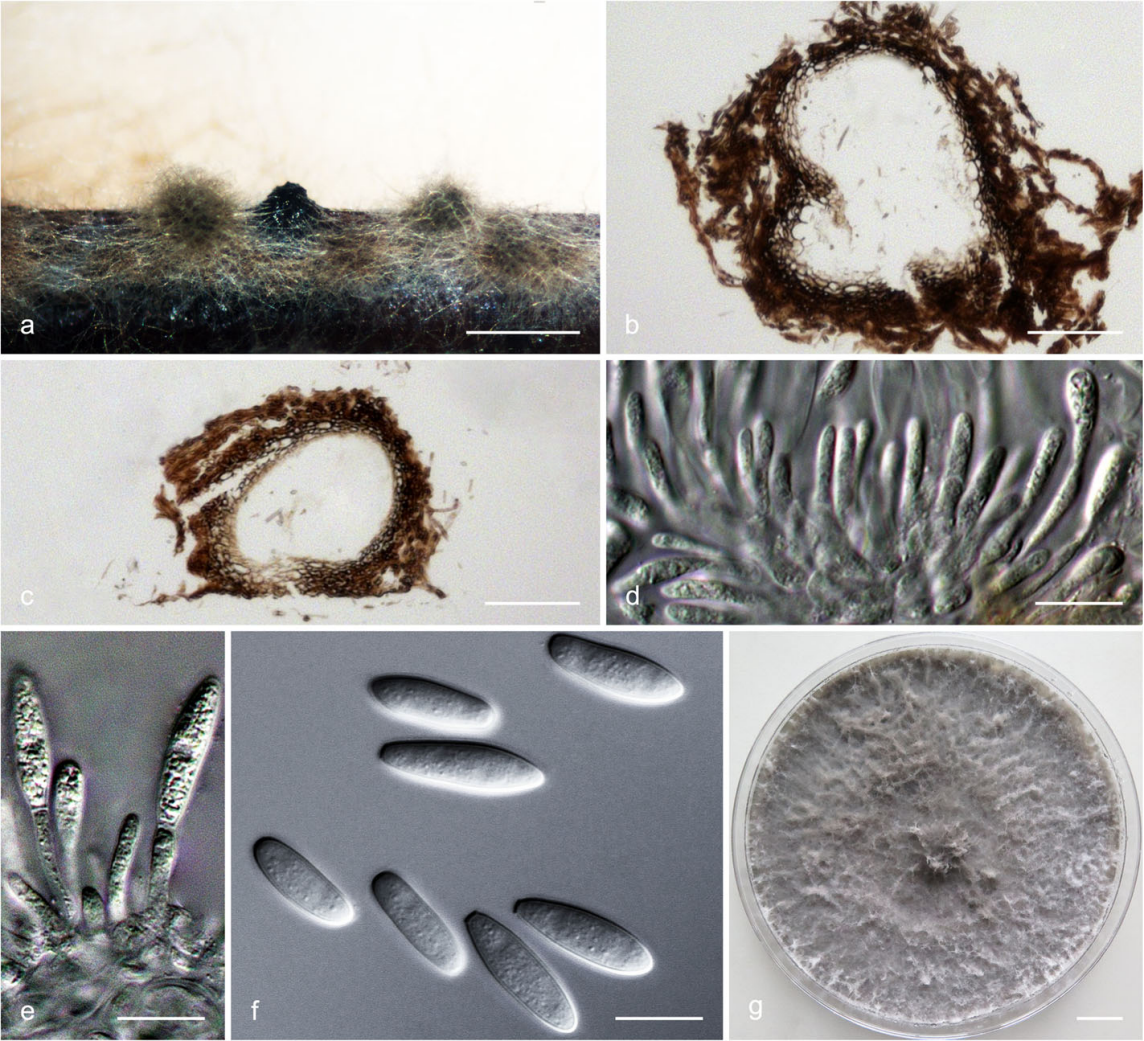
*Neofusicoccum yunnanense.* **a**. Conidiomata formed on pine needle culture; **b**, **c**. Longitudinal section through conidioma; **d**, **e**. Conidiogenous cells and developing conidia; **f**. Conidia; **g**. Living culture after 10 d on 2% MEA (front). Scale bars: a = 500 μm; b, c = 100 μm; d–f = 10 μm; g = 1 cm

MycoBank MB834107. (Fig. 10).

*Etymology*: Name refers to the YunNan Province where the fungus was isolated for the first time.

*Diagnosis*: *Neofusicoccum yunnanense* resides in ‘*N. parvum* / *N. ribis*’ complex and has smaller conidia than its closest relatives, *N. algeriense*, *N. dianense*, *N. italium* and *N. parvum*, yet longer than those of *N. hongkongense*. *Neofusicoccum yunnanense* grew optimally at 30 °C, which is different from that of *N. algeriense* (25 °C), *N. dianense* (25 °C) and *N. hongkongense* (25 °C). Data for growth in culture are not available for *N. italium* or *N. parvum*.

*Type*: **China**: YunNan Province, ChuXiong Region, LuFeng County (GPS 25°03′12″N, 101°46′29″E), from twigs of one *E. globulus* tree, 19 November 2014, *S.F. Chen & G.Q. Li*, fruiting structures induced on needles of *Pinus* sp. on water agar (HMAS255728 – holotype, CSF6142 = CGMCC3.20083 – ex-type culture).

*Description*: *Sexual state* unknown*. Conidiomata* pycnidial, produced on pine needles on WA medium within 4–6 wk., globose to ovoid, dark brown to black, up to 982 μm wide, 549 μm high, embedded in needle tissue, semi-immersed to superficial, unilocular, with a central ostiole. *Conidiophores* reduced to conidiogenous cells. *Conidiogenous cells* holoblastic, discrete, hyaline, cylindrical to lageniform, phialidic with periclinal thickening, (10.5–)11–15(− 18.5) × (1.5–)2–2.5(− 3) μm. *Paraphyses* not seen. *Conidia* hyaline, thin-walled, smooth with granular contents, unicellular, aseptate narrowly fusiform, base subtruncate to bluntly rounded, (13–)13.5–17.5(− 20) × (3.5–)4–4.5(− 5) μm (av. of 100 conidia 15.6 × 4.4 μm; L/W = 3.5) (Table 5).

*Culture characteristics*: Colonies on MEA medium with fluffy mycelia, uneven margins and a few cottony aerial mycelia reaching the lids of Petri plates, mycelial mats appressed and sparse to moderately dense. Colony mycelia initially white, becoming pale mouse grey (15”“‘d) to mouse grey (13”“‘i) at the surface and olivaceous grey (23”“‘b) to iron grey (23”“‘k) at the reverse after 10 d. Optimal growth temperature 30 °C, covering the 90 mm plates after 4 d. No growth at 5 °C and 40 °C. After 4 d, colonies at 10 °C, 15 °C, 20 °C, 25 °C, 30 °C and 35 °C reaching 13 mm, 42 mm, 64 mm, 86 mm, 90 mm and 16 mm, respectively.

*Host*: *E. globulus*, *E. urophylla × E. grandis* and *Eucalyptus* sp.

*Distribution*: Currently known from ChuXiong, HongHe, KunMing, PuEr, WenShan and YuXi Regions in YunNan Province, China.

*Notes*: *Neofusicoccum yunnanense* is phylogenetically closely related to *N. algeriense*, *N. dianense*, *N. hongkongense*, *N. italium* and *N. parvum* (Fig. 4). Conidia of *N. yunnanense* (av. 15.6 × 4.4; L/W = 3.5) are smaller than those of *N. algeriense* (av. 17.6 × 5.6; L/W = 3.1; Berraf-Tebbal et al. 2014), *N. dianense* (av. 18.9 × 5.2; L/W = 3.6), *N. italium* (av. 15.8 × 5.2; L/W = 3.0; Marin-Felix et al. 2017) and *N. parvum* (av. 17.1 × 5.5; L/W = 3.2; Phillips et al. 2013) and longer than those of *N. hongkongense* (av. 14.1 × 4.7; L/W = 3.0; Li et al. 2018).

*Additional specimens examined*: **China**: YunNan Province, PuEr Region, NingEr County (GPS 23°05′26″N, 102°02′40″E), from twigs of one *E. urophylla* × *E. grandis* tree, 16 November 2014, *S.F. Chen & G.Q. Li*, fruiting structures induced on needles of *Pinus* sp. on water agar (HMAS255729, culture CSF6034 = CGMCC3.20080); YunNan Province, HongHe Region, PingBian County (GPS 23°04′02″N, 103°36′33″E), from twigs of one *Eucalyptus* tree, 13 November 2014, *S.F. Chen* & *G.Q. Li* (culture CSF5686); YunNan Province, KunMing Region, AnNing County (GPS 24°55′02″N, 102°23′41″E), from twigs of one *E. globulus* tree, 19 November 2014, *S.F. Chen* & *G.Q. Li* (culture CSF6169).

### Distribution of *Botryosphaeriaceae* in YunNan Province

**Fig. 11:**
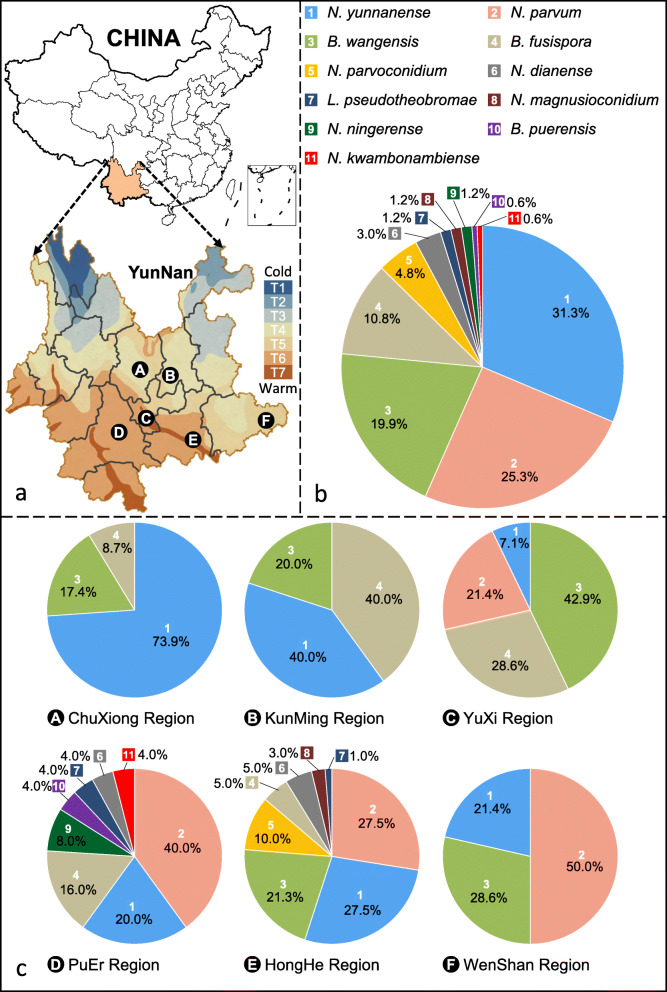
*Botryosphaeriaceae* species detected from *Eucalyptus* plantations in six regions in YunNan Province. **a**. Sampling regions across different climatic zones. T1: cold highland zone, T2: central temperate zone, T3: southern temperate zone, T4: northern sub-tropical zone, T5: central sub-tropical zone, T6: southern sub-tropical zone, T7: tropical zone; **b**. Prevalence of *Botryosphaeriaceae* species as a percentage of the total isolates in YunNan Province. Different species are represented by numbers with different colours; **c**. Prevalence of *Botryosphaeriaceae* species as a percentage of the total isolates in each of the different sampling regions

Based on phylogenetic and morphological analyses, eleven species were identified from collections in YunNan Province. Of these, *Neofusicoccum yunnanense* (31.3%) was the most prevalent species, followed by *N. parvum* (25.3%), *B. wangensis* (19.9%), *B. fusispora* (10.8%), *N. parviconidium* (4.8%), *N. dianense* (3.0%), *L. pseudotheobromae* (1.2%), *N. magniconidium* (1.2%), *N. ningerense* (1.2%), *B. puerensis* (0.6%) and *N. kwambonambiense* (0.6%) (Fig. 11b). *Neofusicoccum yunnanense* was detected in all six regions surveyed, *B. wangensis* was found in all regions other than PuEr, *N. parvum* was found in all regions other than ChuXiong, *B. fusispora* was found in the ChuXiong, HongHe, PuEr and YuXi Regions, and the other species were found in one or two regions of YunNan (Fig. 11c).

Sampling sites in this study included four distinct climate types. Samples in ChuXiong (Region A), KunMing (Region B) and WenShan (Region F) Regions were from the northern sub-tropical or central sub-tropical zone; samples in HongHe (Region E), PuEr (Region D) and YuXi (Region C) were from the southern sub-tropical or tropical zone. Four species were detected in all four climate types surveyed and these included *B. fusispora*, *B. wangensis*, *N. parvum* and *N. yunnanense*. The remaining seven species identified in this study were detected in only southern sub-tropical or tropical zone (Fig. 11a, c).

### Pathogenicity tests

**Fig. 12:**
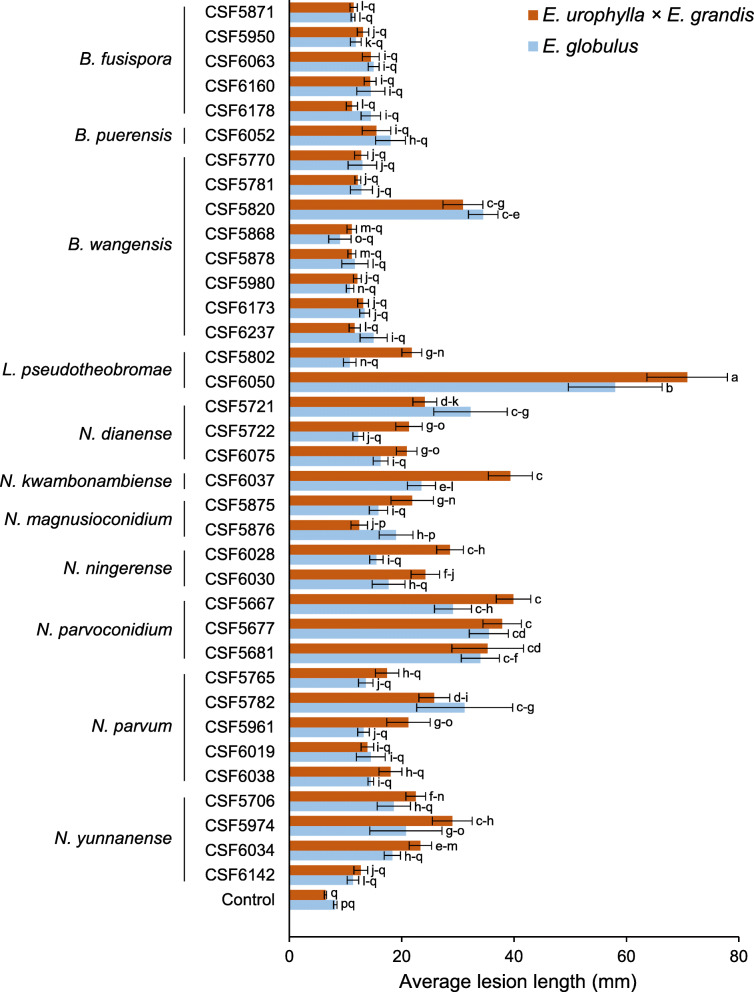
Column chart indicating the average lesion length (mm) produced by 36 isolates of *Botryosphaeriaceae* on tested plants of *E. globulus* and *E. urophylla × E. grandis*. Horizontal bars represent standard error of means. Different numbers on the right of bars indicate treatment means that are significantly different (*P* = 0.05)

Based on their ITS, *tef1* and *tub2* genotypes, thirty-six isolates of the *Botryosphaeriaceae* in three genera and representing 11 species were selected for inoculation. Typical lesions were observed on inoculated *Eucalyptus* plants and lesion lengths were recorded one month after inoculation. The results of pathogenicity tests showed that all isolates produced lesions on the test plants, while the controls produced only small zones of wound reaction (Fig. 12, Additional file 1: Figure S1). The inoculated species were re-isolated from the lesions, but never from the negative controls. Consequently, Koch’s postulates were fulfilled.

Lesion length data were not normally distributed based on Kolmogorov-Smirnov normality test (*P* < 0.05). All data were consequently transformed (Kolmogorov-Smirnov normality test, *P* = 0.2) by conducting a Rank transformation using the statistical package SPSS v. 20.

On *E. globulus* and *E. urophylla* × *E. grandis*, the shortest lesions were produced by isolate CSF5802 of *L. pseudotheobromae* and isolate CSF6178 of *B. fusispora* (Fig. 12). Results of the one-way ANOVA showed that some isolates produced lesions significantly longer than those caused by isolate CSF5802 on *E. globulus* and isolate CSF6178 on *E. urophylla* × *E. grandis* (*P* = 0.05). These isolates included CSF5820 (*B. wangensis*), CSF6050 (*L. pseudotheobromae*), CSF5721 and CSF6075 (*N. dianense*), CSF6037 (*N. kwambonambiense*), CSF5875 (*N. magniconidium*), CSF6028 and CSF6030 (*N. ningerense*), CSF5667, CSF5677 and CSF5681 (*N. parviconidium*), CSF5782 and CSF6038 (*N. parvum*), CSF5706, CSF5974 and CSF6034 (*N. yunnanense*) as shown in Fig. 12. Of these, the most aggressive isolate was CSF6050 (*L. pseudotheobromae*), which produced the longest lesions on *E. urophylla* × *E. grandis* (70.80 ± 7.17 mm) and *E. globulus* (58.00 ± 8.34 mm) as shown in Fig. 12.

Results of GLM Univariate Analysis (two-way ANOVA) showed a significant (*P* = 0.001) interaction effect between isolate and host. The analyses also showed that not all isolates of the same species of *Botryosphaeriaceae* reacted in the same manner on the tested *E. urophylla* × *E. grandis* clone or *E. globulus* plants. For example, lesions produced by isolate CSF5802 (*L. pseudotheobromae*) on *E. urophylla × E. grandis* were significantly longer than those on *E. globulus*, while the lesion lengths produced by isolate CSF6050 (*L. pseudotheobromae*) on the two tested *Eucalyptus* genotypes were not significantly different (*P* = 0.05). The results also showed that the pathogenicity of isolates of the same species on the two tested *Eucalyptus* genotypes can be different. For example, lesion lengths produced by isolate CSF5820 (*B. wangensis*) on *E. urophylla × E. grandis* and *E. globulus* were significantly longer than the other isolates of this species (*P* = 0.05) (Fig. 12). In contrast, lesion lengths produced by all isolates of *B. fusispora* on both *E. urophylla × E. grandis* and *E. globulus* were not significantly different (*P* = 0.05) from each other (Fig. 12).

For the tested isolates residing in three genera of the *Botryosphaeriaceae*, the overall data showed that species of *Lasiodiplodia* were the most aggressive, followed by those in *Neofusicoccum* (Fig. 12). The overall data also showed that plants of the *E. urophylla × E. grandis* clone and *E. globulus* seed-derived plants had similar levels of susceptibility to most of the tested isolates (Fig. 12). The exceptions were for isolates CSF5802 (*L. pseudotheobromae*), CSF5722 (*N. dianense*), CSF6028 (*N. ningerense*), and CSF5974 (*N. yunnanense*), where the lesions were significantly different on the *E. urophylla × E. grandis* clone and the *E. globulus* plants.

## DISCUSSION

In this study, 166 isolates of the *Botryosphaeriaceae* were characterized from *Eucalyptus* plantations in six regions of the YunNan Province. Eleven species residing in the three genera *Botryosphaeria*, *Lasiodiplodia* and *Neofusicoccum* were identified. These included *Botryosphaeria fusispora*, *B. wangensis*, *Lasiodiplodia pseudotheobromae*, *Neofusicoccum kwambonambiense*, *N. parvum*, and six novel species described here as *B. puerensis*, *N. dianense*, *N. magniconidium*, *N. ningerense*, *N. parviconidium* and *N. yunnanense*.

Analysis of multi-gene phylogenetic concordance has emerged as standard practice for species identification in the *Botryosphaeriaceae* (Phillips et al. 2013; Chen et al. 2014a, 2014b; Slippers et al. 2017; Yang et al. 2017; Li et al. 2018; Jayawardena et al. 2019a, 2019b; Phillips et al. 2019). This approach was also essential in the present study to distinguish between closely related species, where we considered the phylogenetic signal for four loci, including ITS, *tef1*, *tub2* and *rpb2*. The most common loci used for species delineation in *Botryosphaeria* are ITS, *tef1* and *tub2* (Phillips et al. 2013; Chen et al. 2014a, 2014b; Osorio et al. 2017; Li et al. 2018) and in *Lasiodiplodia* and *Neofusicoccum* are ITS, *tef1*, *tub2* and *rpb2* (Pavlic et al. 2009a, 2009b; Sakalidis et al. 2011; Cruywagen et al. 2017; Yang et al. 2017; Li et al. 2018; Phillips et al. 2019). These were also the most informative loci for the genera in this study. However, a limitation lies in the fact that there are numerous species for which sequence data are not available for all of these loci.

The majority of the isolates (67%) obtained in this study were species of *Neofusicoccum.* Five of these were previously undescribed taxa and these were found in addition to the well-known species *N. kwambonambiense* and *N. parvum*. Together with the newly described species, *Neofusicoccum* now includes 48 species (Phillips et al. 2013; Yang et al. 2017; Jami et al. 2018; Li et al. 2018).

*Neofusicoccum yunnanense* was isolated from all six regions in the sub-tropical and tropical zones, suggesting that it has a wide distribution in different climatic zones. In contrast, the other new species of *Neofusicoccum* (*N. dianense*, *N. magniconidium*, *N. ningerense* and *N. parviconidium*) were all from the southern sub-tropical or tropical zone that has relatively high average temperatures. *Neofusicoccum parvum* was isolated in five sampled regions, while *N. kwambonambiense* was isolated only from PuEr. A previous study has shown that these two species have a wide geographic distribution including areas, with mediterranean and sub-tropical climates worldwide (Sakalidis et al. 2013), and that they have a wide range of hosts (Pavlic et al. 2009a; Phillips et al. 2013; Sakalidis et al. 2013). In China, *N. parvum* has also been reported from a wide range of hosts including *Cupressus funebris* (Li et al. 2010), *Eriobotrya japonica* (Zhai and Zhang 2019), *Eucalyptus* spp. (Chen et al. 2011), *Koelreuteria paniculata* (Fang et al. 2019), *Hevea brasiliensis* (Liu et al. 2017) and *Juglans regia* (Yu et al. 2015) and in these cases, from sub-tropical and tropical zones. *Neofusicoccum kwambonambiense* was first reported from *Syzygium cordatum* (Myrtaceae) in South Africa (Pavlic et al. 2009a). The present study represents the first report of this species associated with *Eucalyptus* and also the *Myrtaceae* in China.

Two new cryptic species (*N. dianense* and *N. yunnanense*) were discovered in the ‘*N. parvum* / *N. ribis*’ complex based on concordance in the phylogenetic analyses of the ITS, *tef1*, *tub2* and *rpb2* datasets in this study. Cryptic species are defined as two or more distinct species often treated as a single species because they are at least superficially indistinguishable based on their morphology (Bickford et al. 2007). The use of multi-locus phylogenetic concordance has revealed numerous cryptic species in the *Botryosphaeriaceae* in recent years (Alves et al. 2008; Pavlic et al. 2009b; Phillips et al. 2013; Slippers et al. 2014, 2017; Yang et al. 2017). This is especially true in the ‘*N. parvum* / *N. ribis*’ complex, where six cryptic species with similar conidia have been distinguished based on multigene analyses (Pavlic et al. 2009a; Sakalidis et al. 2011; Li et al. 2018). Amongst the three new *Neofusicoccum* species (*N. magniconidium*, *N. ningerense* and *N. parviconidium*) discovered in the present study and that reside in the ‘*N. parvum* / *N. ribis*’ complex, *N. parviconidium*, like *N. microconidium*, have relatively small conidia compared to other species in the genus. *Neofusicoccum magniconidium* has larger conidia in comparison with those of *N. macroclavatum*, and it is phylogenetically most closely related to *N. macroclavatum*, and *N. ningerense*, the latter of which failed to produce fruiting structures. These newly described species, together with other species in the ‘*N. parvum* / *N. ribis*’ complex, makes this one of the most widespread ‘lineages’ in the *Botryosphaeriaceae*.

When our results are consolidated with those from previous studies (Chen et al. 2011; Li et al. 2018), a total of nine species of *Neofusicoccum* have been identified from *Eucalyptus* plantations in China. These include *N. dianense*, *N. kwambonambiense*, *N. magniconidium*, *N. microconidium*, *N. ningerense*, *N. parviconidium*, *N. parvum*, *N. sinoeucalypti* and *N. yunnanense*. Seven of these nine species were first described from or are known only from China on *Eucalyptus* in plantations. The exceptions are *N. parvum* and *N. kwambonambiense* (Chen et al. 2011, Li et al. 2018). These results suggest an unusually high diversity of *Neofusicoccum* species in non-native *Eucalyptus* plantations in China. They could also imply that many additional *Neofusicoccum* species could exist in yet unsampled regions of the country.

A total of 52 isolates were identified as species of *Botryosphaeria*, including *B. fusispora*, *B. wangensis* and the newly described *B. puerensis* found in this study. The genus *Botryosphaeria* was first introduced in 1863 by Cesati & De Notaris, and 143 species were recorded in this genus up to 1997 (Denman et al. 2000). As is true for most groups in the *Botryosphaeriaceae*, *Botryosphaeria* has been substantially revised in recent years using a combination of DNA sequence and morphological data. The genus now accommodates 16 species for which clear taxonomic descriptions and DNA sequence data are available (Phillips et al. 2013; Slippers et al. 2014; Xu et al. 2015; Ariyawansa et al. 2016; Zhou et al. 2016, 2017; Li et al. 2018).

Many *Botryosphaeria* species occur widespread across a broad climatic environment and on diverse hosts. For example, *Botryosphaeria fusispora* was first described from *Entada* sp. in Thailand (Chiang Rai, Doi Tung: tropical zone; Liu et al. 2012), and subsequently in the FuJian, GuangDong and GuangXi Provinces in sub-tropical and tropical zones in China (Li et al. 2018). In the present study, *B. fusispora* was isolated in four of six sampled regions in the YunNan Province, indicating that this species has a wide distribution in *Eucalyptus* plantations in sub-tropical and tropical zones. *Botryosphaeria wangensis* was known only from *Cedrus deodara* in the HeNan Province in Central China (temperate zone) previously (Li et al. 2018). In contrast, it was detected in five regions (sub-tropical and tropical zones) in YunNan Province in the present study, suggesting that it can also survive at a broad range of temperatures. Many of the other *Botryosphaeria* species previously described occur in more temperate climates, but this is clearly not a characteristic of the genus.

The newly described *B. puerensis* is known from only one isolate. It was clearly separate from all other known species based on phylogenetic analyses of *tef1*, *tub2* and *rpb2* datasets. Obvious morphological differences were also observed between *B. puerensis* and its closest known sister species. While we recognise that it is preferable to describe new species based on more than one isolate or specimen (Seifert and Rossman 2010), we chose to describe this species because it was well defined and this is not unprecedented in studies of the *Botryosphaeriaceae* (e.g. Slippers et al. 2014; Yang et al. 2017; Zhang et al. 2017).

*Lasiodiplodia pseudotheobromae* was identified from *Eucalyptus* plantations in PuEr and HongHe Regions (tropical zone) in YunNan Province. This species has previously been reported from a wide variety of hosts across many different climate zones globally including Brazil (tropical zone) (Netto et al. 2014), China (sub-tropical and tropical zones) (Zhao et al. 2010; Li et al. 2018), Costa Rica and Suriname (tropical zone) (Alves et al. 2008), amongst many others. In China, *L. pseudotheobromae* was first reported in 2010 (Zhao et al. 2010) and recorded from different plant species more recently (Chen et al. 2011; Dissanayake et al. 2015; Li et al. 2015; Tennakoon et al. 2016; Wu et al. 2019). Collectively, these results suggest that *L. pseudotheobromae* is one of the most widespread species in the *Botryosphaeriaceae* globally and it has at least 105 recorded hosts (NCBI Nucleotide Database, 2019). It is a species that might easily be spread amongst regions and can be expected to have an important impact on a wide variety of plant-based industries in a diversity of environments.

Overall, the results of this study suggest that climate influences the distribution of *Botryosphaeriaceae*, even over relatively small distances (560 km across the widest sampling points in this study). This is despite the obvious adaptability to both hosts and temperature ranges that is reflected in their wide geographic distribution across climates worldwide (Slippers and Wingfield 2007; Slippers et al. 2014). Only three species of *Botryosphaeria* and one species of *Lasiodiplodia* were detected in the sub-tropical or tropical zone in YunNan Province, compared to the seven species of *Neofusicoccum*. A greater number of *Botryosphaeriaceae* species were detected in the southern sub-tropical or tropical zone (PuEr and HongHe Regions) than northern sub-tropical or central sub-tropical zone (ChuXiong, KunMing and WenShan Regions), suggesting that climate affects the distribution of species in the Botryosphaeiraceae. Relatively few species were detected from YuXi Region in the sub-tropical or tropical zone, which might have been affected by the lower number of samples collected in this region. Factors that probably affect this species diversity and distribution include climates such as temperature and water, host-associated factors such as species and age of host and the host structures from which isolations are made (Slippers et al. 2017; Velásquez et al. 2018).

All 11 species identified in this study were pathogenic to the *E. urophylla* × *E. grandis* hybrid clone and *E. globulus* seed-derived plants. Some of these species could present threats to the *Eucalyptus* industry. One isolate of *L. pseudotheobromae* produced significantly longer lesions than those of other genera of *Botryosphaeriaceae* on the tested *Eucalyptus* genotypes, which is consistent with the results of previous studies (Pérez et al. 2010; Chen et al. 2011; Li et al. 2018). With the exception of one isolate, isolates of the *Botryosphaeria* spp. produced the smallest lesions in the pathogenicity tests; a result similar to that of previous studies (Li et al. 2018). The species of *Neofusicoccum* were also pathogenic and produced lesions that were generally larger than those associated with the *Botryosphaeria* species, which is also consistent with the results of previous studies (Mohali et al. 2009; Pérez et al. 2010; Chen et al. 2011; Li et al. 2018). There was also significant variation in aggressiveness between isolates of species, which emphasises that evaluation of pathogenicity linked to *Eucalyptus* breeding trials should include isolates covering a broad range of aggressiveness.

The present study provides foundational data on the diversity, distribution and pathogenicity of the *Botryosphaeriaceae* from *Eucalyptus* plantations in YunNan Province in southwestern China. Together with previous studies (Chen et al. 2011; Li et al. 2015, 2018), the results revealed a high level of *Botryosphaeriaceae* diversity associated with diseased *Eucalyptus* in the sampled plantations. Special attention should be afforded in future monitoring, to species with wide distributions and high levels of aggressiveness to species of *Eucalyptus*.

## CONCLUSIONS

This study provides important new data regarding on the diversity, distribution and pathogenicity of the *Botryosphaeriaceae* from *Eucalyptus* plantations in YunNan Province in southwestern China. Results revealed a high level of *Botryosphaeriaceae* diversity associated with diseased *Eucalyptus* in the sampled plantations. Species diversity and composition changed across the different climatic zones, despite their relatively close proximity and the fact that some of the species have a global distribution. All the *Botryosphaeriaceae* species were pathogenic to tested one-year-old *Eucalyptus* plants, but showed significant inter- and intra-species variation in aggressiveness amongst isolates. Future tree disease monitoring should consider *Botryosphaeriaceae* species with wide distributions and high levels of aggressiveness to species of *Eucalyptus*. The study also provides a foundation for monitoring and management of *Botryosphaeriaceae* through selection and breeding of *Eucalyptus* in the YunNan Province in southwestern China.

## Supplementary information

**Additional file 1: Figure S1.** Symptoms observed on *E. globulus* and *E. urophylla* × *E. grandis* one month after inoculation. **a, b**. lesion produced on *E. globulus* by isolates (a) CSF6050 (*L. pseudotheobromae*) and (b) CSF5667 (*N. parviconidium*); **c**. negative control showing the absence of lesion development on *E. globulus*; **d–k**. lesion produced on *E. urophylla* × *E. grandis* by isolates (d) CSF5871 (*B. fusispora*), (e) CSF5820 (*B. wangensis*), (f) CSF5721 (*N. dianense*), (g) CSF5876 (*N. magniconidium*), (h) CSF6028 (*N. ningerense*), (i) CSF5667 (*N. parviconidium*), (j) CSF5782 (*N. parvum*), and (k) CSF5974 (*N. yunnanense*); **l**. negative control showing the absence of lesion development on *E. urophylla* × *E. grandis*.

## Data Availability

All data generated or analysed during this study are included in this published article [and its supplementary information files].

## References

[CR1] Abdollahzadeh J, Javadi A, Goltapeh EM, Zare R, Phillips AJL (2010). Phylogeny and morphology of four new species of *Lasiodiplodia* from Iran. Persoonia.

[CR2] Abdollahzadeh J, Zare R, Phillips AJL (2013). Phylogeny and taxonomy of *Botryosphaeria* and *Neofusicoccum* species in Iran, with description of *Botryosphaeria scharifii* sp. nov. Mycologia.

[CR3] Alves A, Correia A, Luque J, Phillips A (2004). *Botryosphaeria corticola*, sp. nov. on *Quercus* species, with notes and description of *Botryosphaeria stevensii* and its anamorph, *Diplodia mutila*. Mycologia.

[CR4] Alves A, Crous PW, Correia A, Phillips AJL (2008). Morphological and molecular data reveal cryptic speciation in *Lasiodiplodia theobromae*. Fungal Diversity.

[CR5] Ariyawansa HA, Hyde KD, Liu JK, Wu S-P, Liu ZY (2016). Additions to karst Fungi 1: *Botryosphaeria minutispermatia* sp. nov., from Guizhou Province, China. Phytotaxa.

[CR6] Begoude BAD (2010). Characterization of *Botryosphaeriaceae* and *Cryphonectriaceae* associated with *Terminalia* spp. in Africa.

[CR7] Begoude BAD, Slippers B, Wingfield MJ, Roux J (2010). *Botryosphaeriaceae* associated with *Terminalia catappa* in Cameroon, South Africa and Madagascar. Mycological Progress.

[CR8] Berraf-Tebbal A, Guereiro MA, Phillips AJL (2014). Phylogeny of *Neofusicoccum* species associated with grapevine trunk disease in Algeria, with description of *Neofusicoccum algeriense* sp. nov. Phytopathologia Mediterranea.

[CR9] Bickford D, Lohman DJ, Sodhi NS, Ng PKL, Meier R, Winker K, Ingram KK, Das I (2007). Cryptic species as a window on diversity and conservation. Trends in Ecology & Evolution.

[CR10] Billones-Baaijens R, Savocchia S (2019). A review of *Botryosphaeriaceae* species associated with grapevine trunk diseases in Australia and New Zealand. Australasian Plant Pathology.

[CR11] Burgess TI, Andjic V, Hardy GESJ, Dell B, Xu D (2006). First report of *Phaeophleospora destructans* in China. Journal of Tropical Forest Science.

[CR12] Burgess TI, Barber PA, Hardy GESJ (2005). *Botryosphaeria* spp. associated with eucalypts in Western Australia, including the description of *Fusicoccum macroclavatum* sp. nov. Australasian Plant Pathology.

[CR13] Burgess TI, Barber PA, Mohali S, Pegg G, de Beer W, Wingfield MJ (2006). Three new *Lasiodiplodia* spp. from the tropics, recognized based on DNA sequence comparisons and morphology. Mycologia.

[CR14] Carstensen GD, Venter SN, Wingfield MJ, Coutinho TA (2017). Two *Ralstonia* species associated with bacterial wilt of *Eucalyptus*. Plant Pathology.

[CR15] Chen SF, Gryzenhout M, Roux J, Xie YJ, Wingfield MJ, Zhou XD (2010). Identification and pathogenicity of *Chrysoporthe cubensis* on *Eucalyptus* and *Syzygium* spp. in South China. Plant Disease.

[CR16] Chen SF, Li GQ, Liu FF, Michailides TJ (2015). Novel species of *Botryosphaeriaceae* associated with shoot blight of pistachio. Mycologia.

[CR17] Chen SF, Liu QL, Li GQ, Wingfield MJ (2017). *Quambalaria* species associated with eucalypt diseases in southern China. Frontiers of Agricultural Science and Engineering.

[CR18] Chen SF, Morgan DP, Hasey JK, Anderson K, Michailides TJ (2014). Phylogeny, morphology, distribution, and pathogenicity of *Botryosphaeriaceae* and *Diaporthaceae* from English walnut in California. Plant Disease.

[CR19] Chen SF, Morgan DP, Michailides TJ (2014). *Botryosphaeriaceae* and *Diaporthaceae* associated with panicle and shoot blight of pistachio in California, USA. Fungal Diversity.

[CR20] Chen SF, Pavlic D, Roux J, Slippers B, Xie YJ, Wingfield MJ, Zhou XD (2011). Characterization of *Botryosphaeriaceae* from plantation-grown *Eucalyptus* species in South China. Plant Pathology.

[CR21] Chen SF, van Wyk M, Roux J, Wingfield MJ, Xie YJ, Zhou XD (2013). Taxonomy and pathogenicity of *Ceratocystis* species on Eucalyptus trees in South China, including *C. chinaeucensis* sp. nov. Fungal Diversity.

[CR22] Coppen JJW (2002). *Eucalyptus*: the genus *Eucalyptus*.

[CR23] Coutinho IBL, Freire FCO, Lima CS, Lima JS, Gonçalves FJT, Machado AR, Silva AMS, Cardoso JE (2017). Diversity of genus *Lasiodiplodia* associated with perennial tropical fruit plants in northeastern Brazil. Plant Pathology.

[CR24] Crous PW, Groenewald JZ, Shivas RG, Edwards J, Seifert KA, Alfenas AC, Alfenas RF, Burgess TI, Carnegie AJ, Hardy GESJ, Hiscock N, Hüberli D, Jung T, Louis-Seize G, Okada G, Pereira OL, Stukely MJC, Wang W, White GP, Young AJ, McTaggart AR, Pascoe IG, Porter IJ, Quaedvlieg W (2011). Fungal planet description sheets: 69–91. Persoonia.

[CR25] Crous PW, Groenewald JZ, Wingfield MJ, Phillips AJL (2007). Neofusicoccum mediterraneum. Fungal planet 19.

[CR26] Crous PW, Wingfield MJ, Guarro J, Cheewangkoon R, van der Bank M, Swart WJ, Stchigel AM, Cano-Lira JF, Roux J, Madrid H, Damm U, Wood AR, Shuttleworth LA, Hodges CS, Munster M, de Jesús Y-MM, Zúñiga-Estrada L, Cruywagen EM, de Hoog GS, Silvera C, Najafzadeh J, Davison EM, Davison PJN, Barrett MD, Barrett RL, Manamgoda DS, Minnis AM, Kleczewski NM, Flory SL, Castlebury LA, Clay K, Hyde KD, Maússe-Sitoe SND, Chen SF, Lechat C, Hairaud M, Lesage-Meessen L, Pawlowska J, Wilk M, Śliwińska-Wyrzychowska A, Mętrak M, Wrzosek M, Pavlic-Zupanc D, Maleme HM, Slippers B, Mac Cormack WP, Archuby DI, Grünwald NJ, Tellería MT, Dueñas M, Martín MP, Marincowitz S, de Beer ZW, Perez CA, Gené J, Marin-Felix Y, Groenewald JZ (2013). Fungal planet description sheets: 154–213. Persoonia.

[CR27] Cruywagen EM, Slippers B, Roux J, Wingfield MJ (2017). Phylogenetic species recognition and hybridisation in *Lasiodiplodia*: a case study on species from baobabs. Fungal Biology.

[CR28] Damm U, Crous PW, Fourie PH (2007). *Botryosphaeriaceae* as potential pathogens of *Prunus* species in South Africa, with descriptions of *Diplodia africana* and *Lasiodiplodia plurivora* sp. nov. Mycologia.

[CR29] Darriba D, Taboada GL, Doallo R, Posada D (2012). jModelTest 2: more models, new heuristics and parallel computing. Nature Methods.

[CR30] Denman S, Crous PW, Groenewald JZ, Slippers B, Wingfield BD, Wingfield MJ (2003). Circumscription of *Botryosphaeria* species associated with *Proteaceae* based on morphology and DNA sequence data. Mycologia.

[CR31] Denman S, Crous PW, Taylor JE, Kang J-C, Pascoe I, Wingfield MJ (2000). An overview of the taxonomic history of *Botryosphaeria* and a re-evaluation of ITS anamorphs based on morphology and ITS rDNA phylogeny. Studies in Mycology.

[CR32] Dissanayake AJ, Phillips AJL, Li XH, Hyde KD (2016). *Botryosphaeriaceae*: current status of genera and species. Mycosphere.

[CR33] Dissanayake AJ, Zhang W, Mei L, Chukeatirote E, Yan JY, Li XH, Hyde KD (2015). Lasiodiplodia pseudotheobromae causes pedicel and peduncle discolouration of grapes in China. Australasian Plant Disease Notes.

[CR34] Dou ZP, He W, Zhang Y (2017). *Lasiodiplodia chinensis*, a new holomorphic species from China. Mycosphere.

[CR35] Dou ZP, He W, Zhang Y (2017). Does morphology matter in taxonomy of *Lasiodiplodia*? An answer from *Lasiodiplodia hyalina* sp. nov. Mycosphere.

[CR36] Fang XM, Zeng YL, Li ZJ, Li SJ, Zhu TH (2019). First report of *Neofusicoccum* parvum associated with blotch trunk disease of *Koelreuteria paniculata* in China. Plant Disease.

[CR37] Farr DF, Elliott M, Rossman AY, Edmonds RL (2005). *Fusicoccum arbuti* sp. nov. causing cankers on *Pacific madrone* in western North America with notes on *Fusicoccum dimidiatum*, the correct name for *Scytalidium dimidiatum* and *Nattrassia mangiferae*. Mycologia.

[CR38] Guindon S, Dufayard J-F, Lefort V, Anisimova M, Hordijk W, Gascuel O (2010). New algorithms and methods to estimate maximum-likelihood phylogenies: assessing the performance of PhyML 3.0. Systematic Biology.

[CR39] Hillis DM, Bull JJ (1993). An empirical test of bootstrapping as a method for assessing confidence in phylogenetic analysis. Systematic Biology.

[CR40] IBM Corp (ed) (2011) IBM SPSS statistics for windows, version 20.0. IBM Corp, Armonk

[CR41] Inderbitzin P, Bostock RM, Trouillas FP, Michailides TJ (2010). A six locus phylogeny reveals high species diversity in *Botryosphaeriaceae* from California almond. Mycologia.

[CR42] Jami F, Marincowitz S, Slippers B, Wingfield MJ (2018). New *Botryosphaeriales* on native red milkwood (*Mimusops caffra*). Australasian Plant Pathology.

[CR43] Jayawardena RS, Hyde KD, Jeewon R, Ghobad-Nejhad M, Wanasinghe DN, Liu N, Phillips AJL, Oliveira-Filho JRC, da Silva GA, Gibertoni TB, Abeywikrama P, Carris LM, Chethana KWT, Dissanayake AJ, Hongsanan S, Jayasiri SC, McTaggart AR, Perera RH, Phutthacharoen K, Savchenko KG, Shivas RG, Thongklang N, Dong W, Wei D, Wijayawardena NN, Kang JC (2019). One stop shop II: taxonomic update with molecular phylogeny for important phytopathogenic genera: 26–50 (2019). Fungal Diversity.

[CR44] Jayawardena RS, Hyde KD, McKenzie EHC, Jeewon R, Phillips AJL, Perera RH, de Silva NI, Maharachchikumburua SSN, Samarakoon MC, Ekanayake AH, Tennakoon DS, Dissanayake AJ, Norphanphoun C, Lin C, Manawasinghe IS, Tian Q, Brahmanage R, Chomnunti P, Hongsanan S, Jayasiri SC, Halleen F, Bhunjun CS, Karunarathna A, Wang Y (2019). One stop shop III: taxonomic update with molecular phylogeny for important phytopathogenic genera: 51–75 (2019). Fungal Diversity.

[CR45] Jiang N, Wang X, Liang Y, Tian C (2018). *Lasiodiplodia cinnamomi* sp. nov. from *Cinnamomum camphora* in China. Mycotaxon.

[CR46] Katoh K, Standley DM (2013). MAFFT multiple sequence alignment software version 7: improvements in performance and usability. Molecular Biology and Evolution.

[CR47] Li GQ, Arnold RJ, Liu FF, Li JQ, Chen SF (2015). Identification and pathogenicity of *Lasiodiplodia* species from *Eucalyptus urophylla* × *grandis*, *Polyscias balfouriana* and *Bougainvillea spectabilis* in southern China. Journal of Phytopathology.

[CR48] Li GQ, Liu FF, Li JQ, Liu QL, Chen SF (2018). *Botryosphaeriaceae* from *Eucalyptus* plantations and adjacent plants in China. Persoonia.

[CR49] Li JQ, Wingfield MJ, Liu QL, Barnes I, Roux J, Lombard L, Crous PW, Chen SF (2017). *Calonectria* species isolated from *Eucalyptus* plantations and nurseries in South China. IMA Fungus.

[CR50] Li SB, Li JZ, Li SC, Lu ZH, Wang JH, Zhang H (2010). First report of *Neofusicoccum parvum* causing dieback disease of Chinese weeping cypress in China. Plant Disease.

[CR51] Linaldeddu BT, Deidda A, Scanu B, Franceschini A, Serra S, Berraf-Tebbal A, Zouaoui Boutiti M, Ben Jamâa ML, Phillips AJL (2015). Diversity of *Botryosphaeriaceae* species associated with grapevine and other woody hosts in Italy, Algeria and Tunisia, with descriptions of *Lasiodiplodia exigua* and *Lasiodiplodia mediterranea* sp. nov. Fungal Diversity.

[CR52] Liu JK, Phookamsak R, Doilom M, Wikee S, Li YM, Ariyawansha H, Boonmee S, Chomnunti P, Dai DQ, Bhat JD, Romero AI, Zhuang WY, Monkai J, Gareth Jones EB, Chukeatirote E, Ko TWK, Zhao YC, Wang Y, Hyde KD (2012). Towards a natural classification of *Botryosphaeriales*. Fungal Diversity.

[CR53] Liu QL, Li GQ, Li JQ, Chen SF (2016). *Botrytis eucalypti*, a novel species isolated from diseased *Eucalyptus* seedlings in South China. Mycological Progress.

[CR54] Liu YX, Shi YP, Deng YY, Li LL, Dai LM, Cai ZY (2017). First report of *Neofusicoccum parvum* causing rubber tree leaf spot in China. Plant Disease.

[CR55] Lombard L, Zhou XD, Crous PW, Wingfield BD, Wingfield MJ (2010). *Calonectria* species associated with cutting rot of *Eucalyptus*. Persoonia.

[CR56] Lopes A, Phillips AJL, Alves A (2017). Mating type genes in the genus *Neofusicoccum*: mating strategies and usefulness in species delimitation. Fungal Biology.

[CR57] Machado AR, Pinho DB, Pereira OL (2014). Phylogeny, identification and pathogenicity of the *Botryosphaeriaceae* associated with collar and root rot of the biofuel plant *Jatropha curcas* in Brazil, with a description of new species of *Lasiodiplodia*. Fungal Diversity.

[CR58] Manawasinghe IS, Phillips AJL, Hyde KD, Chethana KWT, Zhang W, Zhao WS, Yan JY, Li XH (2016). Mycosphere essays 14: assessing the aggressiveness of plant pathogenic *Botryosphaeriaceae*. Mycosphere.

[CR59] Marin-Felix Y, Groenewald JZ, Cai L, Chen Q, Marincowitz S, Barnes I, Bensch K, Braun U, Camporesi E, Damm U, de Beer ZW, Dissanayake A, Edwards J, Giraldo A, Hernández-Restrepo M, Hyde KD, Jayawardena RS, Lombard L, Luangsa-ard J, McTaggart AR, Rossman AY, Sandoval-Denis M, Shen M, Shivas RG, Tan YP, van der Linde EJ, Wingfield MJ, Wood AR, Zhang JQ, Zhang Y, Crous PW (2017). Genera of phytopathogenic fungi: GOPHY 1. Studies in Mycology.

[CR60] Marques MW, Lima NB, de Morais MA, Michereff SJ, Phillips AJL, Câmara MPS (2013). *Botryosphaeria*, *Neofusicoccum*, *Neoscytalidium* and *Pseudofusicoccum* species associated with mango in Brazil. Fungal Diversity.

[CR61] Mehl J, Wingfield M, Roux J, Slippers B (2017). Invasive everywhere? Phylogeographic analysis of the globally distributed tree pathogen *Lasiodiplodia theobromae*. Forests.

[CR62] Mohali S, Slippers B, Wingfield MJ (2006). Two new *Fusicoccum* species from *Acacia* and *Eucalyptus* in Venezuela, based on morphology and DNA sequence data. Mycological Research.

[CR63] Mohali SR, Slippers B, Wingfield MJ (2009). Pathogenicity of seven species of the *Botryosphaeriaceae* on *Eucalyptus* clones in Venezuela. Australasian Plant Pathology.

[CR64] NCBI Nucleotide Database. https://www.ncbi.nlm.nih.gov/nuccore/?term=Lasiodiplodia+pseudotheobromae+spacer. Accessed 3rd Dec 2019

[CR65] Netto MSB, Assunção IP, Lima GSA, Marques MW, Lima WG, Monteiro JHA, de Queiroz BV, Michereff SJ, Phillips AJL, Câmara MPS (2014). Species of *Lasiodiplodia* associated with papaya stem-end rot in Brazil. Fungal Diversity.

[CR66] Netto MSB, Lima WG, Correia KC, da Silva CFB, Thon M, Martins RB, Miller RNG, Michereff SJ, Câmara MPS (2017). Analysis of phylogeny, distribution, and pathogenicity of *Botryosphaeriaceae* species associated with gummosis of *Anacardium* in Brazil, with a new species of *Lasiodiplodia*. Fungal Biology.

[CR67] Old KM, Wingfield MJ, YuanZQ (ed) (2003) A manual of diseases of eucalypts in South-East Asia. Centre for International Forestry Research, Bogor

[CR68] Osorio JA, Crous CJ, de Beer ZW, Wingfield MJ, Roux J (2017). Endophytic *Botryosphaeriaceae*, including five new species, associated with mangrove trees in South Africa. Fungal Biology.

[CR69] Pavlic D, Slippers B, Coutinho TA, Gryzenhout M, Wingfield MJ (2004). *Lasiodiplodia gonubiensis* sp. nov., a new *Botryosphaeria* anamorph from native *Syzygium cordatum* in South Africa. Studies in Mycology.

[CR70] Pavlic D, Slippers B, Coutinho TA, Wingfield MJ (2009). Molecular and phenotypic characterization of three phylogenetic species discovered within the *Neofusicoccum parvum* / *N. ribis* complex. Mycologia.

[CR71] Pavlic D, Slippers B, Coutinho TA, Wingfield MJ (2009). Multiple gene genealogies and phenotypic data reveal cryptic species of the *Botryosphaeriaceae*: a case study on the *Neofusicoccum parvum* / *N. ribis* complex. Molecular Phylogenetics and Evolution.

[CR72] Pavlic D, Wingfield MJ, Barber P, Slippers B, Hardy GESJ, Burgess TI (2008). Seven new species of the *Botryosphaeriaceae* from baobab and other native trees in Western Australia. Mycologia.

[CR73] Pérez CA, Wingfield MJ, Slippers B, Altier NA, Blanchette RA (2010). Endophytic and canker-associated *Botryosphaeriaceae* occurring on non-native *Eucalyptus* and native *Myrtaceae* trees in Uruguay. Fungal Diversity.

[CR74] Phillips A, Alves A, Correia A, Luque J (2005). Two new species of *Botryosphaeira* with brown, 1-septate ascospores and *Dothiorella anamorphs*. Mycologia.

[CR75] Phillips AJL, Alves A, Abdollahzadeh J, Slippers B, Wingfield MJ, Groenewald JZ, Crous PW (2013). The *Botryosphaeriaceae*: genera and species known from culture. Studies in Mycology.

[CR76] Phillips AJL, Alves A, Pennycook SR, Johnston PR, Ramaley A, Akulov A, Crous PW (2008). Resolving the phylogenetic and taxonomic status of dark-spored teleomorph genera in the *Botryosphaeriaceae*. Persoonia.

[CR77] Phillips AJL, Hyde KD, Alves A, Liu JK (2019). Families in *Botryosphaeriales*: a phylogenetic, morphological and evolutionary perspective. Fungal Diversity.

[CR78] Phillips AJL, Oudemans PV, Correia A (2006). Characterisation and epitypification of *Botryosphaeria corticis*, the cause of blueberry cane canker. Fungal Diversity.

[CR79] Prasher IB, Singh G (2014) Lasiodiplodia indica - a new species of coelomycetous mitosporic fungus from India. KAVAKA 43:64–69

[CR80] Qi SX (2002). *Eucalyptus* in China.

[CR81] Rayner RW (1970). A mycological colour chart.

[CR82] Rodríguez-Gálvez E, Guerrero P, Barradas C, Crous PW, Alves A (2017). Phylogeny and pathogenicity of *Lasiodiplodia* species associated with dieback of mango in Peru. Fungal Biology.

[CR83] Sakalidis ML, Hardy GESJ, Burgess TI (2011). Use of the genealogical sorting index (GSI) to delineate species boundaries in the *Neofusicoccum parvum*–*Neofusicoccum ribis* species complex. Molecular Phylogenetics and Evolution.

[CR84] Sakalidis ML, Slippers B, Wingfield BD, Hardy GESJ, Burgess TI (2013). The challenge of understanding the origin, pathways and extent of fungal invasions: global populations of the *Neofusicoccum parvum*–*N. ribis* species complex. Diversity and Distributions.

[CR85] SAS Institute Inc (ed) (2011) SAS 9.3® system options: reference, Second edn. SAS Institute Inc, Cary

[CR86] Seifert KA, Rossman AY (2010). How to describe a new fungal species. IMA Fungus.

[CR87] Slippers B, Boissin E, Phillips AJL, Groenewald JZ, Lombard L, Wingfield MJ, Postma A, Burgess T, Crous PW (2013). Phylogenetic lineages in the *Botryosphaeriales*: a systematic and evolutionary framework. Studies in Mycology.

[CR88] Slippers B, Crous PW, Denman S, Coutinho TA, Wingfield BD, Wingfield MJ (2004). Combined multiple gene genealogies and phenotypic characters differentiate several species previously identified as *Botryosphaeria dothidea*. Mycologia.

[CR89] Slippers B, Crous PW, Jami F, Groenewald JZ, Wingfield MJ (2017). Diversity in the *Botryosphaeriales*: looking back, looking forward. Fungal Biology.

[CR90] Slippers B, Fourie G, Crous PW, Coutinho TA, Wingfield BD, Carnegie J, Wingfield MJ (2004). Speciation and distribution of *Botryosphaeria* spp. on native and introduced *Eucalyptus* trees in Australia and South Africa. Studies in Mycology.

[CR91] Slippers B, Fourie G, Crous PW, Coutinho TA, Wingfield BD, Wingfield MJ (2004). Multiple gene sequences delimit *Botryosphaeria australis* sp. nov. from *B. lutea*. Mycologia.

[CR92] Slippers B, Johnson GI, Crous PW, Coutinho TA, Wingfield BD, Wingfield MJ (2005). Phylogenetic and morphological re-evaluation of the *Botryosphaeria* species causing diseases of *Mangifera indica*. Mycologia.

[CR93] Slippers B, Roux J, Wingfield MJ, van der Walt FJJ, Jami F, Mehl JWM, Marais GJ (2014). Confronting the constraints of morphological taxonomy in the *Botryosphaeriales*. Persoonia.

[CR94] Slippers B, Wingfield MJ (2007). *Botryosphaeriaceae* as endophytes and latent pathogens of woody plants: diversity, ecology and impact. Fungal Biology Reviews.

[CR95] Smith H, Crous PW, Wingfield MJ, Coutinho TA, Wingfield BD (2001). *Botryosphaeria eucalyptorum* sp. nov., a new species in the *B. dothidea*-complex on *Eucalyptus* in South Africa. Mycologia.

[CR96] Smith H, Wingfield MJ, Crous PW, Coutinho TA (1996). *Sphaeropsis sapinea* and *Botryosphaeria dothidea* endophytic in *Pinus* spp. and *Eucalyptus* spp. in South Africa. South African Journal of Botany.

[CR97] Summerell BA, Groenewald JZ, Carnegie AJ, Summerbell RC, Crous PW (2006). *Eucalyptus* microfungi known from culture. 2. *Alysidiella*, *Fusculina* and *Phlogicylindrium* genera nova, with notes on some other poorly known taxa. Fungal Diversity.

[CR98] Swofford DL (2002). PAUP*. Phylogenetic analysis using parsimony (* and other methods).

[CR99] Tamura K, Stecher G, Peterson D, Filipski A, Kumar S (2013). MEGA6: molecular evolutionary genetics analysis version 6.0. Molecular Biology and Evolution.

[CR100] Taylor K, Barber PA, Hardy GE SJ, Burgess TI (2009). *Botryosphaeriaceae* from tuart (*Eucalyptus gomphocephala*) woodland, including descriptions of four new species. Mycological Research.

[CR101] Tennakoon DS, Phillips AJL, Phookamsak R, Ariyawansa HA, Bahkali AH, Hyde KD (2016). Sexual morph of *Lasiodiplodia pseudotheobromae* (*Botryosphaeriaceae*, *Botryosphaeriales*, *Dothideomycetes*) from China. Mycosphere.

[CR102] Tibpromma S, Hyde KD, McKenzie EHC, Bhat DJ, Phillips AJL, Wanasinghe DN, Samarakoon MC, Jayawardena RS, Dissanayake AJ, Tennakoon DS, Doilom M, Phookamsak R, Tang AMC, Xu J, Mortimer PE, Promputtha I, Maharachchikumbura SSN, Khan S, Karunarathna SC (2018). Fungal diversity notes 840–928: micro-fungi associated with *Pandanaceae*. Fungal Diversity.

[CR103] Trakunyingcharoen T, Lombard L, Groenewald JZ, Cheewangkoon R, To-anun C, Crous PW (2015). Caulicolous *Botryosphaeriales* from Thailand. Persoonia.

[CR104] Urbez-Torres JR, Peduto F, Striegler RK, Urrea-Romero KE, Rupe JC, Cartwright RD, Gubler WD (2012). Characterization of fungal pathogens associated with grapevine trunk diseases in Arkansas and Missouri. Fungal Diversity.

[CR105] van Burik JAH, Schreckhise RW, White TC, Bowden RA, Myerson D (1998). Comparison of six extraction techniques for isolation of DNA from filamentous fungi. Medical Mycology.

[CR106] van Niekerk JM, Crous PW, Groenewald JZ, Fourie PH, Halleen F (2004). DNA phylogeny, morphology and pathogenicity of *Botryosphaeria* species on grapevines. Mycologia.

[CR107] Velásquez AC, Castroverde CDM, He SY (2018). Plant and pathogen warfare under changing climate conditions. Current Biology.

[CR108] Wang W, Liu QL, Li GQ, Liu FF, Chen SF (2018). Phylogeny and pathogenicity of *Celoporthe* species from plantation *Eucalyptus* in southern China. Plant Disease.

[CR109] Wu RH, Zhang Y, Li ZP (2019). First report of leaf spot on rubber tree caused by *Lasiodiplodia pseudotheobromae* in China. Plant Disease.

[CR110] Xie YJ, Arnold RJ, Wu ZH, Chen SF, Du AP, Luo JZ (2017). Advances in eucalypt research in China. Frontiers of Agricultural Science & Engineering.

[CR111] Xu C, Wang C, Ju L, Zhang R, Biggs AR, Tanaka E, Li B, Sun G (2015). Multiple locus genealogies and phenotypic characters reappraise the causal agents of apple ring rot in China. Fungal Diversity.

[CR112] Yang T, Groenewald JZ, Cheewangkoon R, Jami F, Abdollahzadeh J, Lombard L, Crous PW (2017). Families, genera, and species of *Botryosphaeriales*. Fungal Biology.

[CR113] Ye W (2017). Lanscape and geography of YunNan Province.

[CR114] Yu L, Chen XL, Gao LL, Chen HR, Huang Q (2009). First report of *Botryosphaeria dothidea* causing canker and shoot blight of *Eucalyptus* in China. Plant Disease.

[CR115] Yu Z, Tang G, Peng S, Chen H, Zhai M (2015). *Neofusicoccum parvum* causing canker of seedlings of *Juglans regia* in China. Journal of Forestry Research.

[CR116] Zhai L, Zhang M (2019). First report of *Neofusicoccum parvum* causing fruit rot on *Eriobotrya japonica* in China. Plant Disease.

[CR117] Zhang M, Lin S, He W, Zhang Y (2017). Three species of *Neofusicoccum* (*Botryosphaeriaceae*, *Botryosphaeriales*) associated with woody plants from southern China. Mycosphere.

[CR118] Zhao JP, Lu Q, Liang J, Decock C, Zhang XY (2010). *Lasiodiplodia pseudotheobromae*, a new record of pathogenic fungus from some subtropical and tropical trees in southern China. Cryptogamie Mycologie.

[CR119] Zhou XD, de Beer ZW, Xie YJ, Pegg GS, Wingfield MJ (2007). DNA-based identification of *Quambalaria pitereka* causing severe leaf blight of *Corymbia citriodora* in China. Fungal Diversity.

[CR120] Zhou Y, Dou Z, He W, Zhang X, Zhang Y (2016). *Botryosphaeria sinensia* sp. nov., a new species from China. Phytotaxa.

[CR121] Zhou YP, Zhang M, Dou ZP, Zhang Y (2017). *Botryosphaeria rosaceae* sp. nov. and *B. ramosa*, new botryosphaeriaceous taxa from China. Mycosphere.

