# Supplementary material for: Variation in Botryosphaeriaceae from Eucalyptus plantations in YunNan Province in southwestern China across a climatic gradient
Source: IMA Fungus. 2020 Oct 15;11:22. doi: 10.1186/s43008-020-00043-x (PMC7560076; doi:10.1186/s43008-020-00043-x)
Supplement: Supplementary file 1 — Additional file 1: Figure S1. Symptoms observed on E. globulus and E. urophylla × E. grandis one month after inoculation. a, b. lesion produced on E. globulus by isolates (a) CSF6050 (L. pseudotheobromae) and (b) CSF5667 (N. parviconidium); c. negative control showing the absence of lesion development on E. globulus; d–k. lesion produced on E. urophylla × E. grandis by isolates (d) CSF5871 (B. fusispora), (e) CSF5820 (B. wangensis), (f) CSF5721 (N. dianense), (g) CSF5876 (N. magniconidium), (h) CSF6028 (N. ningerense), (i) CSF5667 (N. parviconidium), (j) CSF5782 (N. parvum), and (k) CSF5974 (N. yunnanense); l. negative control showing the absence of lesion development on E. urophylla × E. grandis. [file 43008_2020_43_MOESM1_ESM.pdf]

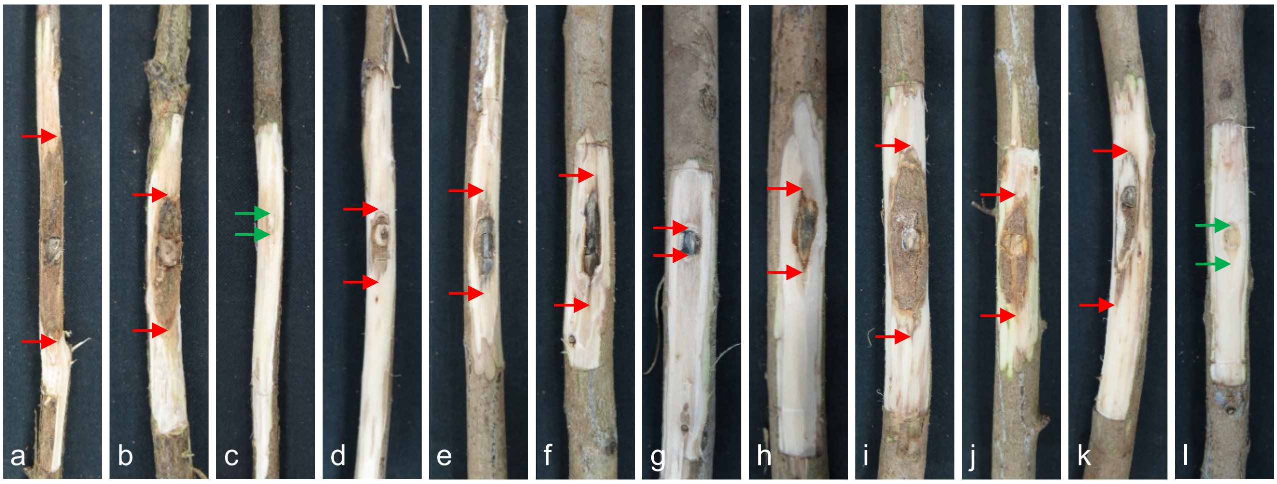

Fig. S1 Symptoms observed on *E. globulus* and *E. urophylla* × *E. grandis* one month after inoculation. **a, b**. lesion produced on *E. globulus* by isolates (a) CSF6050 (*L. pseudotheobromae*) and (b) CSF5667 (*N. parviconidium*); **c**. negative control showing the absence of lesion development on *E. globulus*; **d–k**. lesion produced on *E. urophylla* × *E. grandis* by isolates (d) CSF5871 (*B. fusispora*), (e) CSF5820 (*B. wangensis*), (f) CSF5721 (*N. dianense*), (g) CSF5876 (*N. magniconidium*), (h) CSF6028 (*N. ningerense*), (i) CSF5667 (*N. parviconidium*), (j) CSF5782 (*N. parvum*), and (k) CSF5974 (*N. yunnanense*); **l**. negative control showing the absence of lesion development on *E. urophylla* × *E. grandis*.
